# A noncanonical auxin-sensing mechanism is required for organ morphogenesis in *Arabidopsis*

**DOI:** 10.1101/gad.285361.116

**Published:** 2016-10-15

**Authors:** Sara Simonini, Joyita Deb, Laila Moubayidin, Pauline Stephenson, Manoj Valluru, Alejandra Freire-Rios, Karim Sorefan, Dolf Weijers, Jiří Friml, Lars Østergaard

**Affiliations:** 1 Department of Crop Genetics, John Innes Centre, Norwich NR4 7UH, United Kingdom;; 2 Department of Molecular Biology and Biotechnology, University of Sheffield, Sheffield S10 2TN, United Kingdom;; 3 Laboratory of Biochemistry, Wageningen University, 6703 HA Wageningen, the Netherlands;; 4 Institute of Science and Technology (IST) Austria, 3400 Klosterneuburg, Austria

**Keywords:** *Arabidopsis*, auxin signaling, IAA, plant development, transcription factor complex, ETTIN

## Abstract

Tissue patterning in multicellular organisms is the output of precise spatio–temporal regulation of gene expression coupled with changes in hormone dynamics. In plants, the hormone auxin regulates growth and development at every stage of a plant's life cycle. Auxin signaling occurs through binding of the auxin molecule to a TIR1/AFB F-box ubiquitin ligase, allowing interaction with Aux/IAA transcriptional repressor proteins. These are subsequently ubiquitinated and degraded via the 26S proteasome, leading to derepression of auxin response factors (ARFs). How auxin is able to elicit such a diverse range of developmental responses through a single signaling module has not yet been resolved. Here we present an alternative auxin-sensing mechanism in which the ARF ARF3/ETTIN controls gene expression through interactions with process-specific transcription factors. This noncanonical hormone-sensing mechanism exhibits strong preference for the naturally occurring auxin indole 3-acetic acid (IAA) and is important for coordinating growth and patterning in diverse developmental contexts such as gynoecium morphogenesis, lateral root emergence, ovule development, and primary branch formation. Disrupting this IAA-sensing ability induces morphological aberrations with consequences for plant fitness. Therefore, our findings introduce a novel transcription factor-based mechanism of hormone perception in plants.

## Abstract (precis)

Simonini et al. present an alternative auxin-sensing mechanism in which the auxin response factor ARF3/ETTIN controls gene expression through interactions with process-specific transcription factors.

Precise orchestration of organ patterning and polarity establishment during plant growth depends on close integration of environmental, hormonal, and cellular responses. A crucial factor facilitating such integration is the phytohormone auxin, which coordinates growth and development at every stage of a plant's life cycle (for review, see Benjamins and Scheres 2008; Vanneste and Friml 2009). Canonical auxin signaling occurs through binding of the auxin molecule to F-box proteins of the TIR1/AFB class, which forms part of the SCF^TIR1/AFB^ complex (Dharmasiri et al. 2005; Kepinski and Leyser 2005). This allows interaction with the transcriptional repressor proteins of the Aux/IAA family, which, in the absence of auxin, repress auxin response factor (ARF) proteins, preventing them from regulating their targets. The interaction between Aux/IAAs and ARFs occurs through the PB1 (Phox/Bem1p) domain, which is present in the C-terminal part of most ARFs (for review, see Guilfoyle 2015; Korasick et al. 2015; Salehin et al. 2015). Different combinations of the 23 ARFs, 29 Aux/IAAs, and six TIR1/AFB auxin receptors in *Arabidopsis* have been proposed to contribute to the complexity of auxin responses during plant development (Calderón Villalobos et al. 2012).

The *ETTIN* (*ETT*)/*ARF3* gene encodes an atypical ARF lacking the PB1 domain (Guilfoyle 2015), and it is therefore unclear how or whether ETT functions within the canonical auxin signaling machinery during plant development. Mutations in the *ETT* gene lead to severe polarity defects in the female reproductive organ, the gynoecium, with overproliferation of apical tissue and reduced ovary development (Fig. 1A,B; Supplemental Fig. 1A,B; Sessions et al. 1997). In addition to its role in the gynoecium, ETT functions during lateral root (LR) formation and ovule integument development, in the establishment of leaf polarity, and in the stem–pedicel fusion process (Garcia et al. 2006; Marin et al. 2010; Kelley et al. 2012; Zhao et al. 2013). It has been suggested that ETT has a role in the interpretation of auxin levels (Nemhauser et al. 2000; Pekker et al. 2005). However, a mechanistic model for how ETT may respond to changes in auxin concentrations has not yet been proposed.

**Figure 1.**
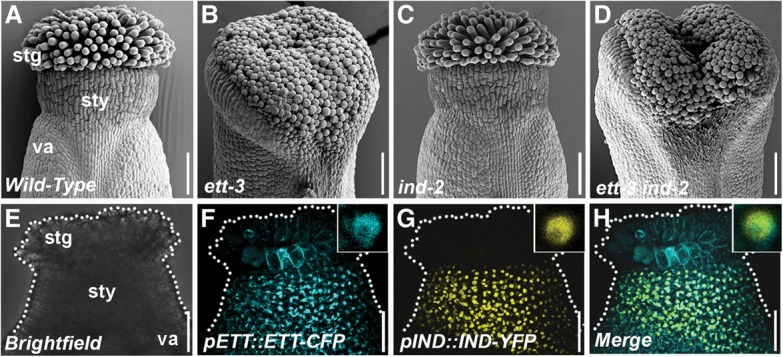
ETT and IND genetically interact to regulate gynoecium development. (*A*–*D*) Scanning electron micrographs (SEMs) of apices from stage 12 gynoecia of *Col-0* (*A*), *ett-3* (*B*), *ind-2* (*C*), and *ett-3 ind-2* (*D*). (*E–H*) Confocal images of stage 11 gynoecium (*E*) from double-transgenic lines expressing *pETT::ETT-CFP* (*F*) and *pIND::IND-YFP* (*G*) and their colocalization in the nucleus (*H*). *Insets* show close-ups of nuclei. (stg) Stigma; (sty) style; (va) valve. Bars, 100 µm.

INDEHISCENT (IND) is a basic helix–loop–helix (bHLH) transcription factor (TF) required for formation of the *Arabidopsis* valve margins, which comprise the tissue that allows the fruit to open upon seed dispersal (Liljegren et al. 2004). In addition, IND controls polarity at the apex of the gynoecium together with the bHLH protein SPATULA (SPT) (Girin et al. 2011; Moubayidin and Østergaard 2014), and, in both processes, IND mediates its function at least in part by controlling auxin distribution (Sorefan et al. 2009; Girin et al. 2011; Moubayidin and Østergaard 2014). IND coordinates directional auxin flux by direct repression of the *PINOID* (*PID*) gene (Sorefan et al. 2009). PID is a serine–threonine kinase that mediates polar auxin transport and is fundamental for proper symmetry establishment (Benjamins et al. 2001; Friml et al. 2004; Moubayidin and Østergaard 2014).

Here we show that ETT and IND interact to form a complex that mediates gynoecium patterning and that auxin affects the activity of this complex in the regulation of gene expression. Our data demonstrate the existence of a novel auxin-sensing mechanism comprised of ETT and IND that exhibits a strong preference for the naturally occurring auxin indole 3-acetic acid (IAA) over synthetic auxins and is fundamental for coordinating growth and patterning during carpel development. Moreover, our findings demonstrate that the IAA-sensing function is an intrinsic property of ETT, and the disruption of this IAA-sensing ability triggers morphological aberration in other developmental contexts in addition to gynoecium patterning such as LR emergence, ovule development, and primary branch formation. Thus, our findings introduce a novel TF-based mechanism of hormone signaling in plants.

## Results

### ETT and IND control polarity at the gynoecium apex by direct transcriptional regulation of the serine–threonine kinase PID

Understanding how auxin can coordinate growth with symmetry establishment and tissue patterning is essential to obtain an integrated comprehension of plant development. The *Arabidopsis* gynoecium is particularly well suited for such purposes due to its division into highly distinct tissues with different symmetries (for review, see Reyes-Olalde et al. 2013; Liu and Franks 2015). Both ETT and IND control polarity in the gynoecium through precise coordination of auxin distribution (Nemhauser et al. 2000; Girin et al. 2011). We therefore tested whether genetic interactions between them contribute to gynoecial polarity establishment. Although *ind-2* mutant gynoecia have no obvious radiality defects (Fig. 1A,C; Supplemental Fig. 1A,C,E,G), the radial style deformation of the *ett-3* single mutant is exacerbated in the *ett-3 ind-2* double mutant, which fails to achieve complete closure at the apex (Fig. 1A–D; Supplemental Fig. 1A–H), suggesting overlapping roles of these regulators. Analyses of the *pETT::ETT-CFP* and *pIND::IND-YFP* reporter lines revealed that *ETT* and *IND* expression patterns overlap in the apical region and that the fusion proteins colocalize in the nuclei in the cells of this domain (Fig. 1E–H; Supplemental Fig. 1I–K). We demonstrated previously that IND promotes radial symmetry at the gynoecium apex via interaction with another bHLH protein, SPT (Moubayidin and Østergaard 2014). The data presented here suggest that this may occur via cooperation with ETT. Indeed, a genetic interaction between *SPT* and *ETT* was reported previously (Heisler et al. 2001).

**Figure 2.**
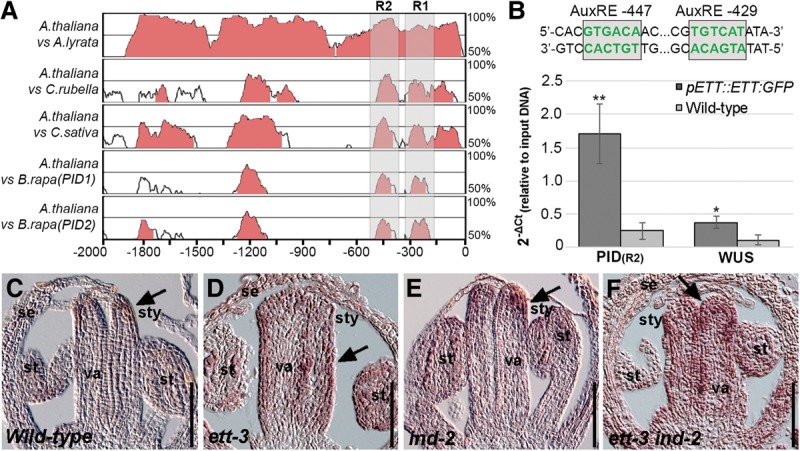
ETT and IND function together to regulate target genes during gynoecium development. (*A*) Phylogenetic shadowing using mVISTA of a 2-kb genomic region upstream of the translational start site of the *PID* gene with pairwise alignments of *Arabidopsis thaliana* with *Arabidopsis lyrata*, *Capsella rubella*, *Camelina sativa*, and *Brassica rapa*. Regions 1 and 2 (R1 and R2) are indicated by shaded areas. (*B*) ChIP with the *pETT::ETT-GFP* line showing enrichment of a fragment containing the conserved AuxRE sites at −429 and −447. The *WUS* promoter was used as a positive control. Error bars show standard deviation. (*) *P* < 0.01; (**) *P* < 0.001. (*C*–*F*) In situ hybridization of *PID* mRNA at the apex of stage 8 gynoecia from *Col-0* (*C*), *ett-3* (*D*), *ind-2* (*E*), and *ett-3 ind-2* (*F*). (se) Sepal; (st) stamen; (stg) stigma; (sty) style; (va) valve. Bars, 50 μm.

The controlled expression of *PID* at the gynoecium apex is fundamental to ensure proper symmetry establishment (Moubayidin and Østergaard 2014). Given that IND is a direct regulator of *PID* gene expression (Sorefan et al. 2009), we therefore tested whether ETT contributes to this regulation. The *PID* promoter contains a number of potential auxin response elements (AuxREs) with the consensus sequence TGTCNN (or NNGACA on the opposite strand). Phylogenetic shadowing using mVISTA (Mayor et al. 2000) demonstrated that two AuxREs in positions −429 and −447 compared with the start codon are highly conserved among *PID* genes in the *Brassicaceae* family and therefore are strong candidates for being recognition sites for ARF proteins (Fig. 2A,B). Chromatin immunoprecipitation (ChIP) using the *pETT::ETT-GFP* (*ett-3*) line revealed a particularly strong interaction with a region in the *PID* promoter containing these two elements (Fig. 2B; Supplemental Fig. 2A,B). Moreover, a yeast one-hybrid (Y1H) assay demonstrated that ETT specifically interacts with these sequences, while this interaction is disrupted when they are mutated to non-AuxREs (Supplemental Fig. 2C).

To test whether the interaction of ETT and IND with the *PID* promoter is of relevance for *PID* gene expression, we performed in situ hybridization with a probe against the *PID* mRNA on longitudinal sections of developing wild-type gynoecia. In agreement with previous data (Girin et al. 2011), we detected very weak *PID* expression at the apex of the gynoecium (Fig. 2C). This expression pattern was not significantly changed in the *ind-2* mutant but was markedly expanded in the *ett-3* mutant gynoecium (Fig. 2D,E). Moreover, in the *ett-3 ind-2* double mutant, strong ectopic and precocious *PID* expression was observed, extending the wild-type *PID* expression domain from the apex of the gynoecium into the carpel body (Fig. 2C,F). Analyses of the *pPID::PID-GUS* reporter line in an *ett-3 ind-2* background corroborated the in situ hybridization observations (Supplemental Fig. 2D–G) and together suggest that IND and ETT repress *PID* expression during early stages of gynoecium growth to ensure proper carpel development.

### ETT and IND proteins interact

**Figure 3.**
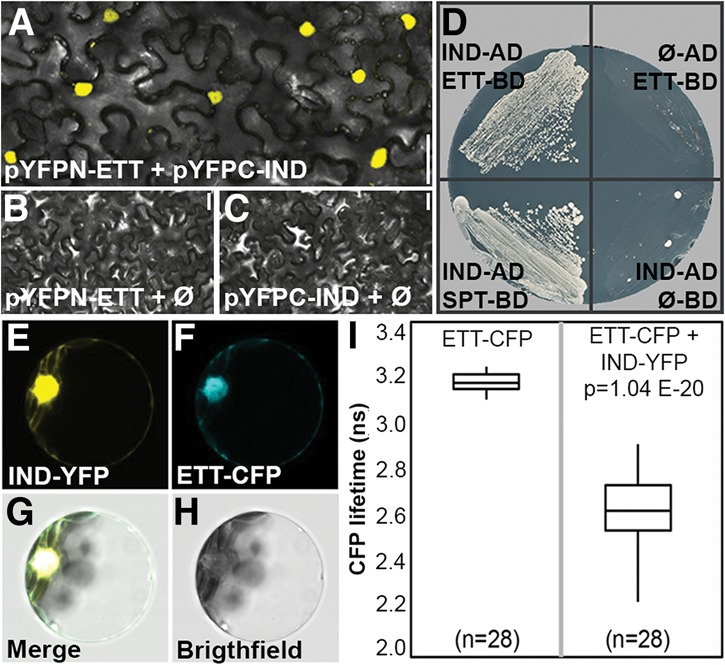
ETT and IND proteins interact. (*A*–*C*) BiFC with *pYFPN-ETT* and *pYFPC-IND* (*A*) and respective negative controls *pYFPN-ETT* + *pYPFC* empty (*B*) and *pYFPN* empty + *pYFPC-IND* (*C*). (*D*, *left*) Y2H assay showing positive interaction between IND-AD and ETT-BD and between IND-AD and SPT-BD. (*Right*) Negative controls (IND-AD with empty BD vector and ETT-BD with empty AD vector) are shown. (*E*–*H*) FRET/FLIM analysis in *Arabidopsis* protoplast of IND-YFP (*E*) and ETT-CFP (*F*) and the merged signal in the nucleus (*G*–*H*). (*I*) Quantification of CFP lifetime with ETT-CFP and empty YFP vector (*left*) and ETT-CFP with IND-YFP (*right*). Bars: *A*–*C*, 50 µm.

Given the genetic interaction between *ETT* and *IND*, their overlapping expression pattern, and the fact that both proteins together directly regulate *PID* expression, we investigated whether ETT and IND proteins interact. To this end, we used bimolecular fluorescence complementation (BiFC) (Fig. 3A–C), yeast two-hybrid (Y2H) assays (Fig. 3D), fluorescence resonance energy transfer/fluorescence lifetime imaging (FRET/FLIM) (Fig. 3E–I), and in vivo FRET (Supplemental Fig. 3A). All of the techniques revealed strong interactions between the two proteins, suggesting that ETT and IND most likely heterodimerize to regulate expression of their target genes.

A deletion analysis demonstrated that the C-terminal ETTIN-specific (ES) domain of 215 amino acids is sufficient for interaction with IND (Supplemental Fig. 3B). IND has been divided previously into three domains, including the bHLH domain in the C-terminal half and a central HEC domain with homology with the closest homologs of IND, the HECATE proteins (Girin et al. 2011; Gremski et al. 2007). The N-terminal part of IND, comprising 56 amino acids, has no sequence homology with any other protein in *Arabidopsis* and was therefore named the IND-specific (IS) domain. In contrast to ETT, all IND domains appear necessary to maintain the interaction with ETT (Supplemental Fig. 3B). Moreover, the interaction with IND was specific to ETT, since no interaction could be detected between IND and the ETT homolog ARF4 (Supplemental Fig. 3B). These results further support a synergistic role for ETT and IND in the control of gynoecium development.

### ETT and IND interaction is IAA-sensitive

**Figure 4.**
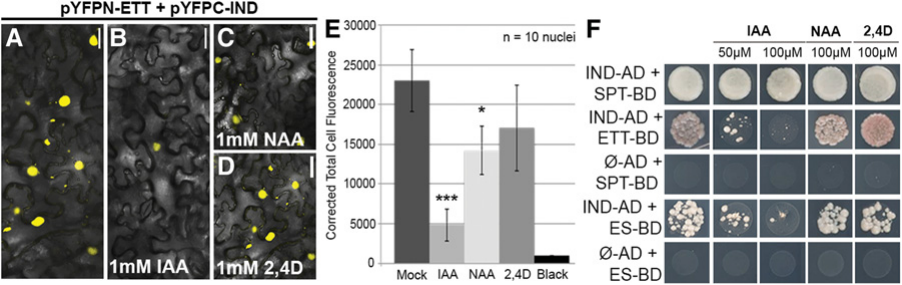
ETT and IND protein interact in an IAA-sensitive manner. (*A*–*D*) BiFC with *pYFPN-ETT* and *pYFPC-IND* in the presence of IAA (*B*), NAA (*C*), and 2,4-D (*D*). All hormonal treatments were with 1 mM in lanolin. (*E*) Fluorescence quantification (CTCF) of split YFP signal between ETT and IND without treatment and with IAA, NAA, and 2,4-D. (*) *P* < 0.01; (***) *P* < 0.0001. Error bars show the standard deviation. (*F*) Y2H assays with increasing concentration of IAA, NAA, and 2,4D. Bars: *A*–*D*, 50 µm.

Members of the ARF family are capable of forming complexes with components of the auxin signaling pathway, including other ARFs and Aux/IAA repressor proteins (Vernoux et al. 2011; Boer et al. 2014; Korasick et al. 2014). Since ETT does not contain a C-terminal PB1 domain and does not appear to interact with Aux/IAA proteins (Piya et al. 2014), it is possible that ETT is regulated post-transcriptionally by auxin via a different pathway. To test this, we added the naturally occurring auxin IAA to the in vivo interaction assays. Interestingly, we found that the YFP signal with ETT and IND in the BiFC assay decreased in the presence of IAA (applied in lanolin) (Fig. 4A,B,E; Supplemental Fig. 3C), suggesting that IAA affected the ETT–IND dimerization. Performing the Y2H assay in the presence of IAA showed, in agreement with the BiFC data, that the ETT–IND interaction was sensitive and suppressed at IAA concentrations similar to those used previously for testing the activity of TIR1 and AFBs in yeast (Fig. 4F; Calderón Villalobos et al. 2012). In contrast, the previously established interaction between IND and SPT was not sensitive to IAA (Fig. 4F). These data suggest that the dimerization between ETT and IND is modulated by IAA.

The effect on the ETT–IND complex was most pronounced with IAA, whereas the synthetic auxin NAA had a weaker effect in the BiFC assay. In yeast, neither NAA nor 2,4-D showed any effect (Fig. 4A–F). This IAA preference differs distinctly from the established SCF^TIR1/AFB^-mediated auxin signaling pathway that is strongly activated also by these synthetic analogs (Dharmasiri et al. 2005; Kepinski and Leyser 2005).

A potential biological consequence of the IAA-sensing mechanism emerged when we performed ChIP on ETT-GFP inflorescences following exogenous IAA treatment. While regions upstream in the *PID* promoter exhibited decreased association with ETT upon IAA treatment, a striking increase in the enrichment of a region close to the transcriptional start site of *PID* was detected (region 1) (Supplemental Fig. 4A,B). Although region 1 contains potential AuxREs, our phylogenetic shadowing analysis shows that none of these are conserved in *PID* genes of the other *Brassicaceae* species in the analysis. Moreover, the interaction between ETT and region 2 (containing the two conserved AuxREs) is not disrupted by the IAA treatment in both the ChIP and Y1H assays (Supplemental Fig. 4A,C). One way to explain this could be that the increased association of ETT with region 1 in the presence of IAA may be indirect and possibly occurs via another protein bound to this region and facilitated by the modified ETT–IND interaction (see the model in Supplemental Fig. 4B). The potential importance of sequences within region 1 for *PID* regulation is reflected in its high overall level of conservation in the phylogenetic shadowing analysis (Fig. 2A).

Additional support of IAA affecting IND activity emerged when we carried out a microarray experiment using a *35S::IND-GR* line treated with or without dexamethasone (DEX) and with or without IAA. In this analysis, 1734 genes significantly changed expression (>1.5 fold; *P* < 0.05) following induction of IND. However, out of these, 796 genes required the presence of IAA for IND to have an effect (Supplemental Fig. 5; Supplemental Table 1).

### IAA perception is fundamental for proper gynoecium morphogenesis

To assess the biological and functional relevance of the IAA-sensing ability in planta, we aimed to identify point mutations in IND and the ETT-ES domain that permitted the formation of the ETT–IND dimer while rendering it insensitive to IAA.

**Figure 5.**
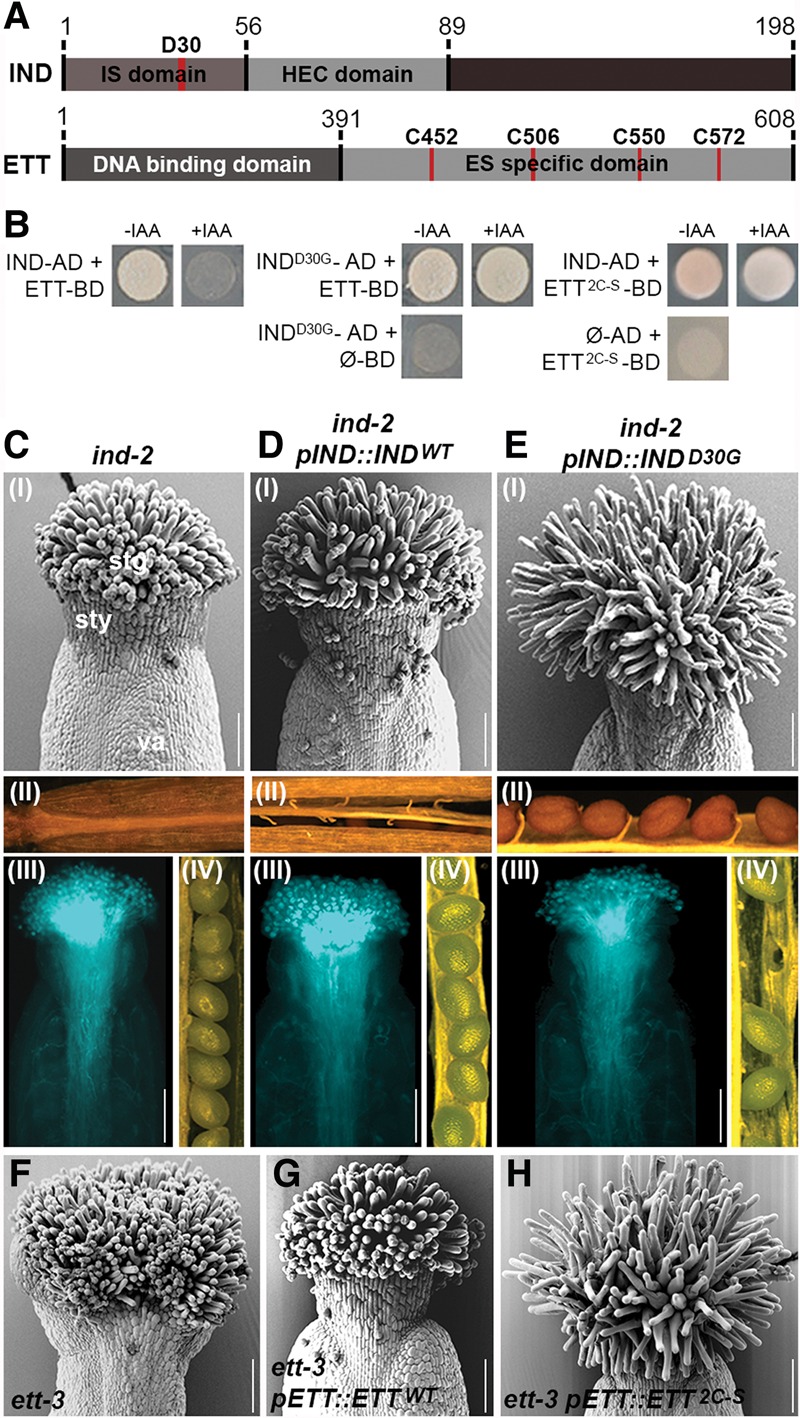
Auxin sensitivity is required for proper gynoecium morphogenesis. (*A*) Schematic representation of protein domains of IND and ETT. Numbers indicate amino acid positions. (*B*) Y2H assays of IND/IND^D30G^ versions with ETT-BD and ETT/ETT^2C-S^ versions with IND-AD and their sensitivity to IAA. Controls of IND^D30G^, ETT^2C-S^, and empty plasmids are shown *below*. (*C*–*E*) Phenotypic analyses of *ind-2* (*C*), *pIND::IND*^*WT*^ in *ind-2* (*D*), and *pIND::IND*^*D30G*^ in *ind-2* (*E*) with SEM images of stage 13 gynoecia (panel *I*), dehiscence (panel *II*), pollen tube growth (panel *III*), and a seed set (panel *IV*). (*F*–*H*) SEM images of stage 13 gynoecia from *ett-3* (*F*), *pETT::ETT*^*WT*^ in *ett-3* (*G*), and *pETT::ETT*^*2C-S*^ in *ett-3* (*H*). (stg) Stigma; (sty) style; (va) valve. Bars, 100 μm.

A screening for random point mutations introduced in the *IND* ORF resulted in the isolation of an aspartate-to-glycine substitution at position 30 (D30G) (Fig. 5A), which caused the ETT–IND interaction to become IAA-insensitive (Fig. 5B). Expressing this IND^D30G^ variant under the *IND* promoter (*pIND::IND*^*D30G*^) in the strong *ind-2* mutant background led to a striking overproliferation of stigmatic tissue accompanied by reduced pollen tube density and decreased fertility, which was not seen when *ind-2* was transformed with a wild-type *pIND::IND* construct or in the untransformed *ind-2* mutant (Fig. 5C–E; Supplemental Fig. 6A–F). These results strongly suggest that the IAA sensitivity of the ETT–IND complex is important for proper gynoecium development and plant reproduction.

In contrast to the gynoecium defect, expressing *pIND::IND*^*D30G*^ in the *ind-2* mutant background was perfectly able to rescue its indehiscent phenotype (Fig. 5E; Supplemental Fig. 6E). This observation shows that the IND^D30G^ protein is functional and that the IAA sensitivity of the IND and ETT interaction is not required for specification of the valve margin. This is in agreement with the lack of *ETT* expression in this tissue (Supplemental Fig. 1J).

Since both ETT and IND are TFs, we tested whether the IAA insensitivity of the ETT–IND^D30G^ complex affected the regulation of downstream target genes. In situ hybridization showed that *PID* expression was reduced in gynoecia of the *pIND::IND*^*D30G*^ line as compared with wild type (Supplemental Fig. 6G,H), thus suggesting that IAA regulates the activity of the ETT–IND complex toward its downstream targets. In the case of *PID* expression, it is conceivable that ETT–IND acts as a repressor in the absence of IAA, while accumulation of IAA leads to an ETT–IND-dependent induction of *PID* expression. Additional evidence of an ETT–IND-mediated effect on auxin dynamics in the gynoecium comes from a persisting DR5::GFP signal at the late developmental stages in the *pIND::IND*^*D30G*^ line (Supplemental Fig. 6I). This emphasizes a defect in auxin dynamics when disrupting the IAA-sensitive ETT–IND activity.

As opposed to the random mutagenesis approach with IND, we took a site-directed approach with ETT due to the presence of four cysteine residues in the ES domain. Cysteines have been shown previously to modulate transactivational activity of TFs (Klatt et al. 1999; Pineda-Molina et al. 2001; Dietz 2008; Li et al. 2009) and could therefore also play a role in the IAA-sensitive response uncovered here. We substituted each cysteine for serine and tested them individually in Y2H assays with wild-type IND. No single substitution interfered with the ETT–IND interaction but also did not render the interaction with IND IAA-insensitive (Supplemental Fig. 7A,B). In contrast, combining C-to-S mutations in positions 452 and 506 led to growth of yeast also in the presence of IAA (Fig. 5A,B). Remarkably, expressing this *pETT::ETT-C452S-C506S* IAA-insensitive variant (referred to here as *pETT::ETT*^*2C-S*^) in the *ett-3* mutant background led to the same overproliferated stigmatic tissue phenotype observed in the *pIND::IND*^*D30G*^ line (Fig. 5F–H), while other defects of the *ett-3* gynoecium were rescued (Supplemental Fig. 7C–H). These data therefore strongly support the hypothesis that gynoecium development requires the ETT–IND-mediated IAA-sensing pathway.

### ETT can interact with proteins belonging to different TF families in an IAA-sensitive fashion

**Figure 6.**
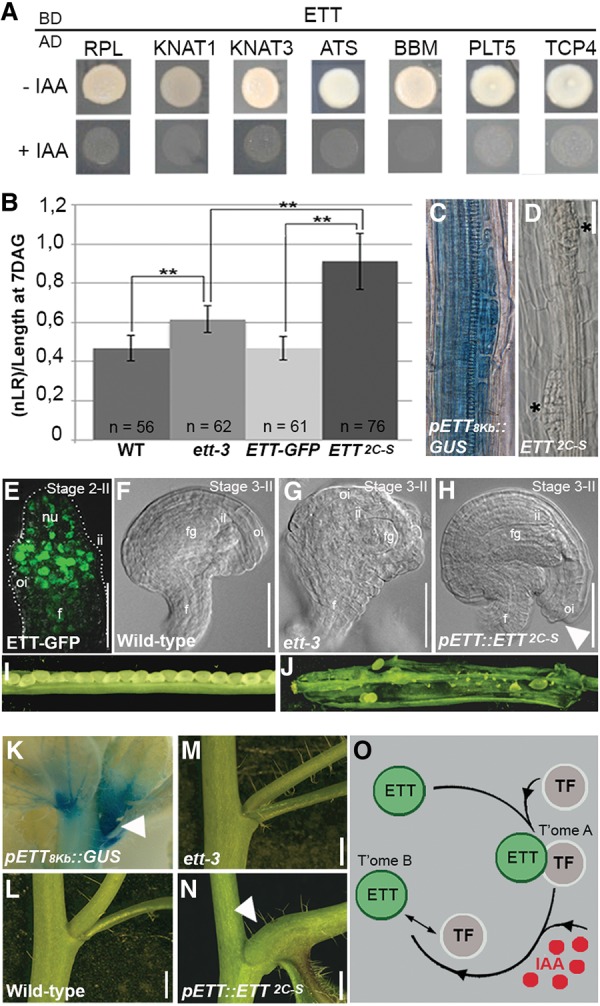
Auxin sensitivity is required for proper organ morphogenesis. (*A*) Y2H assay showing IAA sensitivity of interactions of ETT-BD and TFs (fused to the AD domain) identified in the REGIA (Regulatory Gene Initiative in *Arabidopsis*) library. Assays were carried out in the absence of IAA (−IAA; *top*) and presence of IAA (+IAA; *bottom*). (*B*–*D*) Chart summarizing the comparison of LR numbers among the genotypes (normalized against root length), showing a statistically higher number of LR in the *pETT::ETT*^*2C-S*^ line (shown in *B*). GUS staining of the *pETT(8Kb)::GUS* line in the root (*C*) and LR defects in *pETT::ETT*^*2C-S*^ (*D*), with two adjacent LRs developing (indicated by asterisks). (*E*) *pETT::ETT-GFP* expression profile in ovule primordia at stage 2-II; the signal is localized predominantly in the region from which the integuments will develop. (*F*–*H*) Clearing of wild-type (*F*), *ett-3* (*G*), and *pETT::ETT*^*2C-S*^ (*H*) ovules showing abnormal overgrowth of the outer integument in *pETT::ETT*^*2C-S*^. (*I*,*J*) Stage 17 fruits from wild type (*I*) and *pETT::ETT*^*2C-S*^ (*J*), with one valve removed to expose the defect in fertility in the *pETT::ETT*^*2C-S*^ line. (*K*–*N*) GUS staining of the *pETT(8Kb)::GUS* line during lateral branch formation (*K*) and close-up views of the primary branch–stem internode in wild type (*L*), *ett-3* (*M*), and *pETT::ETT*^*2C-S*^ (*N*). (*O*) Schematic model illustrating how the IAA-induced change in the ETT/TF complex dimerization state determines the regulation of downstream targets. As shown for *PID* in this study, transcriptomes A and B share genes regulated differently by the repressor/activator states. (nu) Nucellus; (ii) inner integument; (oi) outer integument; (fg) female gametophyte; (f) funiculus. Bars: *C–H*, 20 µm; *L–M*, 5 mm.

Abnormal carpel development is the most obvious and severe defect in *ett* mutants; however, ETT has been reported to function in a diverse range of developmental contexts in addition to the gynoecium; namely, LR initiation, ovule integument development, and stem–pedicel fusion (Marin et al. 2010; Kelley et al. 2012; Zhao et al. 2013). Analyses of the *pETT(8Kb)::GUS* reporter line (Ng et al. 2009) and the *pETT::ETT-GFP* complementation line confirmed the presence of *ETT* expression in domains relevant to these three processes. *ETT* expression was detected (1) in the pericycle (the tissue that will give rise to LRs) and during all stages of LR formation (Fig. 6C; Supplemental Fig. 8A,B); (2) during ovule development, coinciding with the region from which the integuments will later emerge (Fig. 6E; Supplemental Fig. 9A–F); and (3) at the abaxial side of the new developing primary branches, marking the region that corresponds to the point of conjunction between the stem and the branch (Fig. 6K; Supplemental Fig. 10A–C).

In contrast, IND function appears to be very specific to gynoecium and fruit development (Liljegren et al. 2004; Girin et al. 2011). It is therefore possible that ETT constitutes the principal prerequisite for the IAA-sensing ability and that it interacts with different protein partners in other processes. To test this hypothesis, we performed a Y2H screening of ETT against the REGIA (Regulatory Gene Initiative in *Arabidopsis*) library of TFs (Paz-Ares et al. 2002). This analysis led to the identification of several putative ETT interactors (Fig. 6A; Supplemental Fig. 11) belonging to different TF families, including HOMEOBOX (REPLUMLESS [RPL], KNOTTED-LIKE FROM ARABIDOPSIS THALIANA1 [KNAT1], and KNAT3), AP2 domain (BABYBOOM [BBM]), and TCP (TEOSINTE BRANCHED, CYCLOIDEA AND PCF4 [TCP4] and TCP18). Since ETT already has been reported to interact with ABERRANT TESTA SHAPE (ATS; also known as KANADI4) during ovule development (Kelley et al. 2012), ATS was included in this test. Furthermore, due to its high sequence homology with BBM, we also included PLETHORA5 (PLT5). Both ATS and PLT5 were found to interact with ETT in this assay (Fig. 6A). Similar to the interaction with IND, the interaction between ETT and these TFs was sensitive to the presence of IAA (Fig. 6A), thus suggesting that the IAA-sensitive activity of ETT involves tissue- and process-specific TF partners. Interestingly, the factors on this list function in the same processes as ETT, including the development of roots (KNAT3, BBM, and PLT5) (Serikawa et al. 1997; Galinha et al. 2007; Truernit and Haseloff 2007; Hofhuis et al. 2013), ovules (ATS) (Kelley et al. 2012), stem–pedicel fusion (TCP18) (Aguilar-Martínez et al. 2007), and carpels (RPL and KNAT1) (Roeder et al. 2003; Alonso-Cantabrana et al. 2007). Remarkably, RPL was shown recently to directly bind a fragment of the *PID* promoter located ∼1.5 kb upstream of the *PID* start codon (Bencivenga et al. 2016). This region is enriched in the ETT-GFP ChIP assay (region 4 in Supplemental Fig. 2A,B) despite the lack of conserved AuxREs (Fig. 2A). As proposed above, it is thus possible that ETT associates with DNA in this region indirectly via IAA-controlled protein–protein interactions (see the model in Supplemental Fig. 4B).

We next wondered whether the IAA-sensing activity of ETT is a generally conserved mechanism for modulating organ and tissue morphogenesis in response to high IAA concentrations. Indeed, LR initiation, ovule integument development, and primary branch fusion are strictly connected to and dependent on auxin accumulation (Benková et al. 2003; Gallavotti 2013). Therefore, we performed a morphological analysis and compared wild-type, *ett-3*, *pETT::ETT-GFP*, and *pETT::ETT*^*2C-S*^ genotypes with respect to these three different developmental contexts.

In accordance with the *ETT* expression pattern, the *ett-3* mutant displays defects in all the three tissues: a significantly higher number of developing LRs (Fig. 6B; Supplemental Fig. 8C–F), aberrant ovule integument morphology (Fig. 6F,G; Supplemental Fig. 9K–N; Kelley et al. 2012), and a mild deformity at the stem–primary branch fusion (Fig. 6L,M; Supplemental Fig. 10D,E,H,I; Zhao et al. 2013). Notably, the *pETT::ETT-GFP* construct was able to complement all of these defects (Fig. 6B; Supplemental Figs. 8E,F, 9O,R, 10F,J). In contrast, analyses of the *pETT::ETT*^*2C-S*^ line revealed morphological aberrations in all three developmental contexts that could not be explained by complementation of the *ett-3* phenotypes.

First, during the LR emergence process, we detected a significant overproduction of LRs along the root length and in proximity to the root tip (Fig. 6B,D; Supplemental Fig. 8C–F). Second, during ovule development, we observed an extensive overgrowth of the outer integument that brings it particularly close to the funiculus (Fig. 6H; Supplemental Fig. 9S–V), thus placing the micropile aperture, the point of entrance of the pollen tube, in an unfavorable position for fertilization. As a consequence, *pETT::ETT*^*2C-S*^ plants displayed very high ovule abortion frequency and a drastically reduced seed set (Fig. 6I,J). Last, in *pETT::ETT*^*2C-S*^ plants, abnormal development at the point of conjunction between the stem and the primary branch was observed (Fig. 6N), with the latter developing more horizontally in comparison with wild type (Supplemental Fig. 10D–K). These results demonstrate that ETT-mediated IAA perception plays multiple diverse roles throughout plant development.

## Discussion

A common theme in plant hormone signaling has emerged involving ubiquitination and degradation of transcriptional repressors (for review, see Santner and Estelle 2009). For auxin, this mechanism involves repression of ARFs at low auxin concentrations through interaction between PB1 protein domains of ARFs and Aux/IAAs. Derepression of ARFs at high auxin levels occurs when the Aux/IAA repressor proteins are degraded (for review, see Guilfoyle 2015). ETT belongs to the ARF family and has been associated previously with auxin-related processes during gynoecium development (Nemhauser et al. 2000); however, given that ETT lacks the PB1 domain, it seems unlikely that ETT would function via the canonical auxin signaling pathway. Indeed, the data presented here demonstrate the existence of an alternative mechanism through which IAA regulates ETT activity. This mechanism is fundamentally different from the canonical auxin signaling pathway, as it involves an IAA-mediated direct effect on TF activity without the involvement of a ubiquitination and degradation step. Both the ETT–TF and TIR1/AFB pathways are short, allowing for rapid transcriptional responses to auxin. However, since the Aux/IAAs must be resynthesized to reset repression, the ETT–TF pathway may have an advantage in the speed with which it can return to a nonauxin state, depending on the reversibility of the IAA effect. This may be particularly important during processes of developmental switching such as in the patterning of the gynoecium apex or the initiation of LRs.

Our data suggest that IAA affects the activity of ETT toward downstream targets by directly interfering with ETT–TF interactions (Fig. 6O). Specific ETT–TF complexes may therefore control two separate transcriptomes dependent on the absence/presence of IAA (Fig. 6O, transcriptomes A and B, respectively). The same genes may be present in both transcriptomes but simply be controlled differently under the two conditions. One such example is *PID*: At low IAA levels, an ETT–IND complex represses *PID* expression, whereas high IAA levels change the manner in which ETT interacts with the *PID* promoter, most likely via IAA-induced changes in protein interactions correlating with induction of *PID* expression (Supplemental Fig. 4A,B). The IAA-dependent modulation of ETT protein interactions implicates that, in a precise spatio–temporal window that coincides with *ETT* expression, a specific set of ETT partners must be available to achieve a tissue-specific transcriptional response. The identification here of a number of proteins from diverse TF families that interact with ETT in an IAA-sensitive manner supports this hypothesis.

The genetic results as well as the protein–protein interaction data indicate that IAA directly affects the ETT–TF dimer, opening the possibility that the IAA perception occurs via direct contact between the IAA molecule and the ETT protein. If so, this mechanism may be reminiscent of the way by which the thyroid hormone receptor (TR) regulates its targets in animals (for review, see Tsai and O'Malley 1994; Xu et al. 1999). The TR is constitutively bound to the promoter of its target genes. However, in the absence of the hormone ligand, TR functions primarily as a repressor, whereas binding of the ligand changes it into an activator of the same targets (Tsai and O'Malley 1994; Xu et al. 1999). The molecular nature of the interaction between ETT and IAA and the effect it has on the ETT–TF complex will be the focus of future biochemical and structural studies.

In summary, we identified a novel IAA-sensing mechanism in plant development. While adding to the complexity of auxin signaling, these findings will contribute to our understanding of how auxin can regulate such a vast number of biological processes. Above and beyond the specific mechanism by which the ETT–TF sensor complex controls organ morphology, identification of this novel signaling conduit may have far-reaching implications for the existence of similar alternative mechanisms for hormonal regulation of plant growth and development.

## Materials and methods

### Plant materials and growth conditions

Plants were grown on soil in long-day conditions (16 h light/8 h dark). Mutant line *ind-2* is a strong loss-of-function allele in the Col-0 background (Liljegren et al. 2004); *ett-3*, a mutant allele of medium strength and originally in the Ler ecotype (Sessions et al. 1997), was introgressed in the *Col-0* background via five backcrosses; and *pETT(8Kb)::GUS* was kindly provided by Toshiro Ito (Ng et al. 2009). Selection of transgenic lines was carried out by plating seeds on 0.8% MS supplemented with the appropriate antibiotic; the plates were subsequently stratified for 4 d in the dark at 4°C and then moved to long-day conditions (16 h light/8 h dark).

### Scanning electron micrographs (SEMs), sectioning, and GUS staining

SEMs and sectioning were performed as described previously (Moubayidin and Østergaard 2014). GUS staining was performed as described previously (Liljegren et al. 2004). SEM images were used as the source for stigmatic papilla length quantification.

### ChIP

ChIP was performed in triplicate according to Schiessl et al. (2014) using *pETT::ETT-GFP* in the *ett-3* line, and wild-type plants were used as a negative control. Data were analyzed as in Schiessl et al. (2014). A WUS promoter fragment was used as a positive control as described previously (Liu et al. 2014). IAA treatment was performed by spraying *pETT::ETT-GFP* and wild-type plants with a solution containing 0.1 mM IAA, 10 µM NPA, and 0.03% silwet and collecting material 6 h after treatment. Primers sequences are in Supplemental Table 2.

### Y1H assay, Y2H assay, and REGIA Y2H library screening

The Y1H assay was performed in strains Y187 and PJ64a following the protocol described in Roccaro et al. (2005). *PID* promoter fragments were cloned in the pHISi vector (Clontech). Interaction was tested on selective yeast synthetic dropout (YSD) medium lacking Leu (L) and His (H) supplemented with different concentrations (20, 40, and 60 mM) of 3-aminotriazole (3-AT). IAA (Sigma) was dissolved in ethanol and added at the desired concentration directly to the cooling medium.

The Y2H assays were performed at 28°C in the yeast strain AH109 (Clontech) using the cotransformation technique (Egea-Cortines et al. 1999). Coding sequences were cloned into pGAD424 and pGBKT9 vectors (Clontech) using the SmaI and PstI sites or cloned into the Gateway vector GAL4 system (pGADT7 and pGBKT7; Clontech) passing through pDONR207 (Life Technologies). Strength of interaction was tested on selective YSD lacking Leu (L), Trp (W), adenine (A), and His (H) supplemented with different concentrations (1, 2.5, and 5 mM) of 3-AT. IAA, NAA, and 2,4D (Sigma) were dissolved in ethanol and added at the desired concentrations directly to the cooling medium.

Screening of the REGIA library was performed with the mating technique; the ETT coding sequence was cloned in the pDEST32 plasmid (Invitrogen) passing through the pDONR207 (Invitrogen) and transformed in the PJ69a strain. The REGIA library was in the PJ69alfa mating type. After mating, cells were resuspended in sterile water and plated on YSD − W-L-H-A supplemented with different concentration of 3-AT. Plasmids were extracted from positive clones and sequenced.

### BiFC

ORFs of full-length IND and ETT were cloned in the pYFPN43 and pYFPC43 (http://www.ibmcp.upv.es/ferrandolabvectors.php) passing through pDONR207 (Life Technologies). The pEAQ-HT vector (Sainsbury et al. 2009), which expresses the silencing suppressor *p19* of tomato bushy stunt virus, was added to the vector combination. The positive *pYFPN43-SNF* + *pYFPC43-SNF* control was used as described previously (Ferrando et al. 2001). BiFC was performed as described previously (Belda-Palazón et al. 2012).

Exogenous IAA, NAA, and 2,4D applications were performed by mixing lanolin with the hormone as described previously (Reinhardt et al. 2003); a thin layer of lanolin–hormone mixture was applied on the infiltrated area 24 and 48 h after infiltration. Images were taken 72 h after infiltration.

### In situ hybridization

The *PID* digoxygenin-labeled antisense and sense RNA probes were generated by in vitro transcription according to the instructions provided with the DIG RNA labeling kit (SP6/T7; Roche) using cDNA as template. Developing inflorescences were fixed and embedded in Paraplast Plus embedding medium, cut in 8-μm sections, and then hybridized as described previously (Dreni et al. 2007). For in situ of the *ind-2 pIND-IND*^*D30G*^ line, a 3× *PID* antisense probe quantity was used to amplify the signal. Sections were observed using a Leica DM6000 equipped with differential interface contrast (DIC) optics.

### FRET/FLIM

FRET/FLIM was performed as described previously (Boer et al. 2014) using transfected *Arabidopsis* (Columbia wild-type) mesophyll protoplasts (Rademacher et al. 2012). Images of 128 × 128 pixels were acquired with acquisition times of 120 sec and an average count rate of 104 photons per second. Analysis of the data was as described (Rademacher et al. 2012). Several cells (*n* = 28) were analyzed, and average FLIMs of different combinations were exported for generating a box plot. The statistical significance of differences between samples was determined using a two-tailed Student's *t*-test.

### Phylogenetic footprinting

Phylogenetic shadowing was performed using the mVISTA program (http://genome.lbl.gov/vista/index.shtml). *PID* genomic sequences from *Brassicaceae* species were obtained from public databases via BLAST search on http://www.ncbi.nlm.nih.gov using the *Arabidopsis PID* gene as the query sequence.

### Microarray data analyses using GeneSpring

35S::IND:GR seeds were grown for 7 d in 5 mL of 0.5% (w/v) glucose, 0.5× Murashige, and Skoog medium with constant shaking and constant light. Seedlings were treated for 6 h with either DMSO mock or 10 μM IAA ± 10 μM DEX. RNA was extracted from three biological replicates (total of 12 samples). The Robust Multiarray Average (RMA) algorithm was used to normalize the microarray data using Affymetrix expression console. Affymetrix transcriptome analysis console (TAC) software was used for differential gene expression analysis. Genes with a >1.5-fold differential expression and a *P*-value of <0.05 were selected using one-way ANOVA.

### Pollen tube guidance

Pistils were emasculated and pollinated after 24 h with wild-type pollen. After 16–18 h, pistils were carefully isolated from the plants and fixed in a solution of acetic acid and absolute ethanol (1:3), cleared with 8 M NaOH, and labeled with aniline blue (Sigma). Images were taken using a Leica DM6000.

### In planta colocalization and in vivo CFP–YFP FRET

*pIND (3.2kb)::IND-YFP* and *pETT(3.7kb)::ETT-CFP* constructs were assembled using the Golden Gate method and appropriate plasmids as described previously (Engler et al. 2008, 2014) and introduced in wild-type plants. Samples was imaged with a Zeiss LSM 780 confocal microscope. YFP fluorescence was excited using the 514-nm line from an argon ion laser, and the emitted light was captured between 530 and 579 nm. CFP fluorescence was excited using the 458-nm line from an argon ion laser, and the emitted light was captured between 470 and 535 nm. Confocal Z-section stacks were collected at 1-µm spacing throughout the depth of the tissue. In vivo FRET was performed with a Zeiss LSM 780 confocal microscope following the acceptor photobleaching method reported in Karpova and McNally (2006). Two-hundred nuclei were imaged for the ETT-CFP/IND-YFP-expressing cells (stylar cells at stage 10–11). As a control, 200 nuclei from a tissue expressing ETT:CFP only (valves at stages 4–5) were imaged. The background value was subtracted from all the values.

### ETT constructs and marker line

For the *pETT::ETT-GFP* marker line, the ETT gene (including a 5-kb promoter sequence upstream of the ATG exons and introns) was cloned in the pPZP222 plasmid already harboring the GFP-coding region and the tNOS terminator and transformed in *ett-3* (Col-0 background). Thirteen independent T1 lines were analyzed for GFP expression pattern. Quantitative real-time PCR analysis was used to estimate the numbers of transgene copies in these individual lines, similar to the approach reported previously (Bartlett et al. 2008). Line number 1, which contained one copy of the transgene and fully complemented the *ett-3* phenotype, was propagated to obtain *ett-3* homozygous plants with the homozygous *pETT::ETT-GFP* construct.

For construction of ETT versions with cysteines mutated to serines, the *ETT* gene (including a 5-kb promoter sequence upstream of the ATG, exons, and introns) was cloned in the pPZP222 plasmid already harboring a tNOS terminator and transformed in *ett-3* (*Col-0* background). Cysteine-to-serine mutations were introduced by site-specific mutagenesis. Primer sequences are in Supplemental Table 2.

### Error-prone mutagenesis for IND^D30G^ identification

Error-prone PCR mix was composed of G2 polymerase buffer (Promega) and G2 polymerase, 5 mM MgCl_2_, 0.15 mM MnCl_2_, and unbalanced dNTP mix (2.5 mM dATP, 2.5 mM dGTP, 10 mM dCTP, 10 mM dTTP). The pool of PCR fragments was incubated with the pGADT7-rec plasmid (Clontech) following the cotransformation protocol described in the Matchmaker library construction and screening user manual (Clontech). Cells were plated on YSD-selective medium lacking Trp (W), Leu (L), Ade (A), and His (H) and supplemented with 100 μM IAA. Plasmids were recovered from those colonies able to grow in the presence of IAA and sequenced (total of 27 colonies). One clone carried five mismatches, including the D30G mutation, and three had a deletion of 12 amino acids, including the D30 residue. The remaining 23 clones contained several errors that affected the correct frame and for this reason were discarded. The *IND* promoter (3.2 kb upstream of the ATG) plus the IND/IND^D30G^-coding region were cloned into the pCGN1547 vector already harboring the tNOS terminator. The *Agrobacterium tumefaciens* strain GV3101 containing the final vector was transformed into *ind-2* plants using the floral dip method.

## Supplementary Material

Supplemental Material
