# Supplementary material for: A noncanonical auxin-sensing mechanism is required for organ morphogenesis in Arabidopsis
Source: Genes Dev. 2016 Oct 15;30(20):2286–96. doi: 10.1101/gad.285361.116 (PMC5110995; doi:10.1101/gad.285361.116)
Supplement: Supplemental Material [file supp_30_20_2286__index.html]

Supplemental Material 

# A noncanonical auxin-sensing mechanism is required for organ morphogenesis in *Arabidopsis*

## Supplemental Material

**Files in this Data Supplement:**

- Supplemental\_Material.pdf
- Supplemental\_Table\_S1.pdf
