# Supplementary material for: A noncanonical auxin-sensing mechanism is required for organ morphogenesis in Arabidopsis
Source: Genes Dev. 2016 Oct 15;30(20):2286–96. doi: 10.1101/gad.285361.116 (PMC5110995; doi:10.1101/gad.285361.116)
Supplement: Supplemental Material [file supp_30.20.2286_Supplemental_Material.pdf]

Supplemental Figure 1.

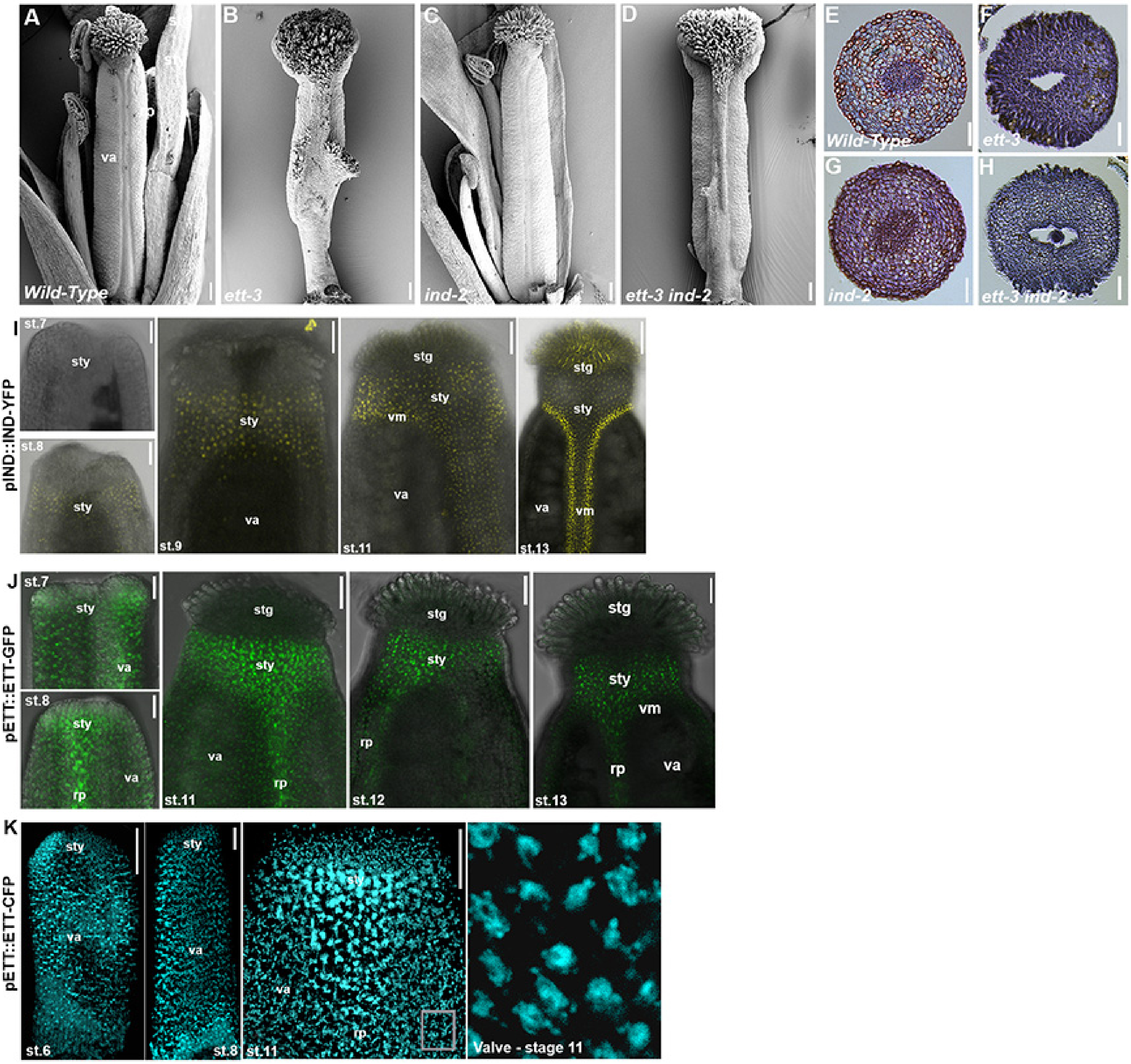

Supplemental Figure 1.

(A–D) Scanning electron microscopy (SEMs) of whole stage-14 gynoecia of Col-0 (A), *ett-3* (B), *ind-2* (C) and *ett-3 ind-2* (D).

(E-H) Toluidin Blue-stained cross sections of styles from Col-0 (E), *ett-3* (F), *ind-2* (G) and *ett-3 ind-2* (H).

(I) *pIND::IND-YFP* expression from stage 7 to stage-13 of gynoecium development.

(I) *pIND::IND-YFP* expression from stage 7 to stage-13 of gynoecium development.

(J) *pETT::ETT-GFP* expression from stage 7 to stage 13 of gynoecium development. No GFP expresson can be detected in the valve margin tissue.

(K) *pETT::ETT-CFP* expression at stage 6, 8 and 11 of gynoecium development. Last image is a magnification of a portion of the valve showing nuclear localization of the ETT-CFP protein.

rp, replum; vm, valve margin; va, valve; stg, stigma; sty, style.

Scale bars, 100 µm

Supplemental Figure 2.

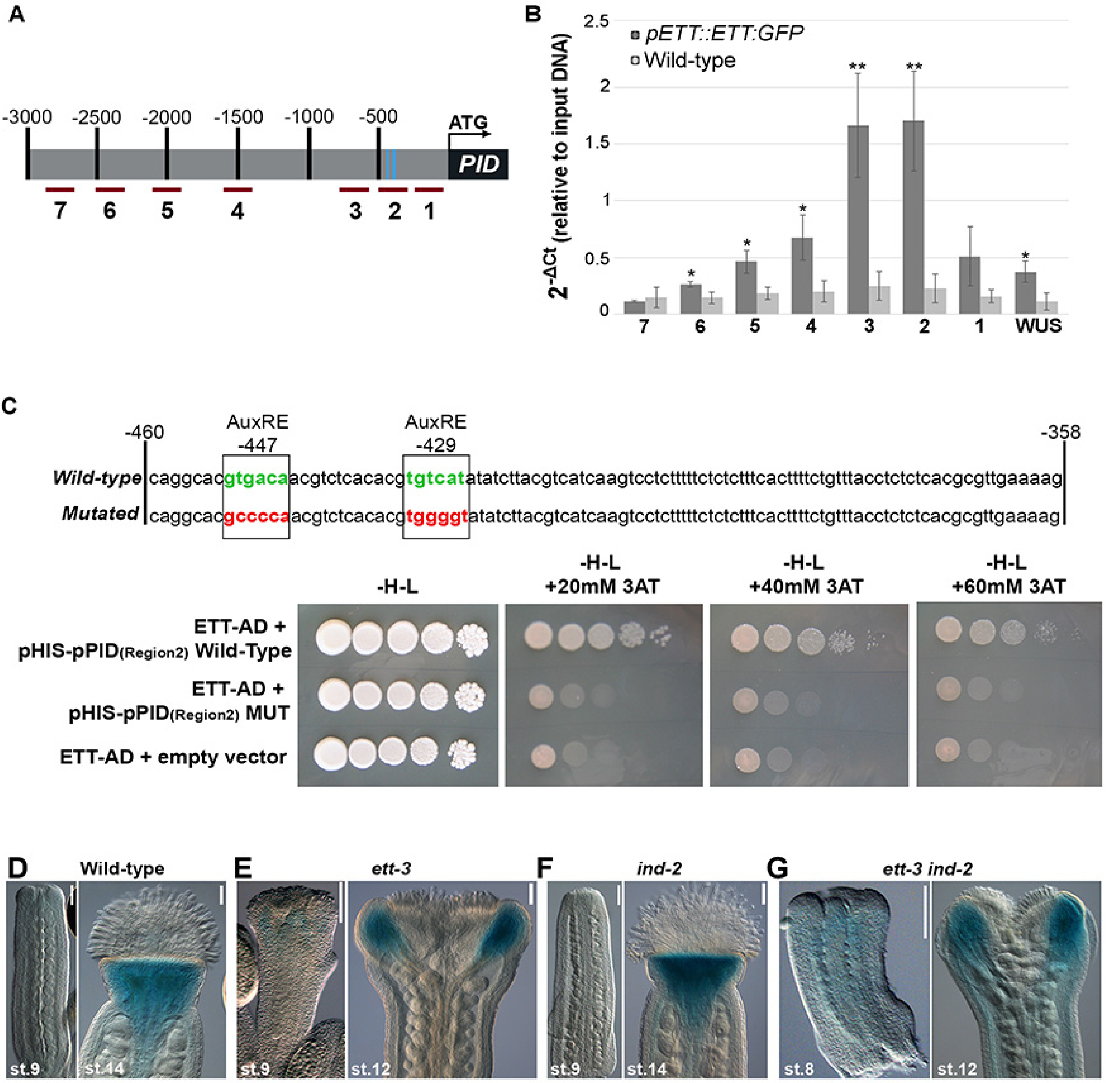

Supplemental Figure 2.
(A) Schematic representation of the five fragments in the *PID* promoter tested in ChIP assay. *PID* coding region is depicted in black. Blue bars represent the conserved AuxRE sites -429 and -447.
(B) Chromatin immunoprecipitation (ChIP) with *pETT::ETT-GFP* line showing enrichment in fragments 1, 2, 3 and 4 of *PID* promoter. *WUS* promoter is used as positive control. Standard deviations are indicated. * indicates $p<0.01$ and ** indicates $p<0.001$.
(C) Yeast-one-hybrid assay with ETT-AD vector in combination with wild-type and mutated version of the Region 2 of PID promoter containing the conserved AuxRE site at -429 and -447.
(D-G) GUS staining of gynoecium at stage 8-9 (left) and stage 12-14 (right) of *pPID::PID-GUS* line in Col-0 (D), *ett-3* (E), *ind-2* (F) and *ett-3 ind-2* (G).
rp, replum; vm, valve margin; va, valve; stg, stigma; sty, style.
Scale bars, 100 µm

Supplemental Figure 3.

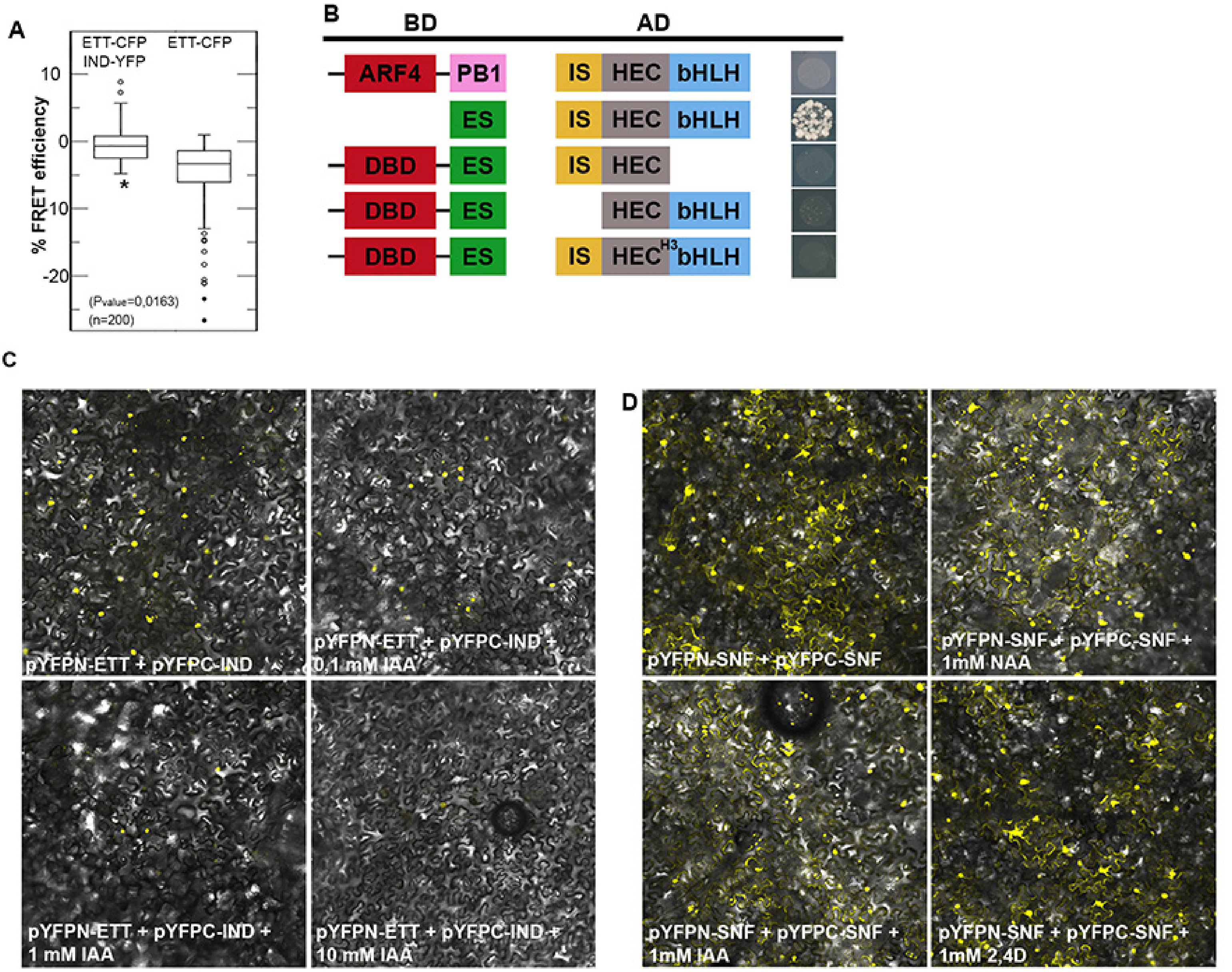

Supplemental Figure 3.

(A) *in planta* FRET analyses with percentage FRET efficiency with *pETT::ETT-CFP* and *pIND::IND-YFP* (left) and *pETT::ETT-CFP* alone (right).

(B) Yeast two-hybrid assays. Upper row shows no interaction between ARF4 and IND.Below, assays with truncated versions of IND and ETT. The ETT-specific domain (ES) and the entire IND protein are necessary for the interaction. ETT domains: DBD, DNA-binding Domain; ES, ETT-specific domain. IND domains: IS, IND-specific domain; HEC, HECATE domain; bHLH, basic Helix-Loop-Helix domain. The HECH3 domain refers to the HEC domain from HECATE3. BD and AD are Binding Domains and Activation Domain from the yeast 2-hybrid system, respectively.

(C) BiFC assay showing decreasing signal of *pYFPN43-IND* + *pYFPC-ETT* after exogenous IAA application.

(D) BiFC assay between SNF proteins as positive control for interaction. None of the hormonal treatments affect the interaction.

Supplemental Figure 4.

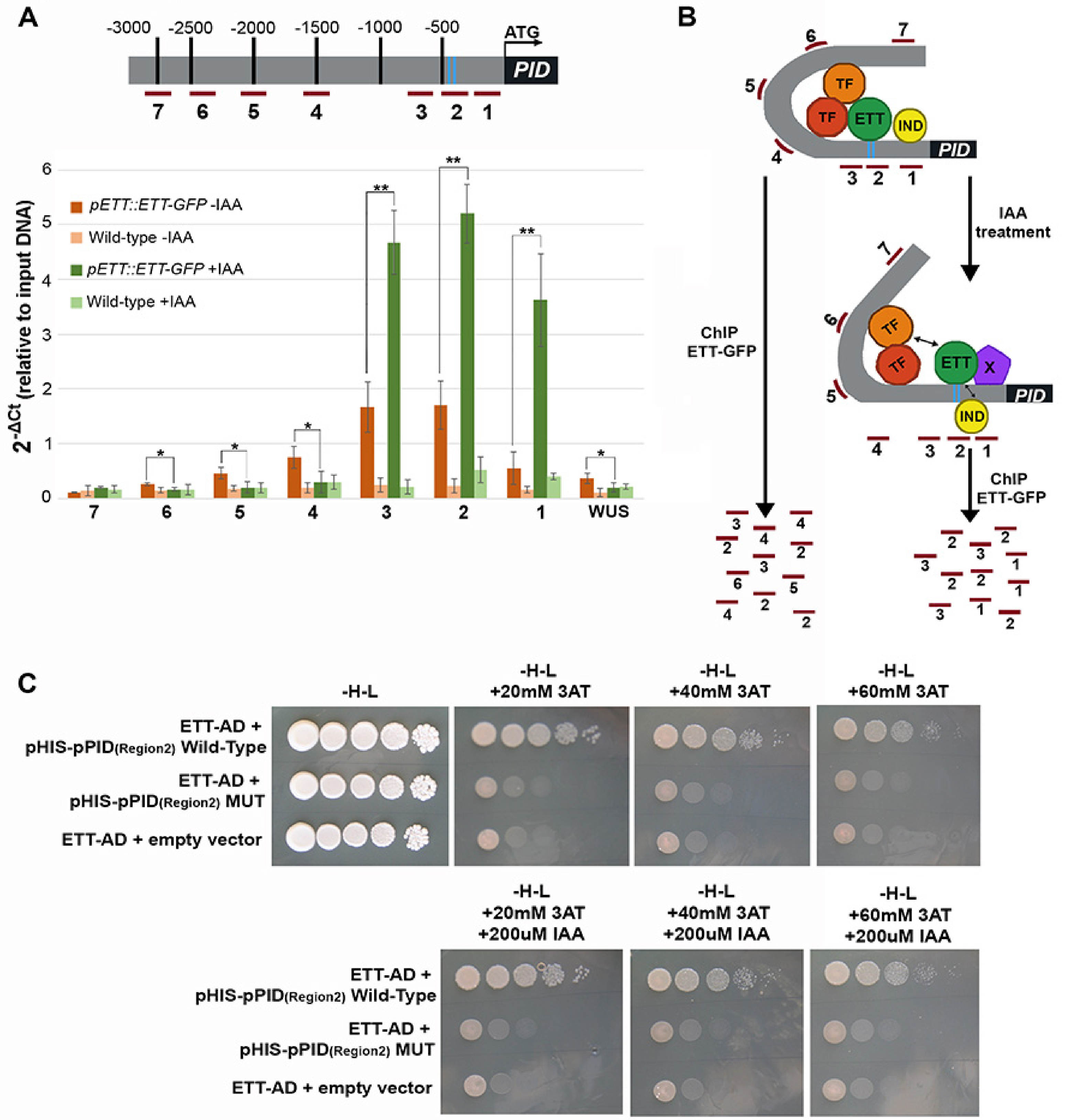

Supplemental Figure 4.

(A) (A) Chromatin Immunoprecipitation (ChIP) in absence (red and orange bars) and presence (dark and light green bars) of IAA. The enrichment in fragments 4-5-6 of the *PID* promoter without IAA is strongly reduced in presence of IAA. Increament in enrichment is observed at sites 1-2-3. The enrichment of the *WUS* promoter fragment, which is included as a positive control in the ChIP experiment without IAA is no longer enriched in the presence of IAA. Error bars show standard deviations. * indicates $p<0.01$ and ** indicates $p<0.001$.

(B) Graphic representation of proposed model for ETT dynamics on PID promoter. In absence of IAA, ETT is bound to the region 2 (and 3) of PID promoter through direct contact with the AuxRE -429 and -447. We propose that interaction of ETT with protein partners allows immunoprecipitation of regions 4-5-6, which do not contain any conserved AuxRE. When IAA cellular concentration increases, ETT dimerization with its partner is abolished thus allowing immunoprecipitation of fragments belonging to the region proximal to the PID start site. Additional protein-protein interactions, stimulated by IAA addiction, would allow the enrichment of the region 1.

(C) Yeast-one-hyrbid assay in absence (top panel) and presence (bottom panel) of IAA between ETT and a fragment of *PID* promoter containing the AuxRE sites -4279 and -447.

Supplemental Figure 5.

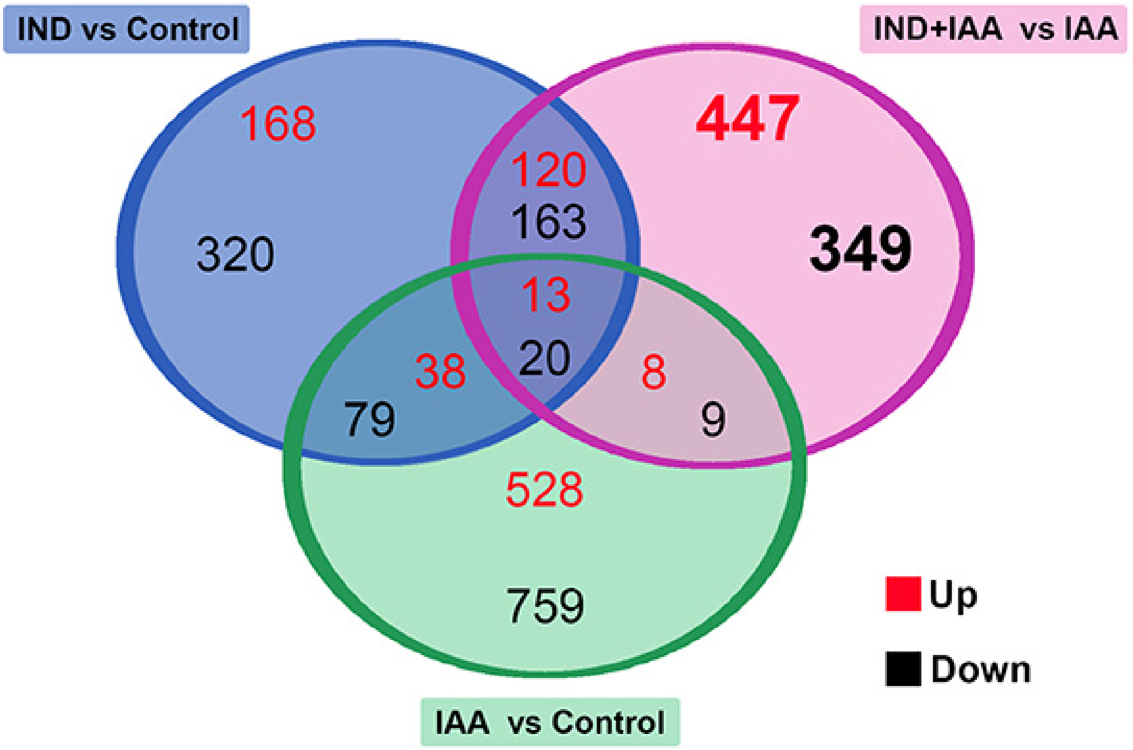

Supplemental Figure 5.
Venn diagram of micro-array experiment using the Affymetrix 22K Ath1 chip. The 35S::IND:GR line was used as previously described (14). Comparisons were made between 10 μM Dex-induction of IND versus control (IND vs control), 50 μM IAA versus control (IAA vs control) and Dex+IAA versus IAA (IND+IAA vs control). Numbers in red indicate upregulated genes and black indicate downregulated genes. The numbers in bigger font indicate the 796 genes (447 up + 349 down) that are regulated by IND only in the presence of IAA.

Supplemental Figure 6.

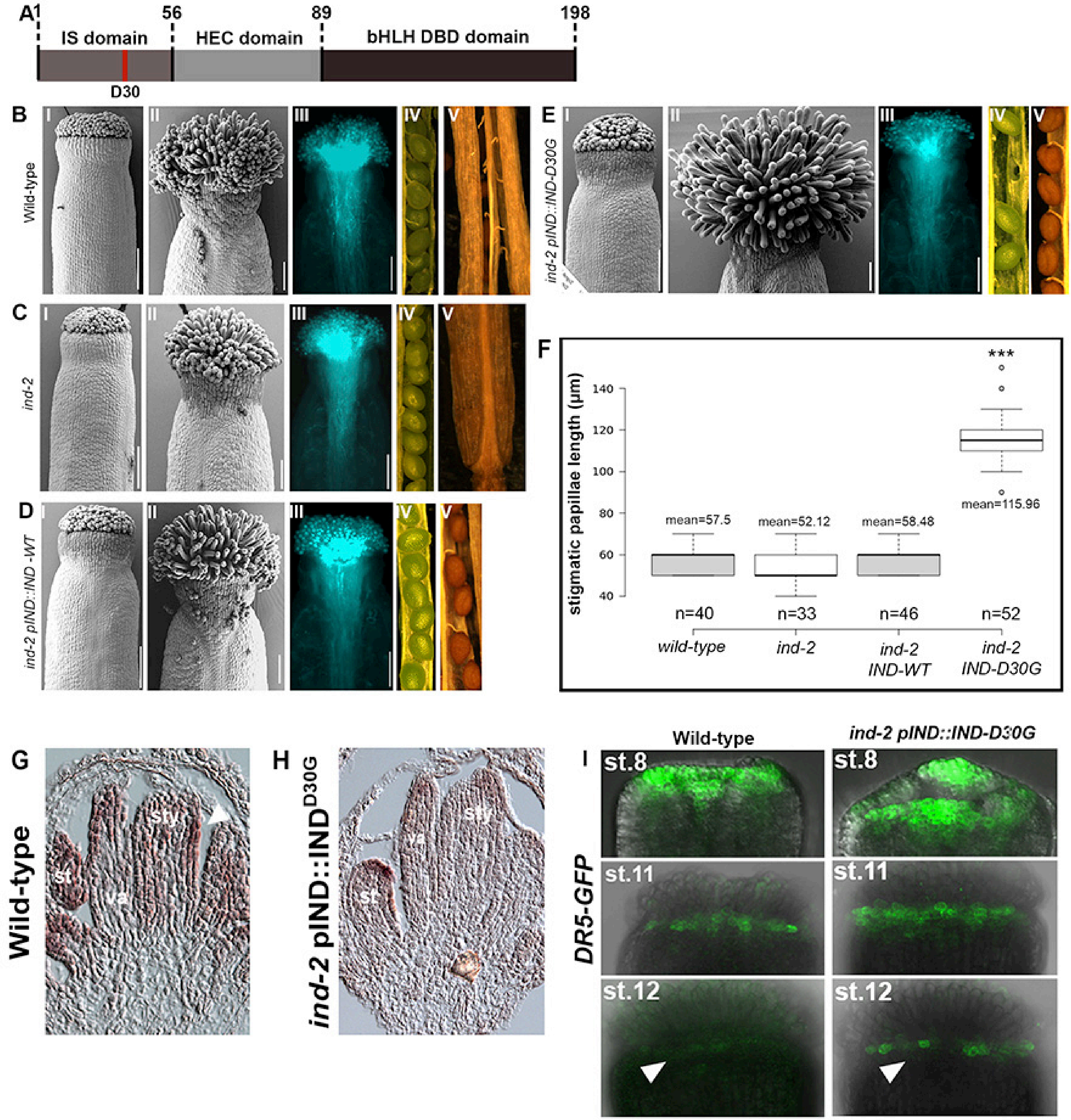

Supplemental Figure 6.

(A) Schematic representation of IND protein domains with position D30 in red.

(B-E) Phenotypical analyses of *pIND::IND-WT* (D) and *pIND::IND-D30G* lines (E) in *ind-2* background (C). Pollen tube density analyses (III image) showed reduce pollen tube growth in *pIND::IND-D30G line* (E) when compared with Wild-type (B), *ind-2* (C) and *pIND::IND-WT* (D). Reduce pollen tube density is accompanied by a reduced fertility with evident ovule abortion (IV image). *pIND::IND-D30G* was able to complement indehiscence phenotype of *ind-2* valves (V image).

(F) Quantification of stigmatic papillae length in wild-type, *ind-2*, *ind-2 pIND-IND-WT* and *ind-2 pIND-IND-D30G* plants. Data are analyzed with One-Way Anova; *** indicates $p<0.0001$.

(H-G) *PID in* situ hybridisation with 3x probe concentration compared to Fig. 1K-N in order reveal the reduced PID expression in the gynoecium (stage 9) of *ind-2 pIND::IND-D30G* (G) compared to wild type (H). Notice that the reduced expression is specific to the gynoecium as *PID* expression in stamens is similar between wild type and mutant. White arrows point to *PID* expression in the apical regions of the gynoecia.

(H-I) DR5-GFP confocal analyses of gynoceia at stage 8, 11 and 12 of wild-type and *pIND::IND-D30G* mutant. White arrowhead points to a persistent DR5-GFP signal, not visible in wild-type plants, at the top of gynoecia in the ind-2 pIND::IND-D30G line.

st: stamen, sty: style, va: valve. Scale bars, 100µm

Supplemental Figure 7.

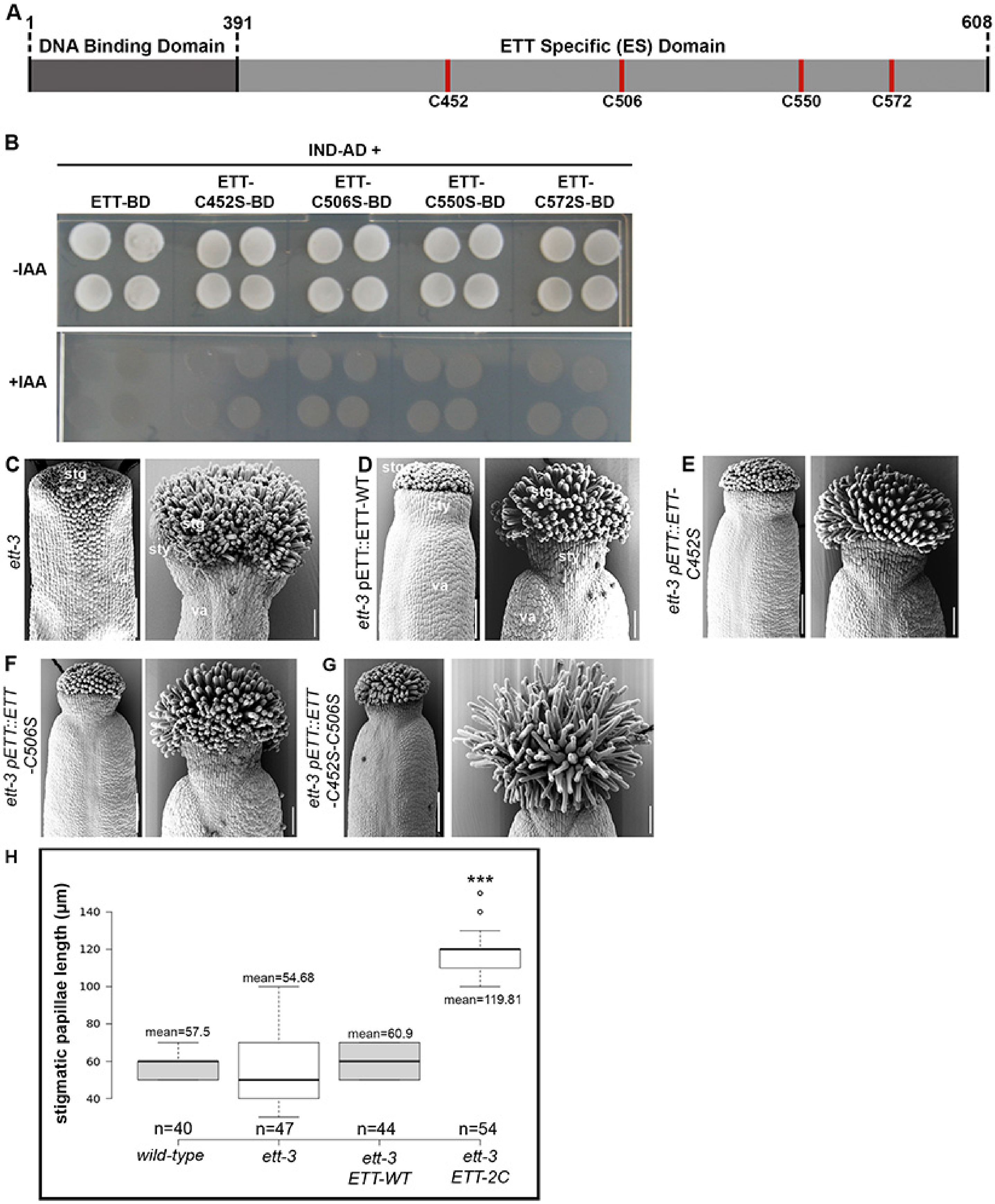

Supplemental Figure 7.

(A) Schematic representation of ETT protein domains and positions of Cysteine residues in the ETT-ES domain.

(B) Yeast 2-hybrid assay shown that individually mutating the cysteines to serine has no effect on the sensitivity of ETT to IAA in the interaction with IND.

(C-G) Phenotypical analyses by SEM of gynoecia from *ett-3* (C), *pETT::ETT-WT* in *ett-3* (D) *pETT::ETT-C452S* in *ett-3* (E), *pETT::ETT-C506S* in *ett-3* (F), and *pETT::ETT-2C-S* (G). Gynoecia were analysed at stage 11 (left image) and 13 (right image) of carpel development.

(H) Quantification of stigmatic papillae length in wild-type, *ett-3*, *ett-3 pETT::ETT-WT* and *ett-3 pETT::;ETT-2C* plants. Data are analyzed with One-Way Anova; *** indicates $p<0.0001$.

stg: stigma; sty: style; va: valve.

Scale bars, 100μm.

Supplemental Figure 8.

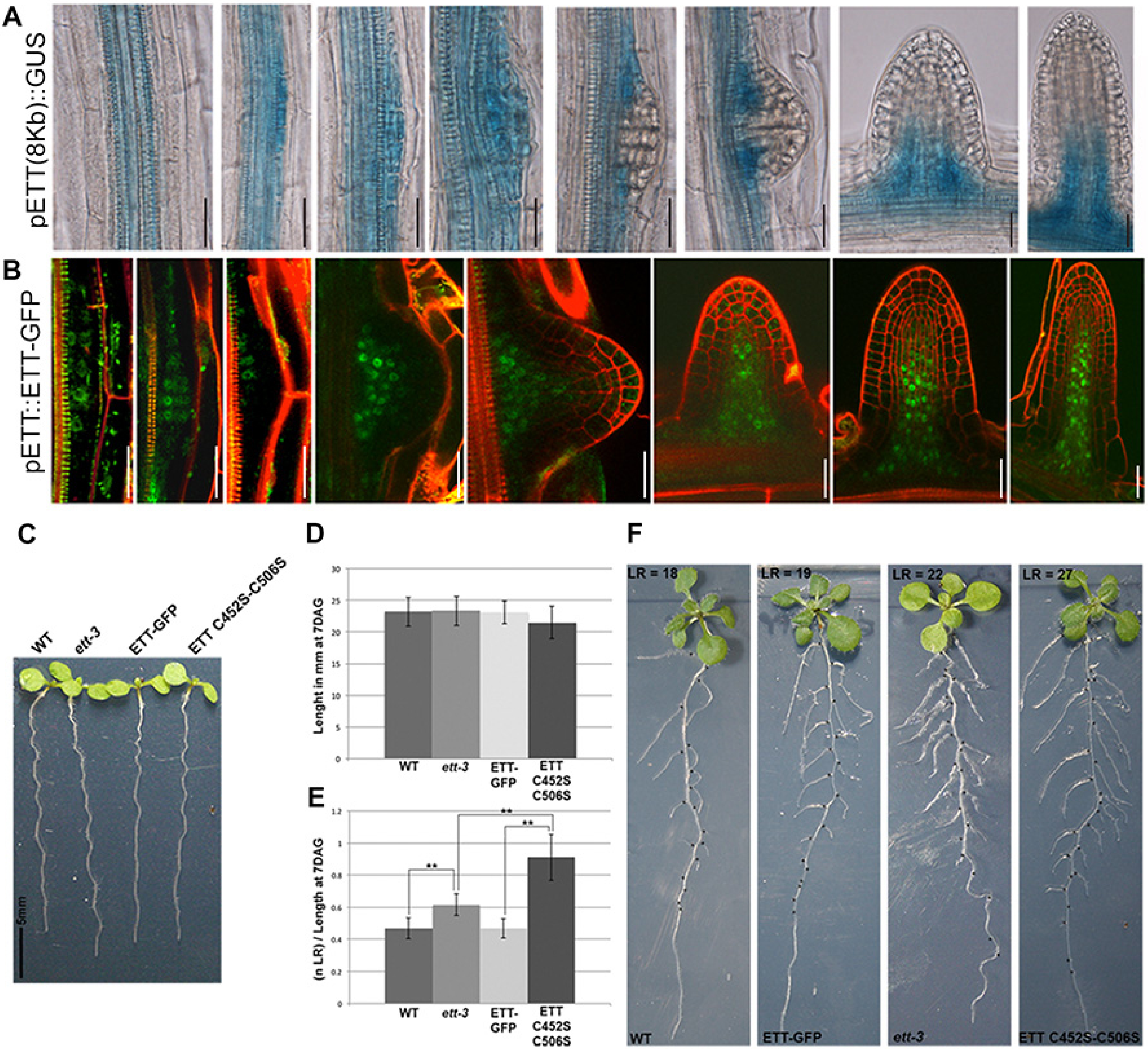

Supplemental Figure 8.

(A) GUS staining of *pETT(8Kb)::GUS* line during lateral root development; from left to right GUS expression was evident in the pericycle and in the emerging lateral root. Later in development the signal localised to the inner part of the lateral root, marking the vasculature.

(B) Confocal images of *pETT::ETT-GFP* line showing clear nuclear signal (green channel) in the emerging lateral root. Later in development the signal concentrate in the stele of the lateral root, in correspondence of the developing vasculature. Cell wall is stained with propidium iodide (red channel).

(C-D) Phenotypical images of seedlings (C) of wild-type, *ett-3*, *pETT::ETT-GFP* and *pETT::ETT-2C-S* line at 7 days after germination and length measurement (D) showing no statistical difference in growth among the 4 genotypes.

(E) Chart showing the ratio between lateral root number and total root length at 7 days after germination, ** is p value < 0.001.

(F) Phenotypical analyses of young plants of wild-type, *ett-3*, *pETT::ETT-GFP* and *pETT::ETT-2C-S*, each lateral root is marked by a black dot.

Scale bar (A-B): 20µM.

Supplemental Figure 9.

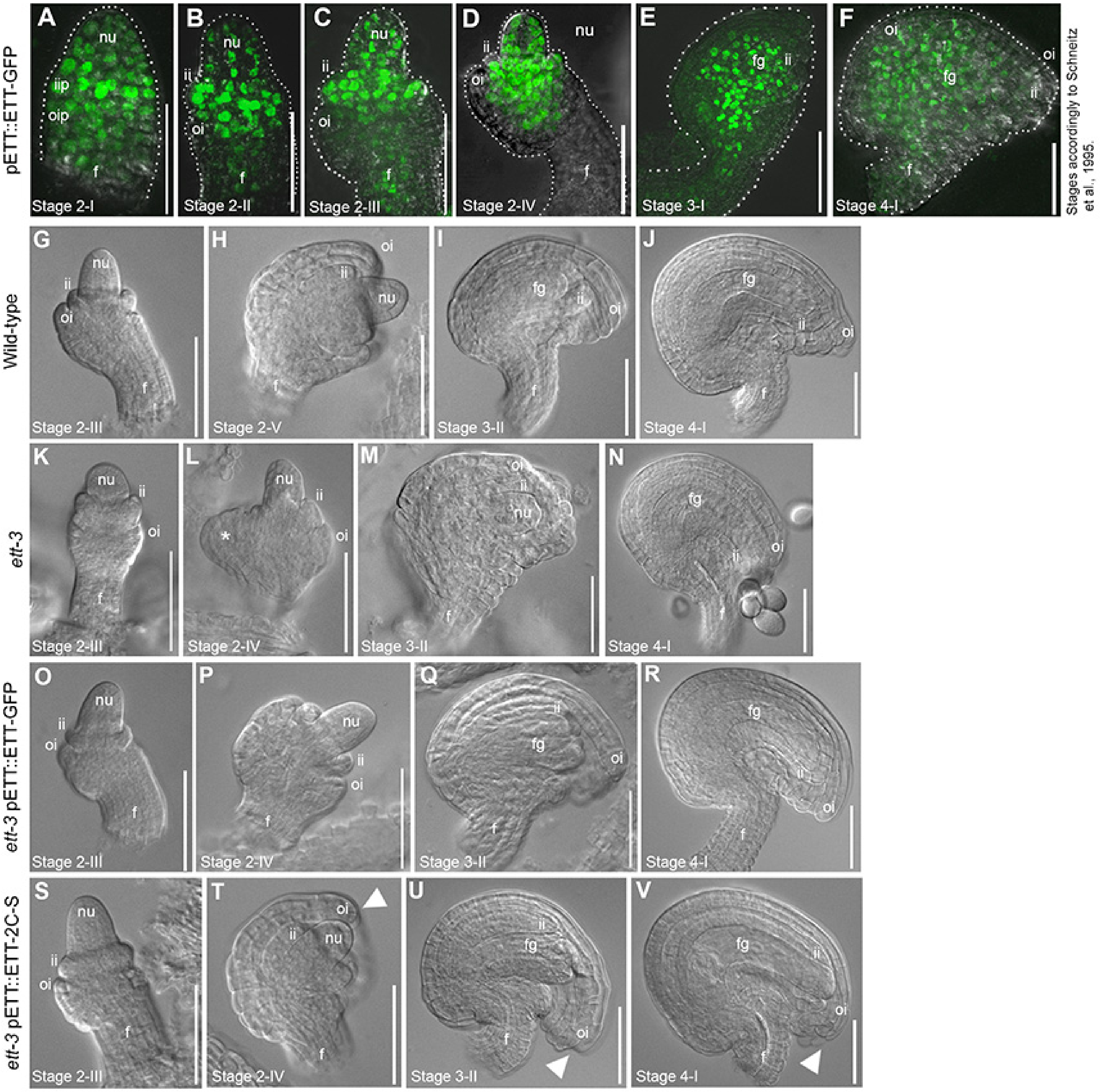

Supplemental Figure 9.

(A-F) Confocal images of *pETT::ETT-GFP* line during ovule development. At stage 2-I (A) GFP signal localised in the region from which inner and outer integuments will develop. Later in development, ETT-GFP localised predominantly in the inner integument and in the cells surrounding the developing female gametophyte. No expression in the female gametophyte nuclei can be detected.

(G-V) Phenotypical analyses of cleared ovules of wild-type (G-J), *ett-3* (K-N), *pETT::ETT-GFP* (O-R) and *pETT::ETT-2C-S* (S-V) lines from stage 2-III (G,K,O,P) to stage 4-I (J,N,R,V). Defects in outer integument growth are evident from stage 2-IV onward (I,M,Q,U) leading to an excessive overgrowth integuments (arrowhead) and deformed ovule. These abnormalities can potentially cause inefficient female gametophyte fertilisation.

fg: female gametophyte; nu: nucellus; ii: inner integument; oi: outer integument; f: funiculus.

Scale bar: 20µm.

Supplemental Figure 10.

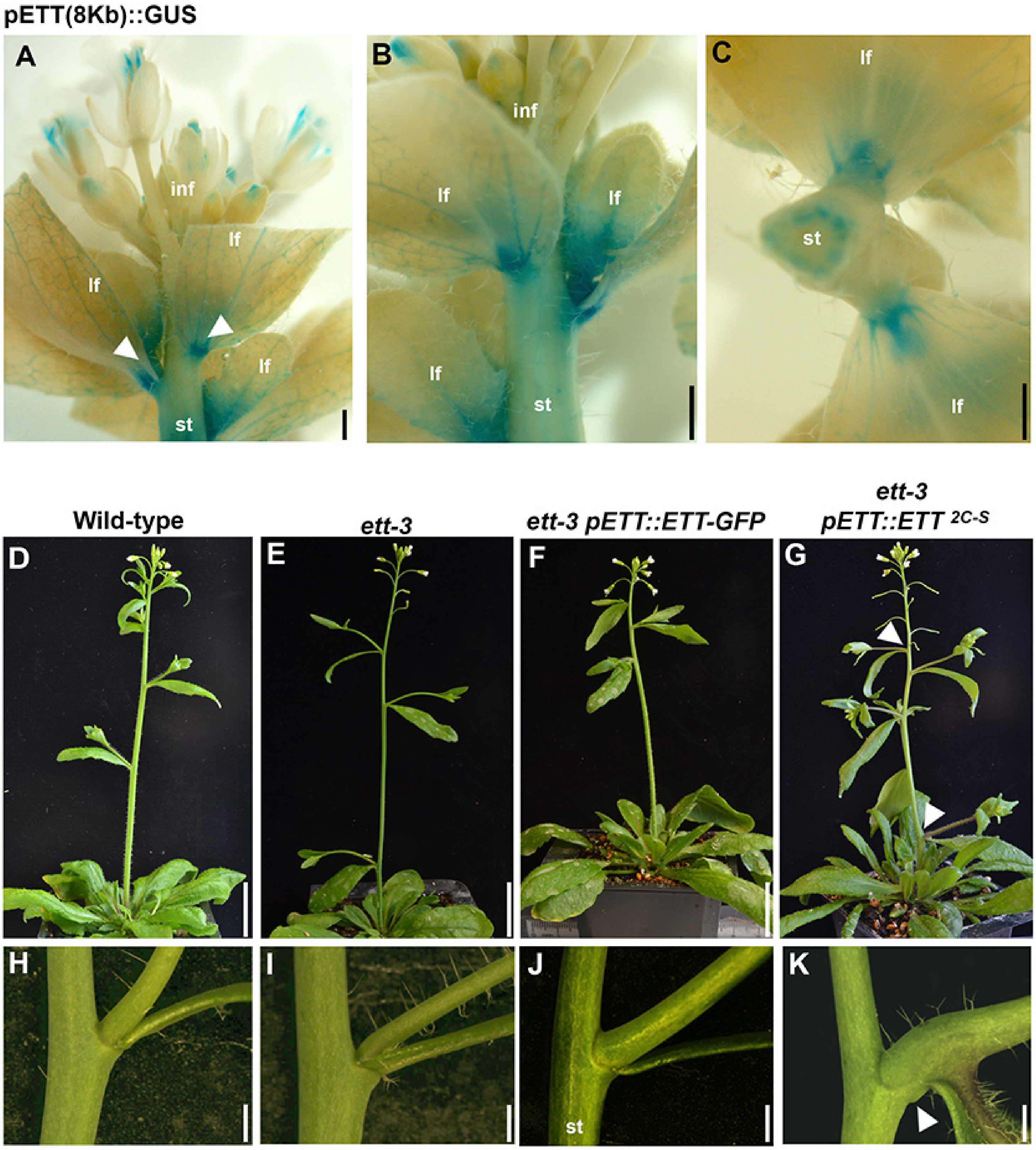

Supplemental Figure 10.
(A-C) GUS staining of *pETT(8Kb)::GUS* line in stem; strong blue signal was observed in correspondence of secondary branch emerge point. In particular GUS signal was mostly localised on the lower side of the branch (C) in correspondence of the point of conjunction of the branch with the stem. GUS signal was also evident in the leaf veins.
(D-G) Phenotypical analyses of wild-type, *ett-3*, *pETT::ETT-GFP* and *pETT::ETT-2C-S* plants. *pETT::ETT-2C-S* displayed higher number thick secondary branches with an angle of growth slightly pointing downward giving a more horizontal directional growth.
(H-K) Close-up images of secondary branch-stem internode. Deformities can be observed in the *pETT::ETT-2C-S* plants, which displayed a abnormal growth and distorted angle of growth of the branch.
inf: inflorescence; lf: leaf; st:stem.
Scale bar: (A-C; H-K) 5mm; (D-G) 1cm.

Supplemental Figure 11.

Putative partner		-W-L-H-A	-W-L-H-A 5mM 3AT	-W-L-H-A 10mM 3AT	IAA sensitivity
**AGL71**	At5g51870	Bright Green	Light Green	Light Green	N.A.
**BABY BOOM**	At5g17430	Bright Green	Bright Green	Light Green	YES
**BBX9**	At4g15250	Bright Green	Light Green	White	N.A.
**FIL**	At2g45190	Bright Green	Bright Green	Bright Green	WEAK above 100µM
**FLC**	At5g10140	Bright Green	Light Green	White	N.A.
**KNAT1**	At4g08150	Bright Green	Bright Green	Light Green	YES
**KNAT3**	At5g25220	Bright Green	Bright Green	Light Green	YES
**PRR3**	At5g60100	Bright Green	Light Green	Light Green	N.A.
**RPL**	At5g02030	Bright Green	Bright Green	Bright Green	YES
**TCP4**	At3g15030	Bright Green	Bright Green	Light Green	YES
**TCP18**	At3g18550	Bright Green	Bright Green	Bright Green	WEAK above 100µM
**Zinc finger protein**	At4g11680	Bright Green	Light Green	White	N.A.

Color code:

Bright Green: strong interaction

Light Green: weak interaction

White: no growth

N.A. = Not assessed

Supplemental Figure 11.
Summary table of the putative partners isolated through the yeast two-hybrid library screening of the REGIA transcription factor library with ETT-BD as prey. Each combination was on selective YSD media lacking W, L, A and H and supplemented with different concentration of 3-AT.

Supplemental table 2.

In situ	PID probe	Fw GATCCGACGGTCACATTATGC<br>Rev TCAAAAGTAATCGAACGCCGC
**In vivo co-localization**	pIND	Fw GGAAGACGGGGAGgaaagtgtgtaatccaaatcgttgcg<br>Rev GGAAGACGGCATTtttcatcttttcttatttctc
	IND	Fw GGAAGACGGAATGATGGAGCCTCAGCCTCACCATCTCC<br>Rev GGAAGACGGCGAAccGGGTTGGGAGTTGTGGTAATAAC
	YFP	Fw GGAAGACGGTTCG**ATGGTGAGCAAGGGCGAGGAGCTGTT**C<br>Rev GGAAGACGGAAGC**TTACTTGTACAGCTCGTCCATGC**
	pETT	Fw GGAAGACGGGGAGacaaattgtatcccaacccttactttc<br>Rev GGAAGACGGCATTtaaagagagagaaacagagataaag
	CFP	Fw GGAAGACGGTTCG**ATGGTGAGCAAGGGCGAGGAGCTGTT**C<br>Rev GGAAGACGGAAGC**TTACTTGTACAGCTCGTCCATGC**
**Error-Prone**	IND	Fw **AAGCAGTGGTATCAACGCAGAGTGGCCATTATGGCC**ATGATGGAGCCTCAGCCTCACCATC<br>Rev **TCTAGAGGCCGAGGCGGCCGACATG**TCAGGGTTGGGAGTTGTGGT
**IND D30G**	Y2H	Fw ggggacaagtttgtacaaaaaagcaggcttcATGATGGAGCCTCAGCCTCACC<br>Rev D→G mutation GATGGTGAGGACCATTGAGAAAAGC<br>Fw D→G mutation GCTTTTCTCAATGGTCCTCACCATC<br>Rev **GGGGACCACTTTGTACAAGAAAGCTGGGTG**TCAGGGTTGGGAGTTGTGGT
	Plant expression D30G	**Fw Ggtacc**ccttatgttaatatcaccgtaggc<br>Rev D→G mutation GATGGTGAGGACCATTGAGAAAAGC<br>Fw D→G mutation GCTTTTCTCAATGGTCCTCACCATC<br>Rev **ggatcc**TCAGGGTTGGGAGTTGTGGT
	Plant expression WT	Fw **Ggtacccccttatgttaatatcaccgtaggc**<br>Rev **ggatccTCAGGGTTGGGAGTTGTGGT**
**IND Ear Motif**	pIND	Fw GGAAGACGGGGAGgaaagtgtgtaatccaaatcgttgcg<br>Rev GGAAGACGGCATTtttcatcttttcttatttctc
	IND-EAR	Fw GGAAGACGGAATGATGGAGCCTCAGCCTCACCATCTCC<br>Rev GGAAGACGGAAGC**CTAAGCAAATCCAAGTCTAAGTTCAAGATCAAGATCAAG**GGGTTGGGAGT TGTGGTAATAAC
**Yeast-2-hybrid**	IND	Fw ccaacccgggcATGATGGAGCCTCAGCCTCACC<br>Rev ccaacccgggcATGATGGAGCCTCAGCCTCACC
	ETT	Fw ctagCCCGGGAATGGGTGGTTTAATCGATCTGAACG<br>Rev CTGTctgcagCTAGAGAGCAATGTCTAGCAACATG
	SPT	Refer to Girin et al. 2011
	ARF4	Fw ggggacaagtttgtacaaaaaagcaggcttcATGGAATTTGACTTGAATACTG<br>Rev GGGGACCACTTTGTACAAGAAAGCTGGGTGTCAAACCCTAGTGATTGTAGGAG
	ETT-ES	Fw TTAGcccgggaTCCAATTCAGGCAGCTTCGTAAC<br>Rev CTGTctgcagCTAGAGAGCAATGTCTAGCAACATG
	IND-IS	Fw ccaacccgggcATGATGGAGCCTCAGCCTCACC<br>Rev CCTGctgcagCTACATGTCTTCATCGTACTCTTC
	IND-(IS+HEC)	Fw ccaacccgggcATGATGGAGCCTCAGCCTCACC<br>Rev ATActgcagCTAACGGCGGTTCGGCTTAGGGAC
	IND(IS)-HEC3(HEC+bHLH)	Fw ccaacccgggcATGATGGAGCCTCAGCCTCACC<br>Extension Overlap Fw CGAAGAGTACGATGAAGACATGgtgctatgaaggaaatgatgtacaag<br>Extension Overlap Rev TTGTACATCATTTCCTTCATAGCACCATGTCTTCATCGTACTCTTCG<br>Rev ATCTctgcagCTAGATTAATTCTCCTACTCCTCTTC
	IND for pGADCg	Fw ggggacaagtttgtacaaaaaagcaggcttcATGATGGAGCCTCAGCCTCACC<br>Rev GGGGACCACTTTGTACAAGAAAGCTGGGTCGGGTTGGGAGTTGTGGTAAT
	ETT for pGBKCg	Fw ggggacaagtttgtacaaaaaagcaggcttcATGGGTGGTTTAATCGATCTG<br>Rev GGGGACCACTTTGTACAAGAAAGCTGGGTCGAGAGCAATGTCTAGCAACATG
**BiFC**	IND	Fw ggggacaagtttgtacaaaaaagcaggcttcATGATGGAGCCTCAGCCTCACC<br>Rev GGGGACCACTTTGTACAAGAAAGCTGGGTGTCAGGGTTGGGAGTTGTGGT
	ETT	Fw ggggacaagtttgtacaaaaaagcaggcttcATGGGTGGTTTAATCGATCTG<br>Rev GGGGACCACTTTGTACAAGAAAGCTGGGTGCTAGAGAGCAATGTCTAGCA
**pETT::ETT-GFP**	pETT::ETT	Fw AAGCTTgagcaatcctatacggagttc<br>Rev AAGCTTGAGAGCAATGTCTAGCAACATG
**ChIP**	Region 7	Fw caagcattagaacatagccagg<br>Rev gtttggtttatacttcaaagac
	Region 6	Fw ggtttttgtgaccaatacac<br>Rev ccaattattatttctccctc
	Region 5	Fw gtctttaggaaccaacgagg<br>Rev gtggaagagggacaaagtcg
	Region 4	Fw gagtacattatatgaatacg<br>Rev ttatcttttccatgagttgc
	Region 3	Fw cctttagaaaattgatgttatc<br>Rev ggagaaatattggactagtgattc
	Region 2	Fw gaatcactagtccaatatttctcc<br>Rev cagtaaaaagacgttacagagacc
	Region 1	Fw ggtctctgtaacgtctttttactg<br>Rev gatgaaacaggacagggtgtc
	MLU (housekeeping gene)	See Schiessl et al., 2014
**Yeast One Hybrid**	PID Region2 wt	Fw CCCGGGGAGCTCcaggcacgtgacaacgtctc<br>Rev TCTAGACCGCGGacttttcaacgcgtgagagagg
	PID Region 2 Mut	Fw CCCGGGGAGCTCcaggcacgccccaacgtctcacacgtggggtatatcttacg<br>Rev TCTAGACCGCGGacttttcaacgcgtgagagagg
