# Supplementary material for: A noncanonical auxin-sensing mechanism is required for organ morphogenesis in Arabidopsis
Source: Genes Dev. 2016 Oct 15;30(20):2286–96. doi: 10.1101/gad.285361.116 (PMC5110995; doi:10.1101/gad.285361.116)
Supplement: Supplemental Material [file supp_30.20.2286_Supplemental_Table_S1.pdf]

Transcript (	DEX Bi-weig	DMSO Bi-w	Fold Chang	ANOVA p-va	FDR p-value	Gene Symb	Description	GO Biologic
13525497	8.33	4.57	13.58	0.001342	0.130717	SAG29	senescence	leaf senesce
13398316	6.07	3.77	4.92	0.000028	0.091234	AT2G28570	hypothetica	biological_p
13417323	6.2	3.93	4.85	0.000385	0.102587	AT2G35640	homeodom	regulation c
13347125	6.88	4.61	4.82	0.000097	0.091234	LOL1	lsd one like	defense res
13368493	7.21	4.97	4.71	0.000419	0.104959	GA3OX1	gibberellin :	response tc
13443516	7.18	4.96	4.67	0.038386	0.359121	SRG3	glyceropho:	glycerol me
13514247	7	4.83	4.51	0.019049	0.287235	AT5G53048		
13484339	9.35	7.24	4.29	0.000048	0.091234	XTH15	probable xy	carbohydra
13537364	5.86	3.84	4.03	0.006051	0.192523	MYB111	myb domai	regulation c
13414201	8.25	6.36	3.7	0.008467	0.215497	AT2G28510	Dof zinc fin	regulation c
13536593	7.18	5.3	3.67	0.000409	0.104959	AT5G47550	cysteine pr	defense res
13517975	6.73	5.02	3.26	0.000031	0.091234	AT5G61610	oleosin	lipid storag
13503882	7.21	5.54	3.17	0.018329	0.284042	SPX1	SPX domair	phosphate i
13494535	6.59	4.94	3.13	0.001674	0.133855	CP1	cysteine pr	proteolysis;
13417922	7.27	5.66	3.06	0.000035	0.091234	XTH32	probable xy	cell wall ma
13497172	5.8	4.19	3.05	0.002583	0.149762	HB51	homeobox	regulation c
13442302	6.66	5.09	2.98	0.000336	0.102587	AT3G61490	polygalactu	carbohydra
13448869	7.61	6.05	2.95	0.000623	0.114633	AT3G13310	chaperone	protein folc
13363825	4.89	3.36	2.88	0.001212	0.126383	chr31	chromatin remodeling 3	
13364229	5.09	3.58	2.85	0.009497	0.226597	GPAT1	sn-glycerol-	phosphatid
13421812	6.82	5.31	2.84	0.002693	0.152554	TRM13	phosphatid	response tc
13366223	6.17	4.66	2.84	0.000683	0.114662	XTH33	probable xy	carbohydra
13511543	7.26	5.76	2.83	0.000308	0.102237	AT5G47050	S-ribonuclease binding	
13459098	7.19	5.71	2.78	0.00049	0.105453	PUB22	E3 ubiquitir	respiratory
13410207	5.45	3.99	2.75	0.001668	0.133855	AT2G18969	hypothetica	biological_p
13526725	7.18	5.75	2.69	0.000598	0.113991	PCME	prenylcyste	metabolic p
13340027	6.78	5.36	2.68	0.00009	0.091234	FLP	FOUR LIPS t	regulation c
13535298	9.07	7.67	2.63	0.002743	0.153969	TZF5	zinc finger (	regulation c
13458752	8.93	7.54	2.61	0.002029	0.141625	AT3G51660	Tautomeras	tryptophan
13448584	4.21	2.85	2.56	0.025757	0.317867	MYB10	myb domai	regulation c
13499397	7.01	5.66	2.55	0.000234	0.09768	FLS1	flavonol syr	response tc
13334311	4.32	2.97	2.55	0.009168	0.223306	ETC1	enhancer o	cell fate spe
13536511	8.27	6.92	2.55	0.000064	0.091234	HAT2	homeobox-	transcriptio
13472434	6.62	5.28	2.53	0.000054	0.091234	AT4G23500	putative po	carbohydra
13497186	4.75	3.43	2.51	0.013608	0.258609	AT5G03890	hypothetical protein	
13422352	8.51	7.19	2.5	0.007962	0.210916	BOR1	boron trans	transition m
13490390	5.56	4.25	2.48	0.000056	0.091234	AT4G27480	core-2/I-bra	carbohydra
13337474	5.25	3.96	2.45	0.010524	0.237798	AT1G08590	CLV1-like le	response tc
13497047	5.49	4.2	2.45	0.045872	0.378298	AT4	hypothetica	cellular resp
13403947	9.31	8.03	2.44	0.000455	0.105453	ZFP8	zinc finger p	MAPK casca
13472239	5.65	4.37	2.43	0.005778	0.190519	AT4G23000	calcineurin-	cellular resp
13529177	7.99	6.72	2.42	0.00063	0.114633	AT5G22020	strictosidin	biosyntheti
13535891	6.09	4.83	2.39	0.001572	0.133855	AT5G45650	subtilase fa	proteolysis;
13473882	7.02	5.77	2.37	0.004295	0.173912	FBA5	fructose-bis	maltose me
13499587	9.32	8.1	2.33	0.001364	0.130717	EXL4	protein exo	defense res
13503695	6.02	4.81	2.32	0.000089	0.091234	AT5G19740	peptidase N	proteolysis
13344418	6.22	5.03	2.28	0.00007	0.091234	IDD16	shoot gravit	regulation c

13477800	5.52	4.34	2.28	0.00008	0.091234	AT4G35070	SBP (S-ribo	biological_p
13340153	6.53	5.35	2.27	0.005486	0.18691	PAP3	purple acid	phosphatas
13461411	4.27	3.09	2.27	0.010349	0.234777	AT3G58060	putative me	cation trans
13416597	5.54	4.37	2.25	0.001636	0.133855	AT2G34020	calcium-bin	cytokinin m
13465230	6.11	4.95	2.23	0.007055	0.200857	AT4G03140	NAD(P)-bin	metabolic p
13470543	4.04	2.88	2.23	0.002178	0.141724	OFP5	ovate famil	embryo sac
13492019	8.27	7.12	2.22	0.001472	0.133855	CSLC5	cellulose-sy	cell wall bio
13487729	6.45	5.3	2.21	0.039425	0.362148	MSRB7	peptide me	response to
13380044	5.12	3.99	2.2	0.006709	0.197793	AT1G55760	BTB/POZ do	biological_p
13343207	8.1	6.97	2.2	0.000217	0.096878	PIP5K1	phosphatid	phosphatid
13504496	9.07	7.95	2.18	0.002654	0.152554	FAR1	fatty acyl-C	microsporo
13518605	5.13	4.01	2.17	0.023253	0.308453	AT5G62960	hypothetica	biological_p
13340529	4.81	3.7	2.16	0.000379	0.102587	AT1G15760	Sterile alph	biological_p
13486663	5.84	4.73	2.16	0.003784	0.167951	AT4G18970	GDSL estera	lipid metab
13385599	4.11	3.01	2.14	0.013312	0.256913	AT1G70080	terpenoid s	metabolic p
13540544	4.41	3.32	2.13	0.028932	0.324813	XTH12	probable xy	carbohydra
13351947	5.86	4.77	2.13	0.000093	0.091234	AT1G54120	hypothetica	biological_p
13449710	3.86	2.79	2.11	0.000814	0.120571	SPL5	squamosa p	regulation o
13494916	9.83	8.75	2.11	0.000162	0.096878	PCK1	phosphoen	gluconeoge
13406417	4.52	3.45	2.1	0.025316	0.316475	AT2G47780	Rubber elo	fatty acid ca
13519141	8.54	7.47	2.1	0.000659	0.114662	EXL2	protein EXO	response to
13384742	4.9	3.83	2.09	0.004408	0.174463	AT1G67910	hypothetica	biological_p
13431958	6.29	5.23	2.08	0.003602	0.166345	AT3G22810	hypothetica	signal trans
13411104	5.68	4.62	2.07	0.002043	0.141724	AT2G20835	hypothetica	biological_p
13460611	7.52	6.47	2.07	0.001652	0.133855	AT3G56060	Glucose-me	alcohol met
13539026	8.56	7.51	2.07	0.001	0.1227	PMEPCRF	putative pe	protein targ
13389141	5.39	4.34	2.06	0.006561	0.195757	AT1G78860	curculin-like (mannose-	
13474655	6.29	5.25	2.06	0.025505	0.316854	AT4G28410	tyrosine tra	cellular ami
13476930	9.27	8.22	2.06	0.04291	0.370821	SQD1	sulfoquinov	glycolipid b
13445203	9.26	8.22	2.06	0.000558	0.111612	RCI2A	Hydrophob	glycolysis; v
13500157	7.26	6.22	2.06	0.00522	0.184185	KNAT4	homeobox	regulation o
13520416	3.73	2.69	2.06	0.022762	0.306183	MAPKKK19	mitogen-ac	protein pho
13364059	7.99	6.96	2.04	0.002412	0.146331	AT1G06000	UDP-glycos	metabolic p
13437976	7.18	6.15	2.04	0.000844	0.122048	F3H	flavanone 3	anthocyanin
13463715	4.4	3.37	2.04	0.012867	0.254065	AT3G63450	RNA-bindin	biological_p
13335980	5.21	4.19	2.03	0.000059	0.091234	PFA-DSP1	atypical dua	intracellular
13479216	4.62	3.6	2.03	0.024278	0.313287	PLC1	putative ph	lipid metab
13400712	7.14	6.12	2.02	0.02592	0.318512	AT2G34620	mitochondr	biological_p
13380874	8.14	7.13	2.01	0.00022	0.096878	ZW9	TRAF-like p	biological_p
13396150	5.47	4.46	2.01	0.005245	0.184607	SCPL13	serine carb	proteolysis
13455873	6.32	5.32	2.01	0.000636	0.114633	AT3G43960	putative cys	proteolysis;
13505451	5.65	4.65	2.01	0.02325	0.308453	AT5G24880	hypothetica	biological_p
13459251	7.54	6.54	2	0.002763	0.153969	AT3G52870	IQ calmodu	biological_p
13506365	3.9	2.9	2	0.003453	0.163117	AT5G27300	pentatricop	biological_p
13403955	5.39	4.4	1.98	0.000233	0.09768	AT2G41990	hypothetica	biological_p
13539278	3.19	2.21	1.97	0.000468	0.105453	PORA	protochloro	metabolic p
13468952	3.73	2.75	1.97	0.006081	0.192839	ABCG43	ABC transp	ATP catabo
13411796	5	4.03	1.97	0.003354	0.161359	ACS4	1-aminocyc	biosyntheti

13390930	4.04	3.07	1.96	0.026023	0.319235	AT2G02630	cysteine/his	oxidation-re
13466860	5.34	4.37	1.96	0.009526	0.226911	TT8	transcriptio	anthocyanii
13534610	8.21	7.24	1.96	0.003712	0.167043	AOS	allene oxide	sulfur amin
13511549	6.34	5.38	1.95	0.000434	0.105453	AT5G47060	hypothetica	biological_p
13521035	5.87	4.91	1.95	0.00183	0.136553	AT5G01790	hypothetica	cell wall bio
13488642	3.42	2.46	1.95	0.022407	0.303829	AT4G23580	F-box/kelch	biological_p
13349877	4.27	3.32	1.94	0.014458	0.263617	AT1G49220	RING-H2 fir	protein ubi
13345782	4.7	3.77	1.91	0.001178	0.125727	AT1G29270	hypothetica	biological_p
13431986	6.1	5.17	1.91	0.004323	0.173912	ath-MIR167a		
13464350	4.32	3.39	1.91	0.001728	0.134833	AT4G01335	hypothetica	biological_p
13540675	7.62	6.69	1.91	0.042859	0.370821	AT5G57785	hypothetica	response to
13453302	5	4.06	1.91	0.002933	0.156582	AT3G24450	heavy meta	copper ion
13474562	4.89	3.97	1.9	0.002478	0.147551	ULT1	protein ULT	meristem d
13442489	4.99	4.06	1.9	0.026872	0.321413	NGA2	B3 domain-	regulation c
13407656	2.3	1.38	1.9	0.018601	0.285075	AT2G04070	MATE efflu	transport; c
13540856	5.33	4.41	1.9	0.000659	0.114662	MES18	methyl este	protein pho
13402294	5.45	4.53	1.89	0.000473	0.105453	TBL34	trichome bi	glucuronox
13468376	4.02	3.1	1.89	0.034166	0.343907	DMT2	DNA methy	DNA methy
13491005	3.91	2.99	1.89	0.014277	0.262593	OZF2	zinc finger (	regulation c
13361869	7.53	6.61	1.89	0.012315	0.251206	KCS1	3-ketoacyl-(	very long-cl
13387816	9.63	8.72	1.88	0.00035	0.102587	GASA1	GA-respons	polysacchar
13460579	9	8.09	1.88	0.018523	0.284647	UGP3	UDP-glucos	metabolic p
13490624	5.33	4.42	1.88	0.000217	0.096878	SLAH2	SLAC1 hom	transmemb
13460831	5.03	4.12	1.87	0.002998	0.157821	AT3G56620	nodulin MtN21-like trai	
13448607	8.72	7.82	1.87	0.001071	0.124366	AT3G12920	S-ribonucle	regulation c
13520323	4.26	3.36	1.87	0.000811	0.120571	AT5G66800	hypothetica	biological_p
13529414	5.84	4.94	1.86	0.040312	0.364836	AT5G22460	esterase/lip	biological_p
13414913	8.09	7.2	1.85	0.003056	0.158608	TCL2	protein tricl	negative re
13453001	4.54	3.65	1.85	0.01571	0.270184	AT3G23880	F-box/kelch	biological_p
13466519	6.37	5.49	1.85	0.036044	0.349762	EXO	phosphate-	response tc
13501251	9.97	9.08	1.85	0.011129	0.241534	TT4	chalcone sy	response tc
13542051	6.36	5.47	1.85	0.020659	0.294957	PIF7	transcriptio	regulation c
13355724	9.12	8.24	1.84	0.003265	0.159634	AT1G66180	aspartyl prc	proteolysis;
13381336	4.44	3.56	1.83	0.016617	0.274169	GolS7	galactinol s	carbohydra
13497797	8.33	7.46	1.83	0.00163	0.133855	CHIL	Chalcone-fl	anthocyanii
13517088	4.05	3.18	1.83	0.007773	0.208034	BOA	protein BRC	regulation c
13434126	2.56	1.69	1.82	0.022447	0.304223	AT3G27980	plant invert	cell wall mc
13460624	5.6	4.74	1.82	0.000267	0.101835	AT3G56080	probable m	methylatior
13380490	4.15	3.29	1.82	0.000786	0.118831	PAP1	transcriptio	regulation c
13478556	6.34	5.48	1.82	0.002063	0.141724	SPT	transcriptio	regulation c
13404737	6.1	5.25	1.81	0.015223	0.26738	KMD3	F-box/kelch	biological_p
13405911	4.22	3.38	1.8	0.027248	0.321761	ath-MIR166a		
13422114	4.81	3.96	1.8	0.001826	0.136553	CYP78A6	cytochrome	seed develc
13468282	2.55	1.7	1.8	0.022063	0.302403	AT4G13760	glycoside hy	carbohydra
13490738	4.12	3.27	1.8	0.000175	0.096878	AT4G28180	hypothetical protein	
13531958	3.45	2.59	1.8	0.022143	0.302403	AT5G29560	caleosin-rel	biological_p
13373372	5.33	4.49	1.79	0.01233	0.251206	AT1G28570	GDSL estera	protein targ
13398746	4.51	3.67	1.79	0.002905	0.156582	UGT71C2	UDP-glucos	metabolic p

13524690	7.13	6.29	1.79	0.001152	0.125727	AT5G11070	hypotheticą	response to
13478776	4.19	3.35	1.79	0.010151	0.232869	CYP91A2	cytochrome	indole gluc
13373159	6.65	5.82	1.78	0.004281	0.173912	SCPL45	serine carb	proteolysis
13438560	8.81	7.97	1.78	0.00588	0.191511	AT3G52470	late embryo	biological_p
13474439	6.7	5.88	1.77	0.0009	0.122048	AT4G27900	CCT motif f	biological_p
13478807	7.28	6.45	1.77	0.002516	0.148454	AT4G37530	peroxidase	response to
13496832	7.18	6.35	1.77	0.002338	0.144358	AT5G03120	hypotheticą	biological_p
13348089	6.75	5.93	1.76	0.008883	0.219081	AT1G35516	myb-like tra	response to
13385529	6.14	5.33	1.76	0.003407	0.162295	AT1G69890	hypotheticą	response to
13464816	2.22	1.4	1.76	0.046962	0.381558	AGL51	protein aga	regulation c
13341424	7.17	6.36	1.75	0.002854	0.155449	AT1G17500	ATPase E1-I	cation trans
13374554	4.59	3.78	1.75	0.002072	0.141724	AT1G31550	GDSL estera	lipid metab
13414633	6.39	5.58	1.75	0.005495	0.186976	CTF2B	CTF2B like c	metabolic p
13448929	5.38	4.57	1.75	0.008412	0.215208	AT3G13403	defensin-lik	defense res
13495546	3.05	2.25	1.75	0.013446	0.257733	GH9B16	glycosyl hyc	carbohydra
13360701	9.57	8.76	1.75	0.000729	0.117031	RHM1	UDP-L-rhan	nucleotide-
13494559	6.26	5.45	1.75	0.028098	0.323325	AT4G36945	PLC-like pho	lipid metab
13379824	4.72	3.92	1.74	0.047316	0.382626	AT1G55380	cysteine/his	oxidation-re
13473509	4.72	3.92	1.74	0.000497	0.106093	AT4G25780	putative pa	biological_p
13475608	7.28	6.48	1.74	0.012906	0.254065	BRS1	serine carb	proteolysis;
13486197	5.72	4.91	1.74	0.001505	0.133855	AT4G17680	SBP (S-ribor	regulation c
13361957	4.43	3.65	1.73	0.026799	0.321413	AT1G01390	UDP-glycos	metabolic p
13378161	3.89	3.1	1.73	0.003458	0.163117	AT1G51330	serine prote	negative re
13396472	6.62	5.83	1.73	0.025569	0.316883	AT2G23910	Rossmann-f	response to
13418828	7.33	6.54	1.73	0.001514	0.133855	AT2G39130	transmembrane amino	
13540085	6.6	5.81	1.73	0.031114	0.334708	OXS3	oxidative st	response to
13358484	4.06	3.27	1.73	0.049767	0.389098	AT1G72890	TIR-NBS cla	autophagy;
13412046	5.34	4.55	1.72	0.020755	0.295178	MES8	methyl esterase 8	
13441859	4.59	3.81	1.72	0.028354	0.323767	AT3G60647	hypothetical protein	
13475498	3.01	2.24	1.72	0.00299	0.157821	AT4G30180	hypotheticą	regulation c
13351374	4.93	4.16	1.71	0.000667	0.114662	AT1G52800	oxidoreduc	oxidation-re
13371740	3.58	2.8	1.71	0.01124	0.242295	AT1G23640	hypothetical protein	
13483911	4.49	3.71	1.71	0.003359	0.161359	YUC2	protein YUC	auxin biosy
13349869	6.76	6	1.7	0.043502	0.372944	AT1G49200	RING-H2 fir	protein ubi
13356611	4.65	3.89	1.7	0.015124	0.266435	AT1G68500	hypothetical protein	
13365497	5.33	4.57	1.7	0.034195	0.343907	AT1G09157	hypothetical protein	
13396165	8.53	7.76	1.7	0.000327	0.102587	SNG1	serine carb	sulfur amin
13434456	4.76	3.99	1.7	0.039029	0.360211	AT3G28899	hypothetical protein	
13388733	7.19	6.43	1.7	0.006338	0.194525	LAX3	auxin influx	polysacchar
13535130	5.57	4.81	1.7	0.014994	0.265798	YUC5	probable in	response to
13403837	7.36	6.6	1.69	0.006719	0.197793	GPDHC1	glycerol-3-p	carbohydra
13405999	6.39	5.64	1.69	0.002308	0.144328	NGA1	B3 domain-	regulation c
13414908	2.9	2.15	1.69	0.000993	0.122396	ETC2	protein ENH	regulation c
13544723	6.68	5.93	1.69	0.000925	0.122048	NPY3	MAB4/ENP,	response to
13360951	9.14	8.39	1.69	0.000891	0.122048	AT1G79110	zinc ion bin	regulation c
13344610	3.68	2.93	1.68	0.003287	0.160033	AT1G26290	hypotheticą	biological_p
13366784	4.47	3.73	1.68	0.010578	0.237798	AT1G11740	ankyrin rep	biological_p
13438558	6.18	5.43	1.68	0.005359	0.185648	AT3G52460	hydroxyproline-rich glyc	

13488707	4.94	4.19	1.68	0.049167	0.388085	AT4G23770	hypotheticɛ	biological_p	
13502952	4.96	4.21	1.68	0.002436	0.146604	NAC088	NAC domai	regulation c	
13488719	8.36	7.61	1.68	0.005285	0.18501	AT4G23820	glycoside h	carbohydra	
13361487	8.13	7.38	1.68	0.005315	0.185319	NTT1	ADP,ATP ca	transport	
13385338	6.32	5.58	1.67	0.02109	0.29621	AGO7	protein arg	vegetative p	
13418625	4.34	3.6	1.67	0.037317	0.354989	AT2G38640	hypotheticɛ	biological_p	
13334797	7.97	7.23	1.67	0.004194	0.173762	CER1	protein ECE	response tc	
13347035	2.51	1.78	1.66	0.01959	0.289527	AT1G32280	bifunctiona	lipid transp	
13383168	6.53	5.8	1.66	0.001661	0.133855	BDG1	putative alp	response to	
13385025	6.97	6.25	1.66	0.043581	0.373063	PHO1;H1	phosphate	phosphate i	
13507587	6.54	5.81	1.66	0.010061	0.232094	CAT3	cationic am	amino acid	
13341993	3.76	3.04	1.66	0.000208	0.096878	ICS2	Isochorisma	biosyntheti	
13366741	6.66	5.93	1.66	0.015045	0.265987	XTH8	probable xy	carbohydra	
13530775	6.77	6.04	1.66	0.016774	0.275056	ESE3	ethylene-re	regulation c	
13373233	6.3	5.57	1.65	0.000342	0.102587	AT1G28260	Telomerase	biological_p	
13375517	5.08	4.35	1.65	0.013065	0.254419	AT1G34640	peptidase;	signal pepti	
13408579	6.95	6.22	1.65	0.03308	0.340483	AT2G12462	hypotheticɛ	biological_p	
13445219	7.85	7.13	1.65	0.00036	0.102587	AT3G05910	pectinacety	cell wall bic	
13449897	4.22	3.5	1.65	0.036037	0.349762	AT3G15700	P-loop cont	defense res	
13473843	3.54	2.82	1.65	0.049352	0.388085	AT4G26460	S-adenosyl-	methylatior	
13479278	6.54	5.82	1.65	0.000761	0.118831	MYB4	transcriptio	polyamine c	
13523164	3.35	2.62	1.65	0.04028	0.364836	AT5G07571	Oleosin fam	biological_p	
13357325	5.96	5.23	1.65	0.007423	0.20481	CYCD1;1	cyclin-D1-1	mitotic cell	
13414696	9.93	9.2	1.65	0.000083	0.091234	FAD3	omega-3 fa	lipid metab	
13501345	5.5	4.79	1.65	0.02675	0.321413	AT5G14090	hypotheticɛ	biological_p	
13409265	8.43	7.72	1.64	0.000386	0.102587	PAP10	purple acid	cellular resp	
13498092	4.42	3.71	1.64	0.037411	0.355256	WOX7	WUSCHEL-r	regulation c	
13384484	5.05	4.33	1.64	0.003884	0.169375	AT1G67360	Rubber elor	biological_p	
13477262	6.56	5.85	1.64	0.003562	0.165078	ABF3	abscisic aci	response tc	
13521364	5.16	4.45	1.64	0.000607	0.114633	AT5G02890	HXXXD-type	acyl-transf	
13381189	5.67	4.96	1.63	0.010326	0.234777	AGL49	protein aga	transcriptio	
13395627	4.61	3.91	1.63	0.016527	0.273884	CESA9	cellulose sy	plant-type c	
13524333	6.95	6.25	1.63	0.037242	0.354672	H3.1	histone H3	nucleosome	
13385383	2.58	1.87	1.63	0.034568	0.344869	AGL94	protein aga	transcriptio	
13514338	6.63	5.92	1.63	0.02375	0.311779	AT5G53144	hypothetical protein		
13347920	2.79	2.1	1.62	0.040738	0.365457	HDG10	homeodom	regulation c	
13450736	6.82	6.13	1.62	0.011126	0.241534	WOX1	WUSCHEL-r	regulation c	
13458732	9.19	8.49	1.62	0.005986	0.19182	LTP5	pathogenes	very long-cl	
13511613	3.52	2.83	1.62	0.013907	0.259998	ADR1-L3	protein ADI	defense res	
13534297	2.25	1.56	1.62	0.034949	0.345677	ath-MIR319b			
13541905	5.91	5.22	1.62	0.045861	0.378298	TCP5	transcriptio	MAPK casca	
13462836	9.82	9.13	1.62	0.001054	0.123978	BRH1	brassinoste	response tc	
13500329	7.7	7.01	1.62	0.0218	0.300455	AT5G11420	hypotheticɛ	biological_p	
13357705	5.85	5.16	1.62	0.004454	0.174646	AT1G71110	hypotheticɛ	biological_p	
13390224	6.14	5.44	1.62	0.028385	0.323767	NIP6;1	aquaporin I	transport; r	
13409394	5.85	5.16	1.62	0.013828	0.25972	MYB7	myb domai	regulation c	
13419256	5.95	5.25	1.62	0.012334	0.251206	HSPRO2	HS1 PRO-1	response tc	
13374042	5.24	4.55	1.61	0.022271	0.302987	AT1G30280	chaperone DnaJ-domai		

13383344	5.31	4.63	1.61	0.023195	0.308222	4CL3	4-coumarat	metabolic p
13487347	7.37	6.69	1.61	0.000099	0.091234	FAH2	fatty acid h	fatty acid bi
13534800	3	2.31	1.61	0.019496	0.288735	AT5G43030	cysteine/his	intracellular
13364238	8.27	7.59	1.61	0.001524	0.133855	AT1G06550	3-hydroxyis	fatty acid be
13447894	2.67	1.99	1.61	0.003848	0.168472	AT3G11580	AP2/B3-like	regulation c
13356210	4.92	4.25	1.6	0.019104	0.287378	AT1G67570	hypothetica	sister chron
13510740	5.91	5.23	1.6	0.016899	0.276034	AT5G45100	BOI-related	regulation c
13513354	4.46	3.78	1.6	0.00427	0.173912	APC2	mitochondr	transport; A
13514542	3.15	2.47	1.6	0.004497	0.175609	OPT8	oligopeptid	oligopeptid
13375015	7.74	7.06	1.6	0.033533	0.341868	GBSS1	granule-bou	response to
13434848	4.28	3.61	1.6	0.023465	0.309786	AT3G32150	hypothetica	biological_p
13338203	6.77	6.11	1.59	0.020583	0.294957	BCAT-1	branched-c	metabolic p
13346361	3.19	2.52	1.59	0.000091	0.091234	UGT78D1	UDP-glucos	metabolic p
13357635	7.72	7.06	1.59	0.000031	0.091234	AT1G70900	hypothetical protein	
13383648	2.66	1.99	1.59	0.044908	0.375333	CYP702A1	cytochrome	tryptophan
13399496	5.17	4.51	1.59	0.048534	0.386889	AT2G31850	hypothetica	biological_p
13419766	5.3	4.63	1.59	0.001422	0.132001	SSL2	strictosidin	biosyntheti
13441679	4.56	3.89	1.59	0.023526	0.309983	AT3G60290	oxidoreduc	biosyntheti
13512993	3.54	2.87	1.59	0.000982	0.122048	AT5G50140	ankyrin rep	biological_p
13517163	8.46	7.79	1.59	0.024675	0.314249	AT5G59690	histone H4	nucleosome
13405857	5.03	4.36	1.59	0.000414	0.104959	ARF11	auxin respo	regulation c
13488286	6.61	5.93	1.59	0.000196	0.096878	AT4G22990	SPX domain	transmemb
13437014	5.55	4.88	1.59	0.012526	0.252577	AT3G48460	GDSL estera	lipid metab
13516311	8.79	8.12	1.59	0.001491	0.133855	CER3	protein ECE	very long-ch
13450177	5.86	5.2	1.58	0.000967	0.122048	PAP1	phytochron	regulation c
13480193	2.37	1.71	1.58	0.046505	0.380112	ADF8	actin depol	trichoblast
13487357	6.75	6.08	1.58	0.005907	0.191511	AT4G20880	ethylene-responsive/re	
13521980	5.68	5.03	1.58	0.00033	0.102587	KCS19	3-ketoacyl-(	fatty acid bi
13411803	6.35	5.69	1.58	0.020601	0.294957	SQE2	squalene ep	metabolic p
13521293	5.43	4.77	1.58	0.008365	0.214904	AT5G02570	histone H2B	nucleosome
13387050	3.38	2.72	1.58	0.044841	0.375333	AT1G73890	protease in	lipid transp
13441861	4.93	4.27	1.58	0.043678	0.373063	AT3G60650	hypothetica	lateral root
13338024	2.67	2.02	1.57	0.018953	0.287235	MYB61	myb domai	vasculature
13375703	1.86	1.22	1.57	0.029638	0.327695	AT1G35537	defensin-lik	defense res
13406379	7.01	6.36	1.57	0.007092	0.200857	AT2G47630	alpha/beta-Hydrolases	
13433982	5.37	4.72	1.57	0.0138	0.25951	AT3G27540	beta-1,4-N-	protein N-li
13370929	7.16	6.5	1.57	0.001432	0.132447	AT1G21590	adenine nu	response to
13383079	5.8	5.15	1.56	0.002894	0.156582	AT1G64380	ethylene-re	regulation c
13406466	4.26	3.62	1.56	0.033937	0.343253	AT2G47870	putative glu	cell redox h
13480813	6.42	5.78	1.56	0.022118	0.302403	PIT1	protein pitchoun 1	
13493916	8.45	7.81	1.56	0.038736	0.359741	AT4G35750	SEC14 cytos	biological_p
13532112	4.99	4.35	1.56	0.040577	0.365158	AT5G35066	hypothetica	biological_p
13437460	5.66	5.02	1.56	0.033056	0.340483	AT3G49550	hypothetica	biological_p
13535849	4.03	3.39	1.56	0.027145	0.321633	AT5G45580	homeodom	regulation c
13521497	2.56	1.93	1.56	0.023115	0.308067	AT5G03350	lectin-like p	phosphoryla
13370131	6.62	5.99	1.55	0.003525	0.164884	CLH1	chlorophyll	MAPK casca
13377980	4.09	3.45	1.55	0.015646	0.269898	AT1G50660	hypothetica	cell adhesio
13383856	3.18	2.55	1.55	0.009874	0.230134	AT1G66190	hypothetica	biological_p

13392636	3.29	2.66	1.55	0.009353	0.22494	AT2G13125	hypothetica	biological_p
13393051	6.72	6.08	1.55	0.038253	0.358491	AT2G14900	gibberellin-	response tc
13424181	5.54	4.91	1.55	0.034512	0.344747	AT3G04525		
13456419	2.91	2.28	1.55	0.033057	0.340483	AT3G45570	RING/U-box protein wit	
13459071	2.9	2.27	1.55	0.019193	0.287742	AT3G52330	F-box assoc	biological_p
13461246	9.95	9.32	1.55	0.002868	0.15583	SIP2	putative gal	response tc
13335446	4.37	3.74	1.55	0.020714	0.295178	SOM	CCCH-type	regulation c
13544615	7.16	6.53	1.55	0.00218	0.141724	DEAR2	ethylene-re	regulation c
13334945	6.6	5.98	1.54	0.018392	0.284042	BXL2	probable be	carbohydra
13375878	5.03	4.4	1.54	0.003039	0.158586	AT1G36950	RING/U-box domain-co	
13385914	2.74	2.12	1.54	0.000877	0.122048	ath-MIR858a		
13398391	10.13	9.51	1.54	0.026363	0.321058	XBAT31	putative E3	regulation c
13422994	5.75	5.12	1.54	0.023436	0.309547	AT3G01750	ankyrin rep	biological_p
13425025	2.87	2.25	1.54	0.035777	0.348799	AT3G06455	ubiquitin family protein	
13427137	5.71	5.09	1.54	0.02157	0.299337	ACT11	actin-11	gluconeoge
13462927	4.1	3.48	1.54	0.007098	0.200857	AT3G61720	Ca2+dependent plant p	
13496071	7.5	6.87	1.54	0.01847	0.284296	LAX1	auxin trans	amino acid
13404478	6.01	5.38	1.54	0.004246	0.173912	AT2G43340	hypothetica	biological_p
13377260	5.99	5.37	1.54	0.025483	0.316854	GH9C1	glycosyl hyc	carbohydra
13411580	8.35	7.73	1.54	0.016675	0.274551	PLAT2	PLAT-plant-	response tc
13491377	7.51	6.88	1.54	0.002537	0.149214	AT4G30060	core-2/I-bra	response tc
13514629	2.25	1.63	1.54	0.010349	0.234777	AT5G53720	RNA recogn	biological_p
13349879	6.14	5.53	1.53	0.04409	0.37373	AT1G49230	RING-H2 fir	protein ubi
13353869	3.17	2.56	1.53	0.004135	0.173674	AGL56	protein aga	transcriptio
13414794	3.79	3.18	1.53	0.005076	0.182225	ATCHX13	cation/H(+)	cation trans
13480872	6.7	6.08	1.53	0.010692	0.237798	RHS13	protein roo	iron ion trai
13341260	6.11	5.49	1.53	0.00258	0.149762	AT1G17230	leucine-rich	response tc
13407634	2.86	2.25	1.53	0.043795	0.373063	AT2G04050	MATE efflu:	drug transn
13340849	4.76	4.15	1.52	0.038899	0.36006	MYB58	myb domai	regulation c
13363672	6.23	5.63	1.52	0.001813	0.136255	HDG2	homeobox-	regulation c
13373216	4	3.4	1.52	0.015409	0.267587	PUP1	purine perr	purine nucl
13373970	3.73	3.13	1.52	0.001963	0.140047	AT1G30016	hypothetical protein	
13395737	3.02	2.42	1.52	0.004154	0.173674	AT2G22050	F-box/kelch	lipid transp
13400867	6.5	5.9	1.52	0.035683	0.348443	AT2G34985		
13484075	2.9	2.3	1.52	0.043842	0.373063	ath-MIR397b		
13489115	6.22	5.62	1.52	0.00598	0.19182	AT4G24780	putative pe	syncytium f
13503687	7.4	6.79	1.52	0.027032	0.321413	AT5G19730	probable pe	response tc
13505181	3.23	2.63	1.52	0.001277	0.130451	AT5G24100	Leucine-ricl	protein phc
13419012	7.92	7.32	1.52	0.019841	0.290821	MTP11	manganese	cation trans
13536399	9.33	8.72	1.52	0.00364	0.167014	LON2	lon proteas	ATP catabo
13359473	8.53	7.93	1.52	0.001613	0.133855	BBX21	putative sal	regulation c
13463350	4.52	3.91	1.52	0.028915	0.324813	AT3G62630	hypothetica	biological_p
13495360	4.72	4.12	1.52	0.013503	0.257812	AT4G38690	1-phosphat	lipid metab
13364821	5.31	4.72	1.51	0.032528	0.338778	NOT2a	NOT2 / NO	regulation c
13451583	4.96	4.36	1.51	0.010315	0.234743	AT3G20015	protein asp	proteolysis
13455039	3.25	2.66	1.51	0.009645	0.228785	VTI13	vesicle tran	intracellulai
13483523	5.18	4.59	1.51	0.023786	0.311779	AT4G12220	hypothetica	biological_p
13494073	5.74	5.15	1.51	0.008684	0.217916	AT4G35985	senescence/dehydratio	

13343563	5.41	4.82	1.51	0.004371	0.174463	AT1G22860	vacuolar so	intracellular
13359844	5.46	4.87	1.51	0.005786	0.190519	AT1G76570	chlorophyll	response tc
13406831	9.24	8.65	1.51	0.000024	0.091234	MPK17	mitogen-ac	protein phc
13407118	5.78	5.18	1.51	0.010442	0.23632	AT2G02170	Remorin fai	biological_p
13363312	8.8	9.39	-1.51	0.019291	0.287944	AT1G04680	putative pe	polysacchar
13365263	3.39	3.98	-1.51	0.008092	0.212154	THA1	threonine a	cellular ami
13393072	7.82	8.41	-1.51	0.027619	0.322798	LTP	non-specifi	lipid transp
13411987	6.13	6.73	-1.51	0.002215	0.142407	AT2G23450	wall-associa	protein phc
13482241	5.47	6.06	-1.51	0.024542	0.313451	MEKK3	MAPK/ERK	protein phc
13520566	3.59	4.19	-1.51	0.031902	0.337202	APK4	adenosine-!	sulfate assi
13540599	5.57	6.16	-1.51	0.00105	0.123978	CIPK21	CBL-interac	protein phc
13359872	2.49	3.09	-1.51	0.006968	0.199844	AT1G76600	hypothetica	respiratory
13394408	6.27	6.86	-1.51	0.001161	0.125727	HPT1	homogentis	sulfur amin
13397024	7.18	7.78	-1.51	0.009931	0.230413	AT2G25510	hypothetica	biological_p
13338103	8.62	9.23	-1.52	0.008017	0.211398	AT1G09750	aspartyl prc	proteolysis
13344694	6.51	7.11	-1.52	0.018326	0.284042	BGLU40	beta glucos	carbohydra
13358847	5.82	6.42	-1.52	0.021189	0.296896	AT1G73750	hypothetica	chlorophyll
13373541	5.74	6.34	-1.52	0.002241	0.143283	TBL38	protein tricl	biological_p
13412424	4.62	5.23	-1.52	0.018932	0.287235	AT2G24580	putative sai	tetrahydrof
13424583	4.37	4.98	-1.52	0.019415	0.288643	AT3G05400	sugar trans	transport; t
13425030	3.54	4.15	-1.52	0.004664	0.177276	AT3G06460	membrane-	sterol biosy
13433164	2.31	2.91	-1.52	0.035079	0.345865	AT3G25590	hypothetica	biological_p
13435766	5.08	5.68	-1.52	0.041297	0.366421	MLO3	MLO-like pr	defense res
13439579	7.61	8.22	-1.52	0.028547	0.324306	PUR5	phosphorib	purine nucl
13456612	2.59	3.19	-1.52	0.002431	0.146604	AT3G46020	RNA recogn	biological_p
13477178	6.98	7.58	-1.52	0.01374	0.259013	AT4G33666	hypothetica	biological_p
13348604	5.98	6.58	-1.52	0.023178	0.308143	RAP2.6	ethylene-re	regulation c
13359280	3.91	4.52	-1.52	0.031503	0.336321	AT1G74940	hypothetica	biological_p
13405201	4.66	5.26	-1.52	0.010204	0.233325	cycp3;1	cyclin p3;1	regulation c
13442051	6.1	6.71	-1.52	0.037362	0.354989	PP2-A13	phloem pro	response tc
13356841	3.61	4.22	-1.53	0.042916	0.370821	At5g57880		carbohydra
13373203	5.11	5.73	-1.53	0.002987	0.157821	AT1G28190	hypothetica	protein targ
13403967	1.79	2.4	-1.53	0.009815	0.230093	AT2G42060	cysteine/his	intracellular
13411838	3.61	4.22	-1.53	0.042916	0.370821	At5g57880		carbohydra
13412076	3.61	4.22	-1.53	0.042916	0.370821	At5g57880		carbohydra
13412890	5.6	6.22	-1.53	0.037585	0.355888	MOT1	molybdate	molybdate i
13418464	9.11	9.72	-1.53	0.000158	0.096878	CAX1	vacuolar ca	cation trans
13428893	4.69	5.3	-1.53	0.01069	0.237798	CES101	G-type lecti	protein phc
13440682	3.13	3.75	-1.53	0.025974	0.319045	AT3G57710	protein kina	protein phc
13453848	1.48	2.09	-1.53	0.01347	0.257733	CYP71B24	cytochrome	oxidation-re
13472296	1.76	2.38	-1.53	0.036463	0.351564	CRK6	cysteine-ric	protein phc
13495839	5.67	6.29	-1.53	0.045654	0.37769	AT4G39780	ethylene-re	regulation c
13516372	3.61	4.22	-1.53	0.042916	0.370821	At5g57880		carbohydra
13520543	3.61	4.22	-1.53	0.042916	0.370821	At5g57880		carbohydra
13539518	3.61	4.22	-1.53	0.042916	0.370821	At5g57880		carbohydra
13542701	3.62	4.23	-1.53	0.015852	0.270418	ARR6	two-compo	phosphorel
13351054	4.82	5.43	-1.53	0.033169	0.341032	IAA18	auxin-respc	regulation c
13461900	8.59	9.2	-1.53	0.010945	0.239907	PIL6	transcription factor PIF!	

13469203	3.3	3.9	-1.53	0.043726	0.373063	AT4G15710	hypothetica	biological_p
13410316	6.85	7.47	-1.53	0.029657	0.327695	AT2G19310	heat shock	response to
13432693	2.41	3.02	-1.53	0.00879	0.21829	AT3G24230	putative pe	biological_p
13464538	6.56	7.16	-1.53	0.001737	0.134893	RGXT2	rhamnogala	rhamnogala
13480017	1.72	2.34	-1.53	0.020075	0.292105	AT4G00390	DNA-bindin	regulation c
13511921	7.06	7.68	-1.53	0.023215	0.308342	AT5G47860	hypothetical protein	
13538783	4.81	5.43	-1.53	0.045789	0.378265	ABCG8	ABC transp	ATP catabo
13539536	7.19	7.81	-1.53	0.019378	0.288396	AT5G54940	translation	translationa
13374229	5.94	6.56	-1.54	0.014528	0.263841	AT1G30755	hypothetica	biological_p
13440020	2.38	3	-1.54	0.006899	0.199003	AT3G55890	yippee fami	fatty acid b
13448013	8.21	8.83	-1.54	0.015912	0.270418	AT3G11780	MD-2-relat	response to
13461328	4.71	5.33	-1.54	0.003163	0.158608	AT3G57780	hypothetical protein	
13479235	9.29	9.91	-1.54	0.008694	0.217916	AT4G38550	phospholip	protein targ
13486867	4.91	5.53	-1.54	0.018376	0.284042	AT4G19380	Long-chain-	oxidation-re
13497842	3.27	3.89	-1.54	0.046303	0.37953	AT5G05365	Heavy meta	metal ion tr
13506386	8.24	8.86	-1.54	0.005257	0.184607	SFP1	sugar trans	transport; r
13522884	3.94	4.57	-1.54	0.013492	0.257812	AT5G06790	hypothetica	biological_p
13526451	5.95	6.57	-1.54	0.023534	0.309983	HB30	zinc finger	homeodoma
13532568	5.8	6.42	-1.54	0.001232	0.127228	CYP81D1	cytochrome	oxidation-re
13541704	4.18	4.8	-1.54	0.040464	0.364852	AT5G60530	late embryo	transition m
13391347	4.55	5.17	-1.54	0.000541	0.109687	AT2G03810	18S pre-ribosomal asse	
13409783	4.84	5.47	-1.54	0.039697	0.362507	MCA2	protein MID	post-embry
13431883	3.75	4.38	-1.54	0.02261	0.305215	OASA2	O-acetylser	cysteine bic
13473709	5.86	6.48	-1.54	0.016029	0.270773	CGA1	putative GA	regulation c
13353323	7.71	8.34	-1.55	0.009312	0.224616	AT1G58602	probable di	defense res
13382938	5.67	6.3	-1.55	0.012512	0.252577	AT1G63860	TIR-NBS-LRI	defense res
13401525	7.22	7.85	-1.55	0.0285	0.324163	ABCG34	ABC transp	ATP catabo
13423543	8.38	9.01	-1.55	0.001682	0.133855	AT3G02910	AIG2-like (a	response to
13428707	2.33	2.97	-1.55	0.006492	0.195547	AT3G15518	hypothetica	biological_p
13467247	3.58	4.21	-1.55	0.001628	0.133855	AT4G11000	ankyrin rep	biological_p
13518095	6.37	7	-1.55	0.04225	0.369793	AT5G61880	protein trar	mRNA splici
13528865	5.35	5.99	-1.55	0.000781	0.118831	SUS1	sucrose syn	response to
13530767	1.98	2.61	-1.55	0.048377	0.386495	CIPK25	CBL-interac	protein pho
13423185	4.82	5.46	-1.55	0.002176	0.141724	PTF1	transcriptio	regulation c
13349924	3.79	4.43	-1.56	0.012553	0.252639	USPL1	BURP doma	seed develc
13372206	5.96	6.6	-1.56	0.006033	0.192256	AT1G24909	putative an	metabolic p
13414401	4.68	5.32	-1.56	0.032627	0.339011	AT2G28960	Leucine-ricl	transition m
13447102	5.39	6.03	-1.56	0.021504	0.299102	MDHAR	monodehyc	signal trans
13468226	5.32	5.96	-1.56	0.005601	0.187737	PRR2	pinoresinol	iron ion trai
13480899	10.3	10.94	-1.56	0.003791	0.167968	SAG21	senescence	MAPK casca
13349800	6.44	7.08	-1.56	0.006718	0.197793	AT1G48960	adenine nu	response to
13369913	5.27	5.92	-1.56	0.024724	0.314361	ARR7	two-compo	phosphorel
13432739	5.85	6.49	-1.56	0.00877	0.21799	MBF1C	multiprotei	transcriptio
13452792	3.32	3.96	-1.56	0.005271	0.184847	RLP38	receptor lik	defense res
13503977	5.45	6.09	-1.56	0.018074	0.283166	AT5G20400	oxidoreduc	nucleotide I
13515386	5.5	6.15	-1.56	0.018243	0.284042	AT5G55530	C2 domain-	biological_p
13356862	4.22	4.87	-1.57	0.027076	0.321413	AT1G69050	hypothetica	biological_p
13372194	5.97	6.63	-1.57	0.00314	0.158608	AT1G24909	putative an	metabolic p

13372220	5.97	6.63	-1.57	0.00314	0.158608	AT1G24909	putative an	metabolic p
13376190	4.84	5.49	-1.57	0.006473	0.19518	AT1G43910	P-loop cont	response tc
13431450	3.68	4.33	-1.57	0.011622	0.245564	AT3G21351	hypothetical protein	
13476016	9.14	9.79	-1.57	0.011127	0.241534	AT4G31351	hypothetica	biological_p
13510508	3.74	4.4	-1.57	0.000839	0.122048	AT5G44574	hypothetica	biological_p
13538661	2.78	3.44	-1.57	0.040124	0.364544	AT5G52390	PAR1 prote	biological_p
13416340	2.74	3.39	-1.57	0.016341	0.272441	RD20	caleosin 3	response tc
13346237	5.37	6.02	-1.57	0.015788	0.270373	ABCC12	multidrug r	transport; t
13384617	5.16	5.81	-1.57	0.025916	0.318512	AT1G67590	Remorin fai	biological_p
13403593	6.56	7.21	-1.57	0.00012	0.096878	CRK1	CDPK-relate	heat acclim
13458498	4.36	5.01	-1.57	0.00152	0.133855	PLP3a	phosducin-l	nuclear divi
13491057	4.88	5.53	-1.57	0.013423	0.257733	AT4G29310	hypothetica	biological_p
13513055	1.76	2.41	-1.57	0.040233	0.364725	CEP1	KDEL-tailed	proteolysis
13408065	6.52	7.18	-1.58	0.023898	0.311779	RIPK	RPM1-indu	MAPK casca
13410452	5.24	5.91	-1.58	0.018321	0.284042	AT2G19650	cysteine/his	intracellular
13418162	4.31	4.97	-1.58	0.026873	0.321413	UMAMIT12	nodulin Mtl	glucosinola
13420524	4.13	4.79	-1.58	0.00304	0.158586	AT2G42760	hypothetica	biological_p
13432101	5.31	5.97	-1.58	0.005673	0.188991	ETR2	ethylene re	phosphorel
13443189	5.07	5.73	-1.58	0.020536	0.294953	AY707472		
13482964	6.38	7.04	-1.58	0.000384	0.102587	OPT7	oligopeptid	oligopeptid
13485180	2.96	3.62	-1.58	0.046256	0.379402	AT4G15990	hypothetica	biological_p
13509547	5.85	6.51	-1.58	0.003972	0.170745	AT5G42250	alcohol deh	oxidation-re
13537086	3.57	4.24	-1.58	0.003757	0.167249	AT5G48657	defense pro	biological_p
13542083	5.71	6.37	-1.58	0.000482	0.105453	AT5G61330	rRNA proce	biological_p
13542995	5.17	5.82	-1.58	0.046475	0.380068	FLS5	flavonol syr	transition m
13347504	5.97	6.63	-1.58	0.000704	0.114662	AT1G33610	leucine-rich	signal trans
13364767	4.01	4.67	-1.58	0.01373	0.259013	ATOBGM	GTP-binding	embryo sac
13380795	6.69	7.35	-1.58	0.007467	0.205041	BCA6	beta carbor	carbon utili
13467648	2.31	2.98	-1.58	0.014906	0.265798	AT4G11910	protein STA	biological_p
13365672	4.08	4.75	-1.59	0.02342	0.309546	AT1G09460	hypothetica	cytokinesis
13419331	6.86	7.53	-1.59	0.013621	0.258684	AT2G40270	Protein kina	response tc
13498992	7.1	7.76	-1.59	0.003075	0.158608	GUS2	glucuronida	unidimensio
13514958	6.95	7.62	-1.59	0.007191	0.201586	FQR1	flavodoxin-l	response tc
13515572	3.87	4.54	-1.59	0.006274	0.194413	HSP81-3	heat shock	protein folc
13519305	5.49	6.16	-1.59	0.034265	0.344068	At5g64570		carbohydra
13361990	4.58	5.24	-1.59	0.037959	0.357756	LEA14	putative de	response tc
13485832	6.18	6.85	-1.59	0.001365	0.130717	AT4G17070	peptidyl-pro	protein folc
13342833	2.46	3.14	-1.6	0.005905	0.191511	AT1G21010	hypothetica	proline trar
13379835	7.34	8.02	-1.6	0.000462	0.105453	AT1G55450	S-adenosyl-	metabolic p
13401712	7.01	7.69	-1.6	0.02573	0.317825	AT2G36950	heavy-meta	toxin catabo
13434151	8	8.67	-1.6	0.000488	0.105453	AT3G28040	probably in	response tc
13456185	7.05	7.72	-1.6	0.006423	0.19489	XTH31	xyloglucan	cell wall ma
13483790	4.8	5.48	-1.6	0.026434	0.32111	STOMAGEN	epidermal p	cell-cell sigr
13497772	5.82	6.5	-1.6	0.005761	0.190519	AT5G05190	hypothetica	response tc
13533025	8.38	9.05	-1.6	0.005254	0.184607	GSR1	glutamine s	response tc
13404420	4.91	5.59	-1.6	0.02399	0.311779	AT2G43200	S-adenosyl-	methylation
13511375	4.81	5.49	-1.6	0.002573	0.149762	bHLH071	transcriptio	regulation c
13387223	4.67	5.34	-1.6	0.008697	0.217916	HSP101	heat shock	protein folc

13455353	8.26	8.94	-1.6	0.000937	0.122048	ERD5	proline deh	glutamate b
13457531	8.06	8.74	-1.6	0.004627	0.177052	NPC6	non-specifi	metabolic p
13483613	10.32	11	-1.6	0.040906	0.365499	AZI1	azelaic acid	protein targ
13342228	8.22	8.91	-1.61	0.035874	0.349017	BES1	protein bra	regulation c
13401680	9.48	10.17	-1.61	0.031399	0.336257	GAMMA-TI	aquaporin T	glycolysis; p
13414535	6.1	6.79	-1.61	0.009732	0.229512	GSTU5	glutathione	response to
13515584	4.88	5.57	-1.61	0.00338	0.161973	HSP81-2	heat shock	protein folc
13415260	6.28	6.97	-1.61	0.002248	0.143394	TCP10	transcriptio	MAPK casca
13336445	2.62	3.32	-1.61	0.002177	0.141724	ORA59	ethylene-re	regulation c
13452428	3.22	3.91	-1.61	0.04277	0.370821	HYR1	UDP-glycos	metabolic p
13341989	4.64	5.33	-1.62	0.035327	0.346786	WRKY61	putative WI	regulation c
13378311	6.79	7.49	-1.62	0.001961	0.140047	AT1G51805	leucine-rich	protein phc
13399502	6.97	7.66	-1.62	0.009262	0.224348	SOBIR1	leucine-rich	protein phc
13400476	6.46	7.16	-1.62	0.004816	0.178115	AT2G34170	hypothetica	biological_p
13419919	6.81	7.51	-1.62	0.002854	0.155449	ABCA1	ABC transp	protein glyc
13483539	5.68	6.37	-1.62	0.00895	0.220351	CYP706A6	cytochrome	oxidation-re
13489295	5.19	5.89	-1.62	0.01081	0.238965	MC2	metacaspas	response tc
13399845	3.29	3.99	-1.62	0.041208	0.366421	AT2G32430	putative be	protein glyc
13379351	7.08	7.78	-1.62	0.014782	0.265705	ALDH7B4	aldehyde de	response tc
13407587	4.5	5.19	-1.62	0.000844	0.122048	SOT12	sulphotrans	MAPK casca
13351770	3.77	4.47	-1.63	0.003844	0.168472	WAG1	protein WA	protein phc
13376232	6.64	7.35	-1.63	0.001975	0.140047	AAP5	amino acid	amino acid
13411817	3.69	4.4	-1.63	0.005412	0.18657	AT2G22880	VQ motif-cc	response tc
13415809	6.52	7.22	-1.63	0.002839	0.155449	AT2G32235	hypothetica	biological_p
13484275	4.93	5.64	-1.63	0.019085	0.287265	AT4G14020	Rapid alkali	biological_p
13493696	2.4	3.11	-1.63	0.035025	0.345677	HIPP25	heavy meta	metal ion tr
13508275	5.05	5.76	-1.63	0.033194	0.341169	AT5G39030	putative rec	protein phc
13541218	5.31	6.01	-1.63	0.001361	0.130717	AT5G59080	hypothetica	response to
13367598	5.84	6.54	-1.63	0.002182	0.141724	PGL1	6-phosphog	carbohydra
13352694	7.62	8.33	-1.64	0.020371	0.293959	CSLE1	cellulose sy	polysacchar
13452511	2.17	2.89	-1.64	0.012099	0.249154	AT3G22160	VQ motif-cc	protein targ
13491984	8.45	9.17	-1.64	0.000457	0.105453	CYP83B1	cytochrome	sulfur amin
13498666	5.12	5.83	-1.64	0.021898	0.301063	IQD24	IQ-domain 2	biological_p
13518585	5.81	6.52	-1.64	0.001375	0.130717	AT5G62900	hypothetical protein	
13534964	3.27	3.99	-1.64	0.000076	0.091234	AT5G43440	1-aminocyc	oxidation-re
13540967	6.76	7.47	-1.64	0.006099	0.192839	RANBP1	Ran-binding	protein imp
13545605	3.76	4.48	-1.64	0.012503	0.252577		cysteine/his	oxidation-re
13344533	5.46	6.17	-1.64	0.002303	0.144328	AT1G25520	putative tra	response tc
13386407	6.59	7.3	-1.64	0.005884	0.191511	JAZ6	protein TIFY	response tc
13502054	5.2	5.91	-1.64	0.001047	0.123978	CPuORF10	conserved p	spermine bi
13354484	5.31	6.03	-1.64	0.001553	0.133855	AT1G62790	Bifunctiona	lipid transp
13404145	5.26	5.98	-1.64	0.030397	0.331505	LSH10	hypothetica	biological_p
13448560	4.77	5.49	-1.64	0.001581	0.133855	ZIP1	zinc transp	iron ion trai
13466739	3.35	4.06	-1.64	0.004251	0.173912	AT4G09500	UDP-glycos	metabolic p
13508180	4.62	5.33	-1.64	0.028789	0.324813	AT5G38780	putative S-a	methylation
13344936	8.67	9.38	-1.65	0.01409	0.261131	AT1G27020	hypothetica	biological_p
13366447	6.53	7.25	-1.65	0.012598	0.252812	AT1G10900	1-phosphat	phosphatid
13372181	5.85	6.57	-1.65	0.003992	0.171066	AT1G24807	putative an	metabolic p

13416790	4.52	5.25	-1.65	0.020916	0.295425	CYP710A1	cytochrome	toxin catab
13418745	7.28	8	-1.65	0.018092	0.283205	AT2G38870	PR-6 protei	response tc
13495464	7.32	8.03	-1.65	0.010598	0.237798	AT4G38810	SnRK2-interacting calciu	
13535369	5.46	6.18	-1.65	0.021556	0.299337	AT5G44380	FAD-bindin	response tc
13365132	5.52	6.24	-1.65	0.036641	0.352191	AT1G08230	GABA trans	gamma-am
13361877	7.13	7.86	-1.66	0.001184	0.125727	CIPK9	CBL-interac	response tc
13376942	5.55	6.28	-1.66	0.003798	0.167968	DHNAT1	1,4-dihydro	phylloquinc
13390794	4.07	4.8	-1.66	0.00805	0.21147	GSTZ2	putative glu	aromatic ar
13421217	2.03	2.76	-1.66	0.006557	0.195757	AT2G44370	cysteine/his	oligopeptid
13427926	4.89	5.63	-1.66	0.012384	0.251485	GRF5	growth-reg	regulation c
13523114	5.12	5.85	-1.66	0.029423	0.32684	PMSR2	peptide me	cellular pro
13470788	6.73	7.47	-1.66	0.010163	0.232946	AT4G19450	major facili	biological_p
13494881	9.19	9.92	-1.66	0.014679	0.265042	XTH7	xyloglucan	carbohydra
13340367	5.76	6.5	-1.67	0.003911	0.169375	GLYI4	Lactoylgluta	carbohydra
13351887	5.33	6.07	-1.67	0.03226	0.337906	GLIP5	GDSL estera	lipid metab
13357031	5.3	6.04	-1.67	0.009068	0.221708	AT1G69520	methyltrans	metabolic p
13357611	4.55	5.29	-1.67	0.009992	0.230891	AT1G70810	Calcium-de	biological_p
13385613	5.04	5.78	-1.67	0.02297	0.307806	FH8	formin-like	mitotic cell
13447405	6.55	7.29	-1.67	0.000887	0.122048	HB2	non-symbic	fatty acid m
13454697	6.66	7.4	-1.67	0.001772	0.135544	AT3G28120	hypothetica	biological_p
13472351	6.85	7.59	-1.67	0.002922	0.156582	CRK22	cysteine-ric	protein phc
13488760	4.28	5.02	-1.67	0.000079	0.091234	AT4G24015	RING-H2 zir	biological_p
13404090	4.04	4.78	-1.67	0.002139	0.141724	AT2G42360	E3 ubiquitir	respiratory
13498037	3.05	3.78	-1.67	0.019464	0.288735	AT5G05600	oxidoreduc	response tc
13503799	5.14	5.88	-1.67	0.004371	0.174463	AT5G20000	AAA-type A	ubiquitin-de
13412418	4.35	5.09	-1.67	0.003646	0.167014	WRKY17	putative WI	regulation c
13353266	6.54	7.29	-1.68	0.008419	0.215208	AAP1	amino acid	amino acid
13375763	4.67	5.42	-1.68	0.018801	0.286726	AT1G35830	VQ motif-cc	biological_p
13500660	6.96	7.71	-1.68	0.007111	0.200857	CLT3	CRT (chloro	defense res
13464377	4.99	5.74	-1.68	0.004175	0.173762	AT4G01410	late embryc	biological_p
13362376	3.44	4.2	-1.69	0.005628	0.188378	AT1G02470	SRPBCC liga	biological_p
13391872	8.93	9.68	-1.69	0.008234	0.213864	AT2G05540	glycine-rich	protein
13393409	5.92	6.67	-1.69	0.002301	0.144328	ADF5	actin depol	biological_p
13415561	5.49	6.24	-1.69	0.005316	0.185319	AT2G31730	basic helix-l	response to
13525779	6.24	7	-1.69	0.001545	0.133855	PPH	pheophytin	chlorophyll
13530238	4.19	4.95	-1.69	0.028022	0.323299	SOS3	calcineurin	detection o
13411260	5.79	6.55	-1.69	0.000774	0.118831	AT2G21210	SAUR-like a	response tc
13505359	5.05	5.81	-1.69	0.031485	0.336303	DMR6	downy mild	MAPK casca
13472450	5.43	6.19	-1.69	0.004286	0.173912	SGT1A	phosphatas	ubiquitin-de
13458416	7.46	8.22	-1.7	0.001941	0.140047	UGT72E1	UDP-glucos	cellular ami
13517727	4.46	5.23	-1.7	0.003261	0.159634	EXO70E2	exocyst sub	response tc
13533671	1.93	2.69	-1.7	0.019661	0.289664	AGL98	protein aga	regulation c
13452690	6.15	6.92	-1.7	0.006327	0.194525	AT3G22750	protein kina	protein phc
13354379	6.31	7.08	-1.7	0.010556	0.237798	AT1G62480	vacuolar ca	calcium ion
13362342	4.09	4.86	-1.71	0.021321	0.297851	AT1G02360	putative chi	respiratory
13382084	8.31	9.08	-1.71	0.000215	0.096878	AT1G61740	Sulfite expc	protein targ
13396819	5.75	6.53	-1.71	0.004713	0.177335	AT2G24990	RIO kinase	phosphoryl
13435671	3.01	3.78	-1.71	0.027807	0.322798	AT3G44960	hypothetica	biological_p

13472707	4.37	5.14	-1.71	0.00129	0.130451	TRE1	trehalase 1	trehalose m
13488297	5.65	6.43	-1.71	0.008967	0.220371	UTR2	UDP-galact	transmemb
13511388	4.13	4.9	-1.71	0.029034	0.32532	AT5G46730	glycine-rich	biological_p
13513031	4.73	5.5	-1.71	0.009602	0.228132	WR3	high-affinity	protein targ
13545607	7.09	7.86	-1.71	0.010761	0.238442		leucine-rich	protein phc
13342238	5.51	6.28	-1.71	0.024834	0.314786	AT1G19380	hypothetica	response tc
13435387	8.07	8.85	-1.71	0.021894	0.301063	NIT1	nitrilase 1	nitrogen co
13453654	3.94	4.72	-1.71	0.012944	0.254172	AT3G25570	S-adenosylr	spermine b
13471008	2.2	2.97	-1.71	0.011877	0.247078	AT4G20000	VQ motif-cc	biological_p
13512872	8.84	9.61	-1.71	0.009443	0.225857	cpHsc70-2	heat shock	protein folc
13516661	6.52	7.29	-1.71	0.030242	0.331059	WNK4	putative sei	protein phc
13403564	6.8	7.58	-1.72	0.00982	0.230093	AT2G41090	calmodulin-	response tc
13451115	5.45	6.23	-1.72	0.005803	0.19074	AT3G19010	2-oxoglutar	protein targ
13504901	2.6	3.38	-1.72	0.027245	0.321761	AT5G23480	SWIB/MDM	DNA-depen
13447329	3.19	3.97	-1.72	0.044452	0.37471	AT3G10320	Glycosyltra	organ senes
13465479	7.04	7.82	-1.72	0.020292	0.293527	FIB	fibrillin	response tc
13397722	5.49	6.28	-1.73	0.01418	0.261997	AAO3	abscisic-ald	abscisic acid
13397876	4.98	5.77	-1.73	0.047107	0.382079	ATC	protein CEN	transition m
13455013	5.47	6.27	-1.73	0.001068	0.124366	AT3G29034	hypothetica	biological_p
13480976	8.15	8.94	-1.73	0.003194	0.158857	GSTF2	glutathione	toxin catab
13485664	2.69	3.48	-1.73	0.008032	0.211406	AT4G16790	hydroxyproline-rich gly	
13385522	7.18	7.98	-1.74	0.002014	0.141451	NRT1:2	nitrate tran	response tc
13504415	4.38	5.18	-1.74	0.035504	0.347405	NIT4	bifunctiona	nitrogen co
13539447	6.25	7.05	-1.74	0.019376	0.288396	AT5G54710	Ankyrin rep	biological_p
13495851	7.15	7.94	-1.74	0.009026	0.221256	MIPS1	myo-inositc	inositol bios
13434315	3.06	3.86	-1.74	0.035384	0.347072	AT3G28540	AAA-type ATPase family	
13483769	4.17	4.97	-1.74	0.006261	0.194413	scpl20	serine carb	proteolysis
13487754	8.26	9.06	-1.74	0.000892	0.122048	AT4G21870	heat shock	response tc
13497851	4.92	5.72	-1.74	0.003051	0.158608	LAC12	laccase 12	lignin catab
13340690	5.2	6.01	-1.75	0.013082	0.254419	WAKL6	wall associa	protein phc
13384349	4.39	5.2	-1.75	0.001909	0.139642	ath-MIR414		
13438985	5.77	6.58	-1.75	0.001788	0.135544	NAKR3	chloroplast-	metal ion tr
13491955	2.18	2.99	-1.75	0.008203	0.213728	CTF7	protein CTF	DNA repair;
13508468	4.86	5.67	-1.75	0.00036	0.102587	AT5G39785	hypothetica	translation
13439330	5.22	6.03	-1.75	0.003808	0.167968	EP3	chitinase cl	respiratory
13466291	5.33	6.13	-1.75	0.002634	0.152085	AT4G08300	nodulin MtN21 /EamA-	
13440324	5.86	6.68	-1.76	0.007174	0.201586	AT3G56880	VQ motif-cc	biological_p
13443140	8.3	9.11	-1.76	0.00019	0.096878	HIR2	SPFH/Band	protein targ
13484207	7.12	7.94	-1.76	0.000894	0.122048	CER26	HXXXD-type	glucosinola
13505230	4.81	5.63	-1.76	0.004817	0.178115	AT5G24230	lipase class	lipid metab
13419757	7.06	7.87	-1.76	0.00199	0.140191	AT2G41250	haloacid de	metabolic p
13418868	8.04	8.86	-1.76	0.00283	0.155449	AT2G39210	major facilit	amino acid
13472326	7.02	7.83	-1.76	0.013881	0.259976	CRK10	cysteine-ric	protein phc
13371655	6.67	7.49	-1.77	0.006397	0.194525	AT1G23390	F-box/kelch	biological_p
13383047	6.1	6.93	-1.77	0.003641	0.167014	CHX16	cation/H(+)	cation trans
13402233	4.95	5.77	-1.77	0.012677	0.253021	AT2G38180	GDSL estera	lipid metab
13483564	3.23	4.06	-1.77	0.001511	0.133855	Hop3	carboxylate	protein folc
13352473	5.97	6.8	-1.77	0.003176	0.158857	AT1G55265	hypothetica	biological_p

13490406	8.82	9.64	-1.77	0.045211	0.376602	ENODL2	early nodul	glucosinola
13520896	2.58	3.4	-1.77	0.004024	0.171761	LECRKA4.3	Lectin-dom	protein phc
13524896	4.24	5.07	-1.77	0.006329	0.194525	AT5G11610	Exostosin fa	biological_p
13407128	3.95	4.78	-1.78	0.016167	0.271918	PSKR1	phytosulfok	protein phc
13534578	9.32	10.15	-1.78	0.009766	0.229631	AT5G42530	hypothetica	biological_p
13363537	8.5	9.34	-1.79	0.000447	0.105453	EFE	1-aminocyc	MAPK casca
13383860	8.05	8.89	-1.79	0.005476	0.18691	GSR2	glutamine s	glycolysis; g
13386757	4.88	5.72	-1.79	0.016145	0.271918	KTI1	kunitz tryps	response tc
13477695	2.54	3.38	-1.79	0.019041	0.287235	AT4G34810	SAUR-like a	response tc
13402064	6.4	7.24	-1.79	0.029433	0.32684	AKR4C8	aldo-keto re	response tc
13470228	2.51	3.35	-1.79	0.00206	0.141724	WRKY28	WRKY DNA-	regulation c
13335058	4.28	5.13	-1.8	0.001962	0.140047	NLA	E3 ubiquitir	phosphate i
13450278	6.09	6.94	-1.8	0.043998	0.373347	SWEET16	bidirectiona	transport; b
13472766	5.27	6.13	-1.8	0.013396	0.257667	YSL1	metal-nicot	developme
13477032	5.86	6.71	-1.8	0.006225	0.194207	AT4G33420	probable pe	polyamine c
13492540	6.9	7.75	-1.8	0.024304	0.313451	KAT3	potassium c	ion transpo
13510863	4.55	5.39	-1.8	0.016162	0.271918	DUR3	urea-protor	transport; c
13463143	6.32	7.16	-1.8	0.009351	0.22494	ABCB21	ABC transpo	transport; t
13414456	5.77	6.63	-1.81	0.018155	0.283831	GLR2.7	glutamate r	transport; c
13339532	6.5	7.35	-1.81	0.001556	0.133855	CYP71B2	cytochrome	heat acclim
13537539	7.19	8.05	-1.81	0.005403	0.18652	FRO6	ferric reduc	response tc
13373698	2.3	3.16	-1.82	0.018838	0.286726	AT1G29430	SAUR-like a	response tc
13377150	4.58	5.44	-1.82	0.011962	0.247618	AT1G48690	auxin-respc	response tc
13410499	8.75	9.62	-1.82	0.000579	0.112321	MIOX2	myo-inosito	syncytium f
13488367	3.19	4.06	-1.82	0.008635	0.217916	CRK5	cysteine-ric	protein phc
13420284	5.48	6.34	-1.82	0.001152	0.125727	FBH4	transcriptio	regulation c
13478971	2.66	3.53	-1.83	0.014461	0.263617	ELI3-1	cinnamyl al	response tc
13355960	4.99	5.87	-1.84	0.014097	0.261131	AT1G66880	serine/thre	MAPK casca
13390611	4.97	5.85	-1.84	0.045696	0.377821	EMB975	pentatricop	biological_p
13390690	5.26	6.14	-1.84	0.004033	0.171761	PTR4	peptide trai	oligopeptid
13402731	6.12	6.99	-1.84	0.017881	0.281437	AT2G39400	alpha/beta-Hydrolases	
13510364	8.56	9.44	-1.84	0.002268	0.143449	GLK2	transcriptio	rRNA proce
13355579	3.77	4.65	-1.84	0.031661	0.336812	YSL7	putative me	oligopeptid
13363723	4.13	5.02	-1.85	0.00359	0.16605	ZIP5	Fe(II) transp	cation trans
13402383	7.73	8.61	-1.85	0.032788	0.339314	WRKY33	putative WI	MAPK casca
13467377	4.42	5.3	-1.85	0.01191	0.247579	ACS6	1-aminocyc	response tc
13494096	4.73	5.62	-1.85	0.002835	0.155449	J11	chaperone	protein folc
13355524	3.71	4.6	-1.86	0.000794	0.118844	AT1G65486	hypothetica	biological_p
13361730	4.83	5.72	-1.86	0.031982	0.337215	WRKY40	putative WI	MAPK casca
13383125	4.39	5.29	-1.86	0.012635	0.253021	AT1G64561	hypothetica	biological_p
13435643	8.07	8.96	-1.86	0.000636	0.114633	ACD1	pheophorbi	cell death; e
13485055	7.1	8	-1.86	0.020075	0.292105	IAGLU	UDP-glucos	metabolic p
13459715	5.88	6.78	-1.87	0.012471	0.252577	AT3G54040	PAR1 prote	transition m
13400285	3.05	3.96	-1.88	0.00707	0.200857	AT2G33510	hypothetica	biological_p
13337021	7.02	7.94	-1.88	0.029383	0.326772	MT1C	metallothio	response to
13350017	3.16	4.07	-1.88	0.023984	0.311779	AT1G49470	hypothetica	biological_p
13464121	5.71	6.62	-1.88	0.001191	0.125727	AT4G00780	TRAF-like fa	biological_p
13422750	3.23	4.16	-1.9	0.026703	0.321413	AT3G01175	hypothetica	translation;

13423581	6.54	7.46	-1.9	0.018564	0.284746	AT3G03040	F-box prote	virus induce
13463528	4.67	5.6	-1.9	0.008277	0.214021	IPT3	adenylate is	reciprocal n
13355535	4.08	5	-1.9	0.003154	0.158608	AT1G65500	hypothetica	biological_p
13367739	5.06	6	-1.91	0.004763	0.177626	AT1G14200	RING finger	protein folc
13503386	6.07	7	-1.91	0.004533	0.17629	AT5G19230	GPI-anchor	biological_p
13521118	5.36	6.29	-1.91	0.02946	0.32684	AT5G02020	hypothetica	autophagy;
13487907	5.98	6.91	-1.91	0.037166	0.354672	KT2/3	potassium c	ion transpo
13348179	4.69	5.64	-1.92	0.005712	0.189349	AT1G35710	putative leu	MAPK casca
13378261	7.4	8.34	-1.92	0.002151	0.141724	4CL1	4-coumarat	polyamine c
13467500	4.07	5.01	-1.92	0.00552	0.187161	AT4G11521	putative cys	protein phc
13484288	7.19	8.13	-1.92	0.000784	0.118831	SBP2	selenium-bi	polar nucle
13367515	3.14	4.09	-1.93	0.01458	0.264357	AT1G13520	hypothetical protein	
13437538	6.21	7.16	-1.93	0.020975	0.295425	PSK4	phytosulfok	cell prolifer
13534993	3.4	4.35	-1.94	0.000518	0.107225	UPI	Serine prot	defense res
13412428	5.31	6.28	-1.95	0.016221	0.2721	AT2G24600	Ankyrin rep	biological_p
13521782	4.36	5.32	-1.95	0.009727	0.229512	AT5G03995	hypothetica	biological_p
13414799	5.79	6.75	-1.95	0.006853	0.198648	WRKY25	WRKY trans	response tc
13335232	3.75	4.72	-1.96	0.048501	0.386889	AT1G03200	hypothetica	biological_p
13345384	6.88	7.85	-1.96	0.000504	0.106773	DPL1	Sphingosine	cellular ami
13392760	5.7	6.67	-1.96	0.046176	0.379402	SERK4	somatic em	MAPK casca
13398059	4.51	5.48	-1.96	0.029663	0.327695	AT2G28120	major facilit	transmemb
13403169	3.88	4.85	-1.96	0.00481	0.178115	AT2G40200	transcriptio	regulation c
13397590	3.57	4.55	-1.97	0.028899	0.324813	PP2-A3	phloem pro	iron ion trai
13488463	7.06	8.03	-1.97	0.000418	0.104959	CRK21	cysteine-ric	protein phc
13437325	6.01	6.99	-1.97	0.027002	0.321413	PRXCB	peroxidase	defense res
13468173	6.12	7.1	-1.98	0.000684	0.114662	AMT1;1	ammonium	response tc
13483928	3.16	4.15	-1.98	0.000875	0.122048	TPS13	terpenoid s	metabolic p
13539453	4.92	5.91	-1.98	0.003894	0.169375	AT5G54720	ankyrin rep	biological_p
13362407	4	4.99	-1.99	0.045538	0.377374	AT1G02610	RING/FYVE/PHD zinc fir	
13370805	3.67	4.66	-1.99	0.001943	0.140047	AT1G21320	nucleic acid	mRNA splic
13385930	6.85	7.84	-1.99	0.003694	0.167014	MYBL2	putative my	regulation c
13492711	6.14	7.13	-1.99	0.011605	0.245564	ATH1	homeobox	sulfur amin
13493642	6.7	7.7	-1.99	0.008771	0.21799	AT4G34950	major facilit	circadian rh
13515359	3.27	4.26	-1.99	0.000343	0.102587	AT5G55450	bifunctiona	MAPK casca
13521635	5.16	6.15	-1.99	0.002855	0.155449	AT5G03700	D-mannose	oligopeptid
13452708	4.52	5.51	-1.99	0.045684	0.377821	AT3G22800	leucine-rich repeat exte	
13479318	4.75	5.74	-1.99	0.004441	0.174463	AT4G38860	SAUR-like a	response tc
13533792	4.39	5.38	-1.99	0.011798	0.247078	UMAMIT40	nodulin MtN21 /EamA-	
13358753	6.86	7.86	-2	0.012482	0.252577	AT1G73600	putative ph	maltose me
13511274	3.97	4.97	-2	0.022469	0.304294	AT5G46490	TIR-NBS-LRI	defense res
13359693	5.27	6.28	-2.01	0.001022	0.123277	AMY2	alpha-amyl	carbohydra
13378380	5.39	6.39	-2.01	0.022021	0.302268	AT1G51860	putative LR	protein phc
13543241	7.19	8.19	-2.01	0.011642	0.245564	AT5G64100	peroxidase	response tc
13341812	5.32	6.34	-2.02	0.034424	0.344747	MYB51	myb domai	MAPK casca
13343614	7.1	8.11	-2.02	0.003386	0.161973	AT1G22890	hypothetica	biological_p
13473704	3.24	4.26	-2.02	0.001816	0.136255	AT4G26120	regulatory p	respiratory
13485028	3.85	4.86	-2.02	0.01383	0.25972	UGT84A3	putative sin	metabolic p
13487212	2.93	3.94	-2.02	0.005057	0.181828	RABE1b	putative elc	very long-cl

13371244	6.76	7.78	-2.02	0.001707	0.134204	UGT85A2	UDP-glucos	metabolic p
13381873	6.59	7.6	-2.02	0.002152	0.141724	SD1-29	G-type lecti	protein phc
13382260	6.97	7.98	-2.02	0.02631	0.320964	WRKY6	WRKY trans	respiratory
13513562	5.88	6.89	-2.02	0.000176	0.096878	ATTPPA	trehalose-p	trehalose b
13479226	5.95	6.97	-2.03	0.002178	0.141724	AT4G38540	FAD/NAD(P	metabolic p
13532524	7.62	8.64	-2.03	0.000934	0.122048	AT5G36160	tyrosine am	cellular ami
13360448	5.35	6.37	-2.03	0.006771	0.198648	SULTR1;2	sulfate tran	transition m
13444373	2.28	3.3	-2.03	0.011157	0.241646	NAC047	NAC domai	regulation c
13484549	3.92	4.94	-2.03	0.026538	0.321413	XBAT34	putative E3	MAPK casca
13351051	3.99	5.02	-2.04	0.00105	0.123978	AT1G51920	hypothetica	biological_p
13434680	5.3	6.34	-2.04	0.001015	0.123165	COBL2	COBRA-like	biological_p
13446913	7.86	8.89	-2.04	0.000706	0.114662	AT3G09440	heat shock	glucose cata
13482358	4.96	5.99	-2.04	0.00086	0.122048	AT4G08850	probable LF	protein phc
13542594	5.34	6.36	-2.04	0.000094	0.091234	HIPL2	HIPL2 prote	carbohydra
13494433	5.58	6.61	-2.04	0.006619	0.196779	PMT6	putative po	tryptophan
13377286	3.86	4.9	-2.05	0.006012	0.19195	AT1G49000	hypothetica	biological_p
13491487	4.19	5.23	-2.05	0.026941	0.321413	AT4G30250	P-loop containing nucle	
13418736	7.18	8.21	-2.05	0.007249	0.202696	YLS5	protease I (	tryptophan
13427024	7.32	8.37	-2.07	0.000659	0.114662	AT3G11930	universal st	response tc
13449344	7.02	8.07	-2.07	0.005601	0.187737	AT3G14200	chaperone	protein folc
13483617	10.74	11.79	-2.07	0.015877	0.270418	EARLI1	putative lip	protein targ
13408697	4.84	5.89	-2.07	0.000832	0.122048	ABCG5	ABC transp	stomatal co
13464862	4.65	5.7	-2.07	0.001616	0.133855	ATPMEPCRI	Probable pe	response tc
13380146	2.87	3.93	-2.08	0.034644	0.345091	AT1G55980	FAD/NAD(P	oxidation-re
13399885	5.58	6.63	-2.08	0.002567	0.149762	CSLB03	cellulose sy	polysacchar
13448447	7.99	9.04	-2.08	0.007552	0.205623	HSP70	heat shock	protein folc
13479803	5.08	6.13	-2.08	0.001901	0.139642	AT4G39955	hydrolase, a	metabolic p
13501189	4.87	5.93	-2.08	0.018026	0.283021	ZIFL1	zinc induce	response tc
13428695	2.55	3.6	-2.08	0.001591	0.133855	NAC3	ATAF-like N	MAPK casca
13355474	4.93	6	-2.1	0.000925	0.122048	PP2-A5	protein PHL	defense res
13427672	7.73	8.81	-2.11	0.000262	0.101835	Cpn60beta2	TCP-1/cpn6	protein folc
13403570	7.66	8.74	-2.12	0.002596	0.150181	TCH3	calmodulin-	protein targ
13537017	5.33	6.41	-2.12	0.000982	0.122048	AT5G48540	receptor-lik	response tc
13469191	5.26	6.36	-2.13	0.011344	0.243799	AT4G15680	monothiol g	cell redox h
13397002	2.59	3.69	-2.14	0.029671	0.327695	RLP20	receptor lik	signal trans
13405800	3.13	4.23	-2.14	0.002936	0.156582	CNGC3	cyclic nucle	ion transpo
13409950	5.04	6.14	-2.14	0.044407	0.37471	AT2G18150	peroxidase	response tc
13414431	3.66	4.76	-2.14	0.037531	0.355816	CYP707A2	abscisic acid	response tc
13359915	3.33	4.43	-2.14	0.043072	0.37171	OPR1	12-oxophyt	lipid metab
13388965	6.68	7.78	-2.15	0.005164	0.183143	AT1G78460	SOUL heme	biological_p
13368611	4.27	5.38	-2.16	0.003855	0.168472	NRAMP6	metal trans	transport; r
13393077	3.1	4.21	-2.16	0.017381	0.279014	RLP19	receptor lik	MAPK casca
13484714	4.39	5.5	-2.16	0.019616	0.289664	AT4G14746	hypothetica	biological_p
13453827	2.12	3.24	-2.17	0.018188	0.283831	CYP71B19	cytochrome	oxidation-re
13487538	6.45	7.57	-2.17	0.003075	0.158608	CRK29	cysteine-ric	protein phc
13522131	7.15	8.27	-2.17	0.022196	0.302681	NAS1	nicotianami	pollen deve
13543434	9.34	10.46	-2.18	0.000162	0.096878	XYL4	beta-D-xylo	carbohydra
13509298	5.18	6.31	-2.19	0.009051	0.221686	AT5G41761	hypothetica	biological_p

13512653	6.59	7.72	-2.19	0.016571	0.27411	CP1	Ca2+-bindir	hyperosmo
13493404	6.1	7.24	-2.19	0.00017	0.096878	CAD5	cinnamyl al	protein targ
13340261	7.38	8.52	-2.2	0.005162	0.183143	AT1G15125	S-adenosyl-	methylatior
13508296	5.86	7.01	-2.2	0.030617	0.332559	PMAT1	phenolic glu	response tc
13519279	3.45	4.6	-2.21	0.017398	0.279014	AT5G64510	hypothetica	protein fold
13441498	5.07	6.22	-2.22	0.002405	0.146331	AT3G59880	hypothetica	biological_p
13354203	4.98	6.13	-2.22	0.007623	0.206162	BGLU46	beta glucos	carbohydra
13408171	6.24	7.39	-2.22	0.002492	0.148009	PLA2-ALPHA	phospholipa	lipid metab
13342919	5.78	6.93	-2.23	0.003726	0.167043	WAK2	wall-associa	protein phc
13427730	4.44	5.59	-2.23	0.024403	0.313451	F6'H1	feruloyl CoA	toxin catab
13537000	5.83	6.99	-2.24	0.001379	0.130717	DIR1	putative lip	lipid transp
13478398	5.73	6.9	-2.25	0.002096	0.141724	UBC17	putative ub	ubiquitin-de
13354383	3.95	5.12	-2.25	0.020277	0.293527	AT1G62500	bifunctiona	lipid transp
13453844	3.34	4.52	-2.26	0.006659	0.197658	CYP71B3	cytochrome	oxidation-re
13502157	4.14	5.31	-2.26	0.014765	0.265705	AT5G16170	core-2/I-branching beta	
13386364	4.97	6.16	-2.27	0.000236	0.09768	AT1G72416	chaperone	biological_p
13438866	3.62	4.81	-2.27	0.005571	0.187737	AtCDC48B	cell division	positive reg
13469195	3.78	4.96	-2.27	0.013379	0.257667	AT4G15690	monothiol g	cell redox h
13489621	5.15	6.33	-2.27	0.001103	0.124636	ABCG4	ABC transpo	ATP catabo
13534879	1.9	3.09	-2.28	0.015618	0.269599	AT5G43285	cysteine-ric	pollen tube
13336448	4.16	5.36	-2.29	0.003434	0.163117	MYB13	myb domai	regulation c
13366267	4.73	5.93	-2.29	0.003296	0.16024	AT1G10640	pectin lyase	carbohydra
13544732	4.49	5.7	-2.3	0.000582	0.112321	ZF1	zinc-finger p	regulation c
13419041	4.35	5.56	-2.31	0.001968	0.140047	UMAMIT14	nodulin Mtl	biological_p
13485016	3.34	4.55	-2.31	0.011257	0.242386	AT4G15390	HXXXD-type	transition m
13483250	5.26	6.47	-2.32	0.014849	0.265705	OSM34	osmotin-lik	response tc
13473728	5.45	6.67	-2.33	0.000067	0.091234	ACS7	1-aminocyc	amino acid
13469353	2.97	4.19	-2.33	0.005845	0.19074	AT4G16008	hypothetica	biological_p
13396832	4.14	5.36	-2.34	0.018199	0.283831	WRKY60	putative WI	MAPK casca
13419051	4.38	5.6	-2.34	0.006365	0.194525	AT2G39518	hypothetica	biological_p
13454944	5.99	7.23	-2.35	0.013297	0.256797	AIG2	avrRpt2-ind	MAPK casca
13540961	5.84	7.08	-2.35	0.003736	0.167079	FOLK	hypothetica	biological_p
13378367	5.32	6.56	-2.36	0.000128	0.096878	AT1G51850	Leucine-ricl	protein phc
13499466	7.3	8.54	-2.36	0.001777	0.135544	AT5G08760	hypothetica	biological_p
13438070	4.78	6.02	-2.37	0.003679	0.167014	YLS2	strictosidin	biosyntheti
13428229	4.46	5.71	-2.38	0.016295	0.272238	CYP72A14	cytochrome	transition m
13482556	4.26	5.51	-2.38	0.000294	0.102237	AT4G09760	choline syn	phosphoryla
13398903	7.22	8.48	-2.39	0.018496	0.284379	UGT87A2	UDP-glycos	toxin catab
13385769	5.38	6.64	-2.4	0.003917	0.169375	CRK2	cysteine-ric	protein folc
13451739	6.1	7.36	-2.4	0.006206	0.193906	AT3G20395	RING-finger domain-cor	
13507077	4.5	5.76	-2.4	0.002098	0.141724	XGD1	xylogalactu	xylogalactu
13508897	6.02	7.28	-2.4	0.000365	0.102587	LHT1	Lysine histic	N-terminal
13505815	4.3	5.57	-2.41	0.003101	0.158608	RLP52	receptor lik	response tc
13535374	4.15	5.42	-2.41	0.001026	0.123277	AT5G44390	FAD-binding	amino acid
13451105	6.83	8.1	-2.41	0.027698	0.322798	AT3G19000	2-oxoglutar	maltose me
13490589	4.93	6.2	-2.41	0.029957	0.329463	BGLU10	beta glucos	response tc
13510657	4.77	6.04	-2.42	0.015563	0.269098	AT5G44910	Toll-Interle	oligopeptid
13413884	4.13	5.41	-2.43	0.001944	0.140047	AT2G27660	cysteine/his	respiratory

13505582	6.54	7.82	-2.43	0.031737	0.336965	AT5G25280	serine-rich	biological_p
13444098	6.78	8.06	-2.43	0.012877	0.254065	CYP89A9	cytochrome	oxidation-re
13421221	2.8	4.09	-2.44	0.000525	0.107225	AT2G44380	cysteine/his	response to
13490301	6.44	7.73	-2.44	0.002192	0.141724	AT4G27300	G-type lecti	protein pho
13519430	5.04	6.33	-2.44	0.002678	0.152554	AT5G64850	hypothetica	myo-inosito
13339861	5.07	6.37	-2.46	0.005253	0.184607	AT1G13990	hypothetica	response to
13385901	3.51	4.82	-2.47	0.000216	0.096878	AT1G70985	hydroxypro	myo-inosito
13501570	8.29	9.6	-2.47	0.001796	0.135779	FDH	formate del	metabolic p
13346465	4.19	5.5	-2.49	0.005295	0.18501	AT1G30730	FAD-binding	oxidation-re
13384115	5.21	6.54	-2.51	0.001078	0.124366	AT1G66920	putative ser	protein pho
13488714	3.92	5.25	-2.51	0.00877	0.21799	WRKY53	putative WI	MAPK casca
13536955	3.52	4.85	-2.51	0.010043	0.231867	AT5G48430	aspartyl pro	proteolysis;
13423224	2.8	4.13	-2.52	0.022154	0.302403	RGF7	hypothetica	positive reg
13453867	3.71	5.04	-2.52	0.008448	0.215414	CYP71B26	cytochrome	oxidation-re
13545581	9.24	10.59	-2.54	0.000244	0.09781		epithiospec	pentose-ph
13518894	6.68	8.03	-2.54	0.004272	0.173912	MUM2	beta-galact	carbohydra
13342060	3.91	5.26	-2.55	0.005934	0.191511	AT1G19020	hypothetica	response to
13487671	4.5	5.86	-2.56	0.017666	0.280994	NRT1.8	nitrate tran	oligopeptid
13488413	3.58	4.93	-2.56	0.00443	0.174463	CRK14	cysteine-ric	response to
13488391	2.95	4.31	-2.58	0.025829	0.318047	CRK12	putative cys	protein pho
13505219	5.2	6.57	-2.58	0.016571	0.27411	AT5G24210	lipase class	response to
13513536	3.4	4.76	-2.58	0.014852	0.265705	AT5G51440	heat shock	protein fold
13537010	3.89	5.26	-2.58	0.001497	0.133855	AT5G48530	hypothetica	biological_p
13362553	4.73	6.12	-2.62	0.007819	0.208679	GSTF6	glutathione	response to
13388333	3.5	4.9	-2.64	0.009985	0.230891	AT1G76960	hypothetica	biological_p
13452877	3.43	4.83	-2.64	0.008507	0.216228	AT3G23550	MATE efflux	drug transm
13498597	5.45	6.85	-2.64	0.004686	0.177335	WRKY26	WRKY DNA-	regulation c
13502841	4.42	5.82	-2.64	0.013354	0.25739	AT5G18030	SAUR-like a	response to
13335042	5.16	6.62	-2.75	0.037939	0.357756	BGLU11	beta glucos	carbohydra
13431668	7.4	8.86	-2.75	0.00003	0.091234	AT3G22060	Receptor-lil	response to
13346454	3.85	5.32	-2.76	0.024257	0.313226	AT1G30700	FAD-binding	amino acid
13514125	1.28	2.74	-2.76	0.01988	0.290898	AT5G52750	heavy meta	MAPK casca
13469187	3.38	4.85	-2.77	0.010349	0.234777	AT4G15670	monothiol g	cell redox h
13503391	6.21	7.68	-2.77	0.004583	0.177052	AT5G19240	GPI-anchor	tryptophan
13443268	5.98	7.45	-2.77	0.02434	0.313451	DOX1	alpha-dioxy	fatty acid al
13346462	4.81	6.29	-2.79	0.000698	0.114662	AT1G30720	FAD-binding	oxidation-re
13370749	3.85	5.33	-2.79	0.008464	0.215497	IGMT1	indole gluc	indole gluc
13486904	5.86	7.34	-2.79	0.002106	0.141724	AT4G19420	putative pe	biological_p
13485035	5.96	7.46	-2.82	0.001325	0.130717	PPDK	pyruvate, p	phosphoryl
13337929	6.07	7.57	-2.83	0.001281	0.130451	AT1G09390	GDSL estera	lipid metab
13537020	5.08	6.59	-2.84	0.002133	0.141724	ROF2	peptidyl-pro	protein pep
13522293	4.07	5.6	-2.89	0.014244	0.262491	PRX52	peroxidase	response to
13336940	4.18	5.73	-2.93	0.001076	0.124366	RLP1	receptor lik	protein fold
13382362	7.51	9.07	-2.94	0.001386	0.130717	AT1G62510	bifunctiona	lipid transp
13358162	3.46	5.02	-2.95	0.034286	0.344095	AT1G72060	serine-type	response to
13488377	5.26	6.82	-2.95	0.006453	0.195172	CRK11	cysteine-ric	protein pho
13443480	5.94	7.5	-2.95	0.007096	0.200857	WRKY45	WRKY DNA-	regulation c
13450808	2.53	4.09	-2.95	0.020157	0.292614	AT3G18250	putative me	amino acid

13469183	2.98	4.55	-2.97	0.002179	0.141724	AT4G15660	monothiol g	cell redox h
13494301	5.07	6.64	-2.97	0.000691	0.114662	AT4G36430	peroxidase	response tc
13479248	4.11	5.69	-2.98	0.003913	0.169375	AT4G38560	phospholipa	systemic ac
13534881	4.88	6.46	-2.98	0.003699	0.167014	WRKY49	putative WI	regulation c
13500637	5.84	7.42	-2.99	0.001123	0.125727	AT5G12110	elongation	translationa
13404541	5.4	6.99	-3.02	0.005445	0.18691	TI1	defensin-lik	defense res
13419748	5.88	7.48	-3.02	0.002496	0.148009	AT2G41230	hypothetica	regulation c
13515537	5.04	6.63	-3.02	0.000491	0.105453	OPT1	oligopeptid	protein targ
13534972	3.42	5.02	-3.03	0.020489	0.294953	AT5G43450	1-aminocyc	oxidation-re
13378412	3.76	5.37	-3.05	0.005008	0.181643	AT1G51890	probable LF	protein pho
13464381	4.2	5.81	-3.05	0.001192	0.125727	UMAMIT29	nodulin Mtl	cell wall mo
13383705	7.97	9.59	-3.07	0.008368	0.214904	AT1G65845	hypothetica	biological_p
13378592	5.9	7.52	-3.08	0.000882	0.122048	AT1G52200	PLAC8 fami	tryptophan
13378298	5.05	6.68	-3.09	0.000464	0.105453	AT1G51790	leucine-rich	respiratory
13485325	4.77	6.41	-3.11	0.009247	0.224186	AT4G16260	putative be	defense res
13367909	3.5	5.14	-3.12	0.045482	0.377374	PER4	peroxidase	response tc
13370081	3.31	4.98	-3.18	0.000414	0.104959	PDF1.4	defensin-lik	defense res
13451543	5.62	7.3	-3.19	0.006945	0.199533	AT3G19850	phototropic	response tc
13536042	5.1	6.77	-3.19	0.000388	0.102587	PTR3	peptide trai	oligopeptid
13404653	7.69	9.37	-3.21	0.002143	0.141724	UGT74F2	UDP-glucos	metabolic p
13357201	6.52	8.2	-3.21	0.000128	0.096878	NRT1.7	nitrate tran	oligopeptid
13441465	5.88	7.57	-3.23	0.006062	0.192523	HLECRK	lectin-recep	N-terminal
13420890	6.92	8.63	-3.28	0.009629	0.22859	AT2G43620	chitinase	respiratory
13510533	5.19	6.9	-3.28	0.002328	0.144329	AT5G44585	hypothetica	biological_p
13397006	5.14	6.86	-3.29	0.000667	0.114662	RLP21	receptor lik	signal trans
13444660	7.29	9.02	-3.31	0.001089	0.124366	PR4	pathogenes	response tc
13516820	4.83	6.59	-3.4	0.001641	0.133855	cPT4	dehydrodol	ubiquinone
13409771	2.49	4.29	-3.47	0.006329	0.194525	AT2G17740	cysteine/his	respiratory
13351025	6.55	8.37	-3.54	0.000658	0.114662	IOS1	putative leu	respiratory
13514081	3.73	5.57	-3.59	0.004238	0.173912	HSP90.1	heat shock	protein folc
13428190	6.59	8.45	-3.62	0.005662	0.188991	CYP72A8	cytochrome	circadian rh
13434268	3.07	4.94	-3.66	0.000106	0.092183	AT3G28270	hypothetica	biological_p
13533607	4.42	6.29	-3.67	0.000031	0.091234	NAC6	NAC-domai	response tc
13401632	6.19	8.08	-3.7	0.001201	0.126186	AT2G36690	2-oxoglutar	biosyntheti
13378339	6.14	8.04	-3.74	0.029929	0.329463	AT1G51820	putative LR	respiratory
13472475	4.56	6.46	-3.74	0.004205	0.173762	CORI3	cystine lyas	sulfur amin
13483621	3.56	5.47	-3.76	0.024795	0.314786	AT4G12490	Bifunctiona	lipid transp
13450196	5.87	7.79	-3.77	0.001633	0.133855	AT3G16530	legume lect	response tc
13362548	4.8	6.73	-3.81	0.002704	0.152753	GSTF7	glutathione	toxin catab
13414255	4.81	6.8	-3.99	0.007274	0.202696	KCS12	3-ketoacyl-(	fatty acid bi
13480733	4.78	6.79	-4.01	0.039574	0.36245	AT4G01870	tolB-relatec	proteolysis;
13469199	3.23	5.25	-4.06	0.001312	0.130717	AT4G15700	monothiol g	cell redox h
13530189	6.41	8.45	-4.11	0.006991	0.200221	SQE6	Squalene m	metabolic p
13410252	3.43	5.51	-4.23	0.000269	0.101835	FRK1	FLG22-indu	MAPK casca
13509850	3.73	5.86	-4.36	0.013344	0.25736	AT5G42830	HXXXD-type	acyl-transf
13500977	5.78	7.92	-4.41	0.002691	0.152554	Rap2.6L	ethylene-re	regulation c
13456701	5.34	7.65	-4.96	0.000961	0.122048	AT3G46280	protein kina	respiratory
13418852	6.57	8.94	-5.17	0.000515	0.107225	MLO12	MLO-like pr	respiratory

13533592	6.22	8.59	-5.17	0.000746	0.118831	AT5G39580	peroxidase	amino acid
13453840	4.56	6.96	-5.25	0.001751	0.1353	CYP71B23	cytochrome	response tc
13543253	8.01	10.46	-5.46	0.001582	0.133855	PRX71	peroxidase	respiratory
13375862	4.21	6.68	-5.56	0.002921	0.156582	AT1G36622	hypothetical protein	
13483625	1.85	4.36	-5.68	0.002995	0.157821	AT4G12500	bifunctiona	lipid transp
13375866	2.88	5.39	-5.69	0.029948	0.329463	AT1G36640	hypothetica	biological_p
13467770	3.98	6.73	-6.73	0.00551	0.187034	AT4G12290	copper ami	amine meta
13488063	5.13	7.93	-6.93	0.002363	0.144919	AT4G22470	protease in	amino acid
13510513	4.3	7.3	-8.01	0.001173	0.125727	AT5G44575	hypothetica	biological_p
13540185	3.91	7.38	-11.07	0.000571	0.112321	MT1B	metallothio	response to

Protein Dor	Tair ID
SWEET suga	AT5G13170
biological_process	AT2G28570
of transcript	AT2G35640
Zinc finger,	AT1G32540
Oxoglutarat	AT1G15550
Glycerophо	AT3G02040
	AT5G53048
Glycoside h	AT4G14130
SANT/Myb	AT5G49330
Zinc finger,	AT2G28510
Proteinase	AT5G47550
Oleosin	AT5G61610
SPX, N-term	AT5G20150
Peptidase C	AT4G36880
Glycoside h	AT2G36870
Homeodom	AT5G03790
Glycoside h	AT3G61490
DnaJ domai	AT3G13310
SNF2-relate	AT1G05490
Phospholipi	AT1G06520
Domain of u	AT2G45900
Glycoside h	AT1G10550
ase binding protein	AT5G47050
U box doma	AT3G52450
biological_process	AT2G18969
Carboxylest	AT5G15860
of transcript	AT1G14350
Zinc finger,	AT5G44260
Macrophag	AT3G51660
SANT/Myb	AT3G12820
Oxoglutarat	AT5G08640
SANT/Myb	AT1G01380
Homeodom	AT5G47370
Glycoside h	AT4G23500
Protein of u	AT5G03890
Bicarbonate	AT2G47160
Glycosyl tra	AT4G27480
Protein kina	AT1G08590
ponse to pho	AT4
ade; regulati	AT2G41940
Metallopho	AT4G23000
Strictosidin	AT5G22020
Peptidase S	AT5G45650
Fructose-bi	AT4G26530
Phosphate-	AT5G09440
Protease-as	AT5G19740
Zinc finger,	AT1G25250

biological_process		AT4G35070
	Metallopho	AT1G14700
	Cation efflu	AT3G58060
	EF-hand-lik	AT2G34020
	Short-chain	AT4G03140
	Ovate prot	AT4G18830
cell wall biogenesis		AT4G31590
	Peptide me	AT4G21830
	BTB/POZ	AT1G55760
	Phosphatid	AT1G21980
	Male sterili	AT5G22500
biological_process		AT5G62960
	Sterile alph	AT1G15760
	Lipase, GDS	AT4G18970
	Terpene syı	AT1G70080
	Glycoside h	AT5G57530
biological_process		AT1G54120
	Transcriptic	AT3G15270
	Phosphoen	AT4G37870
	Rubber elor	AT2G47780
	Phosphate-	AT5G64260
biological_process		AT1G67910
	Domain of ι	AT3G22810
biological_process		AT2G20835
	Glucose-me	AT3G56060
	Pectinester	AT5G53370
	Bulb-type le	AT1G78860
	Aminotrans	AT4G28410
	NAD-depen	AT4G33030
	Proteolipid	AT3G05880
	ELK; KNOX1	AT5G11060
	Protein kina	AT5G67080
	UDP-glucur	AT1G06000
	Oxoglutarat	AT3G51240
	RNA recogr	AT3G63450
	Protein-tyrc	AT1G05000
	C2 calcium-	AT4G38530
	Mitochodria	AT2G34620
	MATH	AT1G58270
	Peptidase S	AT2G22980
	Peptidase C	AT3G43960
biological_process		AT5G24880
biological_process		AT3G52870
	Pentatricop	AT5G27300
	Late embry	AT2G41990
	Short-chain	AT5G54190
	ABC transp	AT4G15236
	Aminotrans	AT2G22810

DC1; C1-like	AT2G02630
Myc-type, b	AT4G09820
Cytochrome	AT5G42650
Protein of u	AT5G47060
genesis; bio	AT5G01790
F-box doma	AT4G23580
Zinc finger,	AT1G49220
biological_process	AT1G29270
	ath-MIR167a
biological_process	AT4G01335
sucrose stir	AT5G57785
Heavy meta	AT3G24450
eterminacy;	AT4G28190
B3 DNA bin	AT3G61970
Multi antim	AT2G04070
sphorylation	AT5G58310
PMR5 N-ter	AT2G38320
Bromo adja	AT4G14140
Zinc finger,	AT4G29190
FAE1/Type	AT1G01120
Gibberellin	AT1G75750
UTP--glucos	AT3G56040
C4-dicarbox	AT4G27970
Drug/metal	AT3G56620
of programm	AT3G12920
biological_process	AT5G66800
biological_process	AT5G22460
SANT/Myb	AT2G30424
F-box doma	AT3G23880
Phosphate-	AT4G08950
Chalcone/st	AT5G13930
Myc-type, b	AT5G61270
Peptidase A	AT1G66180
Glycosyl tra	AT1G60450
Chalcone is	AT5G05270
SANT/Myb	AT5G59570
Pectinester	AT3G27980
Putative S-a	AT3G56080
SANT/Myb	AT1G56650
Myc-type, b	AT4G36930
F-box doma	AT2G44130
	ath-MIR166a
Cytochrome	AT2G46660
Glycoside h	AT4G13760
	AT4G28180
Caleosin	AT5G29560
Lipase, GDS	AT1G28570
UDP-glucur	AT2G29740

brassinoste	AT5G11070
Cytochrom	AT4G37430
Peptidase S	AT1G28110
Late embry	AT3G52470
CCT domair	AT4G27900
Haem pero:	AT4G37530
biological_process	AT5G03120
osmotic str	AT1G35516
Protein of u	AT1G69890
Transcriptic	AT4G02235
P-type ATPa	AT1G17500
Lipase, GDS	AT1G31550
Monooxyge	AT2G29720
ponse; killin	AT3G13403
Glycoside h	AT4G38990
NAD-depen	AT1G78570
Phospholipa	AT4G36945
DC1; C1-like	AT1G55380
CAP domair	AT4G25780
Peptidase S	AT4G30610
of defense re	AT4G17680
UDP-glucur	AT1G01390
Serpin dom	AT1G51330
NAD-depen	AT2G23910
Amino acid	AT2G39130
oxidative st	AT5G56550
Toll/interle	AT1G72890
	At2g23590
	AT3G60647
of transcript	AT4G30180
Oxoglutarat	AT1G52800
Protein of u	AT1G23640
Flavin mon	AT4G13260
Zinc finger,	AT1G49200
	AT1G68500
Protein of u	AT1G09157
Peptidase S	AT2G22990
	AT3G28899
Amino acid	AT1G77690
Flavin mon	AT5G43890
Glycerol-3-p	AT2G41540
B3 DNA bin	AT2G46870
SANT/Myb	AT2G30420
NPH3 doma	AT5G67440
of programm	AT1G79110
biological_process	AT1G26290
Ankyrin rep	AT1G11740
coprotein fa	AT3G52460

biological_process	AT4G23770
No apical m	AT5G18300
Glycoside h	AT4G23820
ADP/ATP ca	AT1G80300
Argonaute/	AT1G69440
LURP1-like	AT2G38640
Fatty acid h	AT1G02205
Bifunctiona	AT1G32280
osmotic str	AT1G64670
SPX, N-term	AT1G68740
transmemb	AT5G36940
Chorismate	AT1G18870
Glycoside h	AT1G11545
AP2/ERF do	AT5G25190
DNA/RNA-b	AT1G28260
Microsoma	AT1G34640
biological_process	AT2G12462
Pectinacety	AT3G05910
NB-ARC	AT3G15700
SAM depen	AT4G26460
SANT/Myb	AT4G38620
Oleosin	AT5G07571
Cyclin, C-te	AT1G70210
Fatty acid d	AT2G29980
biological_process	AT5G14090
Metallopho	AT2G16430
Homeodom	AT5G05770
Rubber elo	AT1G67360
Basic-leucir	AT4G34000
Transferase	AT5G02890
Transcriptic	AT1G60040
Cellulose sy	AT2G21770
Histone cor	AT5G10390
Transcriptic	AT1G69540
	AT5G53144
Homeodom	AT1G34650
Homeodom	AT3G18010
Bifunctiona	AT3G51600
NB-ARC	AT5G47280
	ath-MIR319b
Transcriptic	AT5G60970
Zinc finger,	AT3G61460
Protein of u	AT5G11420
biological_process	AT1G71110
Major intrir	AT1G80760
SANT/Myb	AT2G16720
Hs1pro-1, C	AT2G40000
DnaJ domai	AT1G30280

AMP-deper	AT1G65060
Fatty acid h	AT4G20870
DC1; C1-like	AT5G43030
eta-oxidatio	AT1G06550
B3 DNA bin	AT3G11580
Protein of u	AT1G67570
of programn	AT5G45100
EF-hand do	AT5G51050
Oligopeptid	AT5G53520
Glycosyl tra	AT1G32900
biological_process	AT3G32150
Aminotrans	AT1G10060
UDP-glucur	AT1G30530
	AT1G70900
Cytochrome	AT1G65670
biological_process	AT2G31850
Strictosidin	AT2G41290
Oxoglutarat	AT3G60290
Ankyrin rep	AT5G50140
Histone cor	AT5G59690
AUX/IAA pr	AT2G46530
SPX, N-term	AT4G22990
Lipase, GDS	AT3G48460
Fatty acid h	AT5G57800
AUX/IAA pr	AT3G16500
Actin-bindir	AT4G00680
gulated nuc	AT4G20880
FAE1/Type	AT5G04530
Squalene ep	AT2G22830
Histone cor	AT5G02570
Bifunctiona	AT1G73890
developme	AT3G60650
SANT/Myb	AT1G09540
Defensin-lik	AT1G35537
Alpha/beta	AT2G47630
Glycosyl tra	AT3G27540
Protein kin	AT1G21590
AP2/ERF do	AT1G64380
Glutaredoxi	AT2G47870
Zinc finger,	AT4G02075
biological_process	AT4G35750
biological_process	AT5G35066
biological_process	AT3G49550
SANT/Myb	AT5G45580
Legume lec	AT5G03350
Chlorophyll	AT1G19670
on; trichome	AT1G50660
biological_process	AT1G66190

biological_process		AT2G13125
	Gibberellin	AT2G14900
		AT3G04525
	Zinc finger,	AT3G45570
	F-box assoc	AT3G52330
	Raffinose sy	AT3G57520
	Zinc finger,	AT1G03790
	AP2/ERF do	AT5G67190
	Glycoside h	AT1G02640
	Zinc finger,	AT1G36950
		ath-MIR858a
	Ankyrin rep	AT2G28840
	Ankyrin rep	AT3G01750
	Sde2 N-terr	AT3G06455
	Actin-relate	AT3G12110
	C2 calcium-	AT3G61720
	Amino acid	AT5G01240
	Protein of u	AT2G43340
	Glycoside h	AT1G48930
	Lipoxygena	AT2G22170
	Glycosyl tra	AT4G30060
	RNA recogn	AT5G53720
	Zinc finger,	AT1G49230
	Transcriptic	AT1G60880
	Cation/H+ e	AT2G30240
	Pollen Ole e	AT4G02270
	Protein kina	AT1G17230
	Multi antim	AT2G04050
	SANT/Myb	AT1G16490
	Homeodom	AT1G05230
	Drug/metal	AT1G28230
		AT1G30016
	F-box doma	AT2G22050
		AT2G34985
		ath-MIR397b
	Pectate lyas	AT4G24780
	Pectinester	AT5G19730
	Serine-thre	AT5G24100
	Cation efflu	AT2G39450
	Peptidase S	AT5G47040
	Zinc finger,	AT1G75540
	Protein of u	AT3G62630
	Phospholipa	AT4G38690
	NOT2/NOT3	AT1G07705
	Peptidase A	AT3G20015
	Vesicle tran	AT3G29100
biological_process		AT4G12220
	Senescence	AT4G35985

Citron-like;	AT1G22860
Chlorophyll	AT1G76570
Protein kina	AT2G01450
Remorin, C-	AT2G02170
Pectate lyas	AT1G04680
Aromatic ar	AT1G08630
Bifunctiona	AT2G15050
Protein kina	AT2G23450
Protein kina	AT4G08470
Adenylylsul	AT5G67520
Protein kina	AT5G57630
Protein of u	AT1G76600
UbiA prenyl	AT2G18950
biological_process	AT2G25510
Peptidase A	AT1G09750
Glycoside h	AT1G26560
catabolic pr	AT1G73750
PMR5 N-ter	AT1G29050
FAD depend	AT2G24580
General sub	AT3G05400
GNS1/SUR4	AT3G06460
biological_process	AT3G25590
Mlo-related	AT3G45290
AIR synthas	AT3G55010
RNA recogn	AT3G46020
biological_process	AT4G33666
AP2/ERF do	AT1G43160
Protein of u	AT1G74940
Cyclin PHO8	AT2G45080
Phloem pro	AT3G61060
te metaboli	At5g57880
Protein of u	AT1G28190
C1-like	AT2G42060
te metaboli	At5g57880
te metaboli	At5g57880
ion transpor	At2g25680
Sodium/cal	AT2G38170
Protein kina	AT3G16030
Protein kina	AT3G57710
Cytochrome	AT3G26230
Protein kina	AT4G23140
AP2/ERF do	AT4G39780
te metaboli	At5g57880
te metaboli	At5g57880
te metaboli	At5g57880
Signal trans	AT5G62920
AUX/IAA pr	AT1G51950
Myc-type, b	AT3G59060

biological_process	AT4G15710
› oxidative st	AT2G19310
Pectate lyas	AT3G24230
Nucleotide-	AT4G01750
Protein of u	AT4G00390
Protein of u	AT5G47860
ABC transp	AT5G52860
Translation	AT5G54940
Protein of u	AT1G30755
Yippee-like	AT3G55890
MD-2-relat	AT3G11780
	AT3G57780
Phospholip	AT4G38550
Glucose-me	AT4G19380
Heavy meta	AT5G05365
General sub	AT5G27350
Protein of u	AT5G06790
ZF-HD hom	AT5G15210
Cytochrom	AT5G36220
Root cap	AT5G60530
mbly protei	AT2G03810
Uncharacte	AT2G17780
Tryptophan	AT3G22460
Zinc finger,	AT4G26150
NB-ARC	AT1G58602
Toll/interle	AT1G63860
ABC transp	AT2G36380
AIG2-like	AT3G02910
biological_process	AT3G15518
Ankyrin rep	AT4G11000
ing, via splic	AT5G61880
Sucrose syn	AT5G20830
Protein kin	AT5G25110
Transcriptic	AT3G02150
BURP doma	AT1G49320
Glutamine	AT1G24909
Protein kin	AT2G28960
Pyridine nu	AT3G09940
NmrA-like	AT4G13660
Late embry	AT4G02380
UspA	AT1G48960
Signal trans	AT1G19050
Helix-turn-h	AT3G24500
Leucine-rich	AT3G23120
Oxoglutara	AT5G20400
C2 calcium-	AT5G55530
biological_process	AT1G69050
Glutamine	AT1G24909

Glutamine a	AT1G24909
ATPase, AA	AT1G43910
	AT3G21351
biological_process	AT4G31351
biological_process	AT5G44574
PAR1	AT5G52390
Caleosin	AT2G33380
ABC transpo	AT1G30410
Remorin, C-	AT1G67590
Protein kina	AT2G41140
Thioredoxin	AT3G50960
Protein of u	AT4G29310
Peptidase C	AT5G50260
Serine-thre	AT2G05940
DC1; C1-like	AT2G19650
Drug/metal	AT2G37460
Protein of u	AT2G42760
Signal trans	AT3G23150
	AY707472
Oligopeptid	AT4G10770
biological_process	AT4G15990
Alcohol deh	AT5G42250
Pathogenic	AT5G48657
Apoptosis-a	AT5G61330
Oxoglutarat	AT5G63600
Leucine-rich	AT1G33610
Domain of u	AT1G07620
Carbonic an	AT1G58180
Staygreen p	AT4G11910
X8	AT1G09460
Serine-thre	AT2G40270
Glycoside h	AT5G07830
NADPH-dep	AT5G54500
Heat shock	AT5G56010
te metabolic	At5g64570
Late embry	AT1G01470
Cyclophilin-	AT4G17070
Protein of u	AT1G21010
Methyltran	AT1G55450
Heavy meta	AT2G36950
Protein kina	AT3G28040
Glycoside h	AT3G44990
naling; stom	AT4G12970
Protein of u	AT5G05190
Glutamine s	AT5G37600
Putative S-a	AT2G43200
Myc-type, b	AT5G46690
ATPase, AA	AT1G74310

Proline deh	AT3G30775
Phosphoest	AT3G48610
Bifunctiona	AT4G12470
BZR1, trans	AT1G19350
Major intrir	AT2G36830
oxidative st	AT2G29450
Heat shock	AT5G56030
Transcriptic	AT2G31070
AP2/ERF do	AT1G06160
UDP-glucur	AT3G21760
DNA-bindin	AT1G18860
Protein kin	AT1G51805
Protein kin	AT2G31880
Protein of u	AT2G34170
ABC transp	AT2G41700
Cytochrom	AT4G12320
Zinc finger,	AT4G25110
Glycosyl tra	AT2G32430
Aldehyde d	AT1G54100
Sulfotransf	AT2G03760
Protein kin	AT1G53700
Amino acid	AT1G44100
VQ	AT2G22880
process; resp	AT2G32235
Rapid ALkal	AT4G14020
Heavy meta	AT4G35060
Serine-thre	AT5G39030
oxidative st	AT5G59080
Glucosamin	AT1G13700
Cellulose sy	AT1G55850
VQ	AT3G22160
Cytochrom	AT4G31500
IQ motif, EF	AT5G07240
Protein of u	AT5G62900
Oxoglutarat	AT5G43440
Ran binding	AT5G58590
DC1; Prenylated rab acceptor PRA1; C1-like	
Uncharacte	AT1G25520
Tify; CO/CO	AT1G72450
S-adenosyl-	AT5G15948
Bifunctiona	AT1G62790
Domain of u	AT2G42610
Zinc/iron p	AT3G12750
UDP-glucur	AT4G09500
SAM depen	AT5G38780
Domain of u	AT1G27020
Phosphatid	AT1G10900
Glutamine	AT1G24807

Cytochrom	AT2G34500
Proteinase	AT2G38870
um sensor S	AT4G38810
FAD linked	AT5G44380
Amino acid	AT1G08230
Protein kin	AT1G01140
Thioesteras	AT1G48320
Glutathion	AT2G02380
DC1; C1-lik	AT2G44370
WRC; Gluta	AT3G13960
Peptide me	AT5G07460
Nodulin-lik	AT4G19450
Glycoside h	AT4G37800
Glyoxalase-	AT1G15380
Lipase, GDS	AT1G53920
Methyltran	AT1G69520
C2 calcium-	AT1G70810
Actin-bindi	AT1G70140
Globin	AT3G10520
biological_process	AT3G28120
Serine-thre	AT4G23300
Zinc finger,	AT4G24015
Zinc finger,	AT2G42360
Oxoglutara	AT5G05600
ATPase, AA	AT5G20000
DNA-bindin	AT2G24570
Amino acid	AT1G58360
VQ	AT1G35830
sponse to oo	AT5G12170
Late embry	AT4G01410
Streptomyc	AT1G02470
Glycine rich	AT2G05540
Actin-bindi	AT2G16700
ethylene st	AT2G31730
catabolic pr	AT5G13800
EF-hand-lik	AT5G24270
Auxin respo	AT2G21210
Oxoglutara	AT5G24530
Tetratricop	AT4G23570
UDP-glucur	AT3G50740
Exocyst con	AT5G61010
Transcriptic	AT5G39810
Serine-thre	AT3G22750
transport; (	AT1G62480
Glycoside h	AT1G02360
Transmemb	AT1G61740
RIO-like kin	AT2G24990
biological_process	AT3G44960

Glycoside h	AT4G24040
UAA transp	AT4G23010
biological_process	AT5G46730
geting to me	AT5G50200
Serine-threonine/tyrosine-protein kinase catalytic domain; Leucine-rich repeat; Malectin; Leucine rich repe	
Protein of u	AT1G19380
Carbon-nitr	AT3G44310
S-adenosyl	AT3G25570
VQ	AT4G20000
Heat shock	AT5G49910
Protein kin	AT5G58350
EF-hand do	AT2G41090
Oxoglutara	AT3G19010
SWIB/MDM	AT5G23480
Glycosyltra	AT3G10320
Plastid lipid	AT4G04020
Aldehyde o	AT2G27150
Phosphatid	AT2G27550
biological_process	AT3G29034
Glutathione	AT4G02520
Protein of u	AT4G16790
Proton-dep	AT1G69850
Carbon-nitr	AT5G22300
Ankyrin rep	AT5G54710
Myo-inosito	AT4G39800
ATPase, AA	AT3G28540
Peptidase S	AT4G12910
Alpha cryst	AT4G21870
Multicoppe	AT5G05390
Protein kin	AT1G16110
	ath-MIR414
Heavy meta	AT3G53530
: sister chro	AT4G31400
Protein of u	AT5G39785
Glycoside h	AT3G54420
Drug/metal	AT4G08300
VQ	AT3G56880
Band 7 prot	AT3G01290
Transferase	AT4G13840
Lipase, clas	AT5G24230
HAD-like do	AT2G41250
Nodulin-lik	AT2G39210
Protein kin	AT4G23180
F-box doma	AT1G23390
Cation/H+ e	AT1G64170
Lipase, GDS	AT2G38180
Tetratricop	AT4G12400
Protein of u	AT1G55265

Plastocyani	AT4G27520
Protein kina	AT5G01560
Exostosin-li	AT5G11610
Protein kina	AT2G02220
biological_process	AT5G42530
Oxoglutarat	AT1G05010
Glutamine s	AT1G66200
Proteinase	AT1G73260
Auxin respo	AT4G34810
NADP-depe	AT2G37760
DNA-bindin	AT4G18170
SPX, N-term	AT1G02860
SWEET suga	AT3G16690
Oligopeptid	AT4G24120
Haem pero:	AT4G33420
Cyclic nucle	AT4G32650
Sodium/sol	AT5G45380
ABC transpo	AT3G62150
Ionotropic g	AT2G29120
Cytochrome	AT1G13080
FAD-binding	AT5G49730
Auxin respo	AT1G29430
GH3 auxin-r	AT1G48690
Inositol oxy	AT2G19800
Serine-thre	AT4G23130
Myc-type, b	AT2G42280
Alcohol deh	AT4G37980
Protein kina	AT1G66880
Pentatricop	AT2G01860
Proton-dep	AT2G02020
Alpha/beta	AT2G39400
SANT/Myb	AT5G44190
Oligopeptid	AT1G65730
Zinc/iron pe	AT1G05300
DNA-bindin	AT2G38470
Aminotrans	AT4G11280
DnaJ domai	AT4G36040
biological_process	AT1G65486
DNA-bindin	AT1G80840
biological_process	AT1G64561
Pheophorbi	AT3G44880
UDP-glucur	AT4G15550
PAR1	AT3G54040
biological_process	AT2G33510
nitrate; nit	AT1G07610
Protein of u	AT1G49470
MATH	AT4G00780
Protein of u	AT3G01175

F-box doma	AT3G03040
tRNA isope	AT3G63110
biological_process	AT1G65500
Zinc finger,	AT1G14200
biological_process	AT5G19230
response tc	AT5G02020
Cyclic nucle	AT4G22200
Protein kin	AT1G35710
AMP-deper	AT1G51680
Gnk2-homc	AT4G11521
Selenium-b	AT4G14040
Protein of u	AT1G13520
Phytosulfok	AT3G49780
Proteinase	AT5G43580
Ankyrin rep	AT2G24600
biological_process	AT5G03995
DNA-bindin	AT2G30250
biological_process	AT1G03200
Pyridoxal pl	AT1G27980
Protein kin	AT2G13790
Nodulin-like	AT2G28120
Myc-type, b	AT2G40200
AIG1; Phloe	AT2G26820
Protein kin	AT4G23290
Haem pero:	AT3G49120
Ammonium	AT4G13510
Terpene syr	AT4G13300
Ankyrin rep	AT5G54720
Zinc finger,	AT1G02610
RNA recogn	AT1G21320
SANT/Myb	AT1G71030
POX; Home	AT4G32980
Nodulin-like	AT4G34950
Bifunctiona	AT5G55450
S-locus glyc	AT5G03700
Leucine-rich	AT3G22800
Auxin respo	AT4G38860
Drug/metal	AT5G40240
Methyltran	AT1G73600
Toll/interle	AT5G46490
Glycosyl hy	AT1G76130
Serine-thre	AT1G51860
Haem pero:	AT5G64100
SANT/Myb	AT1G18570
biological_process	AT1G22890
BTB/POZ; A	AT4G26120
UDP-glucur	AT4G15490
Elongation	AT4G20360

UDP-glucur	AT1G22360
S-locus glyc	AT1G61380
DNA-bindin	AT1G62300
Trehalose-p	AT5G51460
Monooxyge	AT4G38540
Aminotrans	AT5G36160
STAS domai	AT1G78000
No apical m	AT3G04070
Ankyrin rep	AT4G14365
biological_process	AT1G51920
Glycosyl-ph	AT3G29810
Heat shock	AT3G09440
Protein kina	AT4G08850
Glucose/So	AT5G62630
General sub	AT4G36670
biological_process	AT1G49000
ATPase, AA	AT4G30250
ThiJ/PfpI	AT2G38860
UspA	AT3G11930
DnaJ domai	AT3G14200
Bifunctiona	AT4G12480
ABC transpo	AT2G13610
Pectinester	AT4G02330
eduction pro	AT1G55980
Cellulose sy	AT2G32530
Heat shock	AT3G12580
metabolic process	AT4G39955
Major facili	AT5G13750
No apical m	AT3G15500
Toll/interle	AT1G65390
Chaperonin	AT3G13470
EF-hand-lik	AT2G41100
Gnk2-homo	AT5G48540
Glutaredoxi	AT4G15680
Leucine-rich	AT2G25440
Ion transpo	AT2G46430
Haem pero:	AT2G18150
Cytochrome	AT2G29090
NADH:flavir	AT1G76680
SOUL haem	AT1G78460
Natural resi	AT1G15960
Leucine-rich	AT2G15080
biological_process	AT4G14746
Cytochrome	AT3G26170
Protein kina	AT4G21410
Nicotianam	AT5G04950
Glycoside h	AT5G64570
biological_process	AT5G41761

EF-hand-like	AT5G49480
Alcohol deh	AT4G34230
SAM depen	AT1G15125
Transferase	AT5G39050
ling; ER-nucl	AT5G64510
biological_process	AT3G59880
Glycoside h	AT1G61820
Phospholipa	AT2G06925
Protein kina	AT1G21270
Oxoglutarat	AT3G13610
Bifunctiona	AT5G48485
Ubiquitin-co	AT4G36410
lipid transport	AT1G62500
Cytochrome	AT3G26220
Glycosyl tra	AT5G16170
DnaJ domai	AT1G72416
CDC48, N-te	AT3G53230
Glutaredoxi	AT4G15690
ABC transpo	AT4G25750
guidance; d	AT5G43285
SANT/Myb	AT1G06180
Glycoside h	AT1G10640
of transcript	AT5G67450
Drug/metal	AT2G39510
Transferase	AT4G15390
Thaumatin	AT4G11650
Aminotrans	AT4G26200
biological_process	AT4G16008
DNA-bindin	AT2G25000
Uncharacte	AT2G39518
AIG2-like	AT3G28930
Phosphatid	AT5G58560
Protein kina	AT1G51850
biological_process	AT5G08760
Strictosidin	AT3G51430
Cytochrome	AT3G14680
ation; glycer	AT4G09760
UDP-glucur	AT2G30140
Protein kina	AT1G70520
Zinc finger,	AT3G20395
Exostosin-li	AT5G33290
Amino acid	AT5G40780
Leucine-rich	AT5G25910
FAD linked	AT5G44390
Oxoglutarat	AT3G19000
Glycoside h	AT4G27830
Toll/interle	AT5G44910
DC1; C1-like	AT2G27660

biological_process	AT5G25280
Cytochrom	AT3G03470
DC1; C1-lik	AT2G44380
S-locus glyc	AT4G27300
l hexakisph	AT5G64850
Protein of u	AT1G13990
l hexakisph	AT1G70985
D-isomer s	AT5G14780
FAD linked	AT1G30730
Serine-thre	AT1G66920
DNA-bindin	AT4G23810
oligopeptid	AT5G48430
ulation of c	AT3G02240
Cytochrom	AT3G26290
Lipase, GDSL	
Glycoside h	AT5G63800
oxidative s	AT1G19020
Proton-dep	AT4G21680
Protein kin	AT4G23220
Serine-thre	AT4G23200
Lipase, clas	AT5G24210
Alpha cryst	AT5G51440
biological_process	AT5G48530
Glutathion	AT1G02930
biological_process	AT1G76960
Multi antim	AT3G23550
DNA-bindin	AT5G07100
Auxin respo	AT5G18030
Glycoside h	AT1G02850
Gnk2-homo	AT3G22060
FAD linked	AT1G30700
Heavy meta	AT5G52750
Glutaredoxi	AT4G15670
catabolic pr	AT5G19240
Haem pero	AT3G01420
FAD linked	AT1G30720
O-methyltr	AT1G21100
Pectinacety	AT4G19420
PEP-utilisin	AT4G15530
Lipase, GDS	AT1G09390
Peptidyl-pr	AT5G48570
Haem pero	AT5G05340
Leucine-ric	AT1G07390
Bifunctiona	AT1G62510
Proteinase	AT1G72060
Serine-thre	AT4G23190
DNA-bindin	AT3G01970
import; biol	AT3G18250

Glutaredoxi	AT4G15660
Haem pero:	AT4G36430
Phospholipa	AT4G38560
DNA-bindin	AT5G43290
Glutathione	AT5G12110
Scorpion lo	AT2G43510
of cell growt	AT2G41230
Oligopeptid	AT5G55930
Oxoglutarat	AT5G43450
Protein kina	AT1G51890
Drug/metal	AT4G01430
biological_process	AT1G65845
Uncharacte	AT1G52200
Serine-thre	AT1G51790
Glycoside h	AT4G16260
Haem pero:	AT1G14540
Gamma thi	AT1G19610
BTB/POZ; N	AT3G19850
Proton-dep	AT5G46050
UDP-glucur	AT2G43820
Proton-dep	AT1G69870
Protein kina	AT3G59700
Glycoside h	AT2G43620
biological_process	AT5G44585
Leucine-ricl	AT2G25470
Chitin-bindi	AT3G04720
Decaprenyl	AT5G58770
C1-like	AT2G17740
Protein kina	AT1G51800
Heat shock	AT5G52640
Cytochrome	AT3G14620
Protein of u	AT3G28270
No apical m	AT5G39610
Oxoglutarat	AT2G36690
Protein kina	AT1G51820
Aminotrans	AT4G23600
Bifunctiona	AT4G12490
Legume lec	AT3G16530
Glutathione	AT1G02920
FAE1/Type	AT2G28630
Peptidase S	AT4G01870
Glutaredoxi	AT4G15700
FAD depend	AT5G24160
Protein kina	AT2G19190
Transferase	AT5G42830
AP2/ERF do	AT5G13330
Malectin-lik	AT3G46280
Mlo-relatec	AT2G39200

Haem pero:	AT5G39580
Cytochrom	AT3G26210
Haem pero:	AT5G64120
	AT1G36622
Bifunctiona	AT4G12500
biological_process	AT1G36640
Copper ami	AT4G12290
transport; li	AT4G22470
biological_process	AT5G44575
response to metal ion	AT5G56795

ne rich repeat 4
